# Supplementary material for: Highly selective transition-metal-free transamidation of amides and amidation of esters at room temperature
Source: Nat Commun. 2018 Oct 9;9:4165. doi: 10.1038/s41467-018-06623-1 (PMC6178361; doi:10.1038/s41467-018-06623-1)
Supplement: Supplementary file 1 — Supplementary Information [file 41467_2018_6623_MOESM1_ESM.pdf]

Highly Selective Transition-Metal-Free Transamidation of Amides and Amidation of Esters at Room Temperature

Li et al.

Supplementary Methods

List of Known Compounds/General Methods

All starting materials reported in the manuscript have been previously described in literature and prepared by the method reported previously. Amides and esters were prepared by standard methods.[1-26] All products reported in the manuscript have been previously described in literature.[27-48] All experiments were performed using standard Schlenk techniques under argon or nitrogen atmosphere unless stated otherwise. All solvents were purchased at the highest commercial grade and used as received or after purification by passing through activated alumina columns or distillation from sodium/benzophenone under nitrogen. All other chemicals were purchased at the highest commercial grade and used as received. Reaction glassware was oven-dried at 140 °C for at least 24 h or flame-dried prior to use, allowed to cool under vacuum and purged with argon or nitrogen (three cycles). All products were identified using ^{1}H NMR analysis and comparison with authentic samples. GC and/or GC/MS analysis was used for volatile products. All yields refer to yields determined by ^{1}H NMR and/or GC or GC/MS using an internal standard (optimization) and isolated yields (preparative runs) unless stated otherwise. ^{1}H NMR and ^{13}C NMR spectra were recorded in $CDCl_3$ on Bruker spectrometers at 500 (^{1}H NMR) and 125 MHz (^{13}C NMR). All shifts are reported in parts per million (ppm) relative to residual $CHCl_3$ peak (7.27 and 77.2 ppm, ^{1}H NMR and ^{13}C NMR, respectively). All coupling constants (J) are reported in hertz (Hz). Abbreviations are: s, singlet; d, doublet; t, triplet; q, quartet; brs, broad singlet. GC-MS chromatography was performed using Agilent HP6890 GC System and Agilent 5973A inert XL EI/CI MSD using helium as the carrier gas at a flow rate of 1 mL/min and an initial oven temperature of 50 °C. The injector temperature was 280 °C. The detector temperature was 280 °C. For runs with the initial oven temperature of 50 °C, temperature was increased with a 10 °C/min ramp after 50 °C hold for 3 min to a final temperature of 280 °C, then hold at 280 °C for 10 min (splitless mode of injection, total run time of 33.00 min). All flash chromatography was performed using silica gel, 60 Å, 300 mesh. TLC analysis was carried out on glass plates coated with silica gel 60 F254, 0.2 mm thickness. The plates were visualized using a 254 nm ultraviolet lamp or aqueous potassium permanganate solutions. ^{1}H NMR and ^{13}C NMR data are given for all compounds for characterization purposes. ^{1}H NMR, ^{13}C NMR, Mp and HRMS data are reported for all new compounds.

Experimental Procedures and Characterization Data

General Procedure for Transamidation and Amidation Reactions. An oven-dried vial equipped with a stir bar was charged with an amide or ester substrate (neat, 1.0 equiv), amine (typically, 2.0 equiv) placed under a positive pressure of argon, and subjected to three evacuation/backfilling cycles. Toluene (typically, 0.25 M) and LiHMDS (1.0 M in THF, typically, 3.0 equiv) were sequentially added with vigorous stirring at room temperature, and the reaction mixture was stirred at room temperature for an indicated time. After the indicated time, the reaction mixture was quenched with NH_4Cl (aq., 1.0 M, 1 mL), diluted with CH_2Cl_2 (10 mL), the organic layer was washed with water (1 x 10 mL), brine (1 x 10 mL), dried and concentrated. A sample was analyzed by ^{1}H NMR ($CDCl_3$, 500 MHz) and GC-MS to obtain conversion, yield and selectivity using internal standard and comparison with authentic samples. Purification by chromatography on silica gel (EtOAc/hexanes) afforded the title product.

Representative Procedure for Transamidation of Amides. Gram Scale. Ph/Boc Amide. An oven-dried vial equipped with a stir bar was charged with *tert*-butyl benzoyl(phenyl)carbamate (1.00 g, 3.36 mmol, 1.0 equiv), *p*-anisidine (0.829 g, 6.73 mmol, 2.0 equiv) placed under a positive pressure of argon, and subjected to three evacuation/backfilling cycles. Toluene (13.5 mL) and LiHMDS (1.0 M in THF, 10.1 mL, 3.0 equiv) were sequentially added with vigorous stirring at -20 °C, the reaction mixture was slowly warmed up to room temperature and stirred for 15 h at room temperature. After the indicated, the reaction mixture was quenched with NH_4Cl (aq., 1.0 M, 20 mL), extracted with EtOAc (3 x 50 mL), the organic layers were combined, washed with water (1 x 50 mL), brine (1 x 50 mL), dried over Na_2SO_4 and concentrated. Purification by chromatography on silica gel (EtOAc/hexanes) afforded the title product. Yield 94% (720 mg). White solid. Characterization data are included in the section below.

Representative Procedure for Transamidation of Amides. Gram Scale. Me/Boc Amide. An oven-dried vial equipped with a stir bar was charged with *tert*-butyl benzoyl(methyl)carbamate (1.00 g, 4.25 mmol, 1.0 equiv), aniline (0.791 g, 8.50 mmol, 2.0 equiv) placed under a positive pressure of argon, and subjected to three evacuation/backfilling cycles. Toluene (17 mL) and

LiHMDS (1.0 M in THF, 12.8 mL, 3.0 equiv) were sequentially added with vigorous stirring at 0 °C, the reaction mixture was slowly warmed up to room temperature and stirred for 15 h at room temperature. After the indicated, the reaction mixture was quenched with NH_4Cl (aq., 1.0 M, 20 mL), extracted with EtOAc (3 x 50 mL), the organic layers were combined, washed with water (1 x 50 mL), brine (1 x 50 mL), dried over Na_2SO_4 and concentrated. Purification by chromatography on silica gel (EtOAc/hexanes) afforded the title product. Yield 91% (770 mg). White solid. Characterization data are included in the section below.

Representative Procedure for Amidation of Esters. Gram Scale. An oven-dried vial equipped with a stir bar was charged with phenyl benzoate (1.00 g, 5.05 mmol, 1.0 equiv), aniline (0.940 g, 10.10 mmol, 2.0 equiv) placed under a positive pressure of argon, and subjected to three evacuation/backfilling cycles. Toluene (20 mL), LiHMDS (1.0 M in THF, 15.1 mL, 3.0 equiv) were sequentially added with vigorous stirring at room temperature, and the reaction mixture was stirred for 15 h at room temperature. After the indicated time, the reaction mixture was quenched with NH_4Cl (aq., 1.0 M, 20 mL), extracted with EtOAc (3 x 50 mL), the organic layers were combined, washed with water (1 x 50 mL), brine (1 x 50 mL), dried over Na_2SO_4 and concentrated. Purification by chromatography on silica gel (EtOAc/hexanes) afforded the title product. Yield 90% (895 mg). White solid. Characterization data are included in the section below.

Mechanistic Studies Referred to from the Main Manuscript

A series of studies were performed to gain insight into the reaction mechanism and investigate factors involved in controlling the transition-metal-free transamidation protocol.

(1) Intermolecular competition experiments revealed that the electronic nature of aniline does not significantly affect the reactivity, however, a trend favoring electron-deficient anilines should be noted (Supplementary Figure 1).

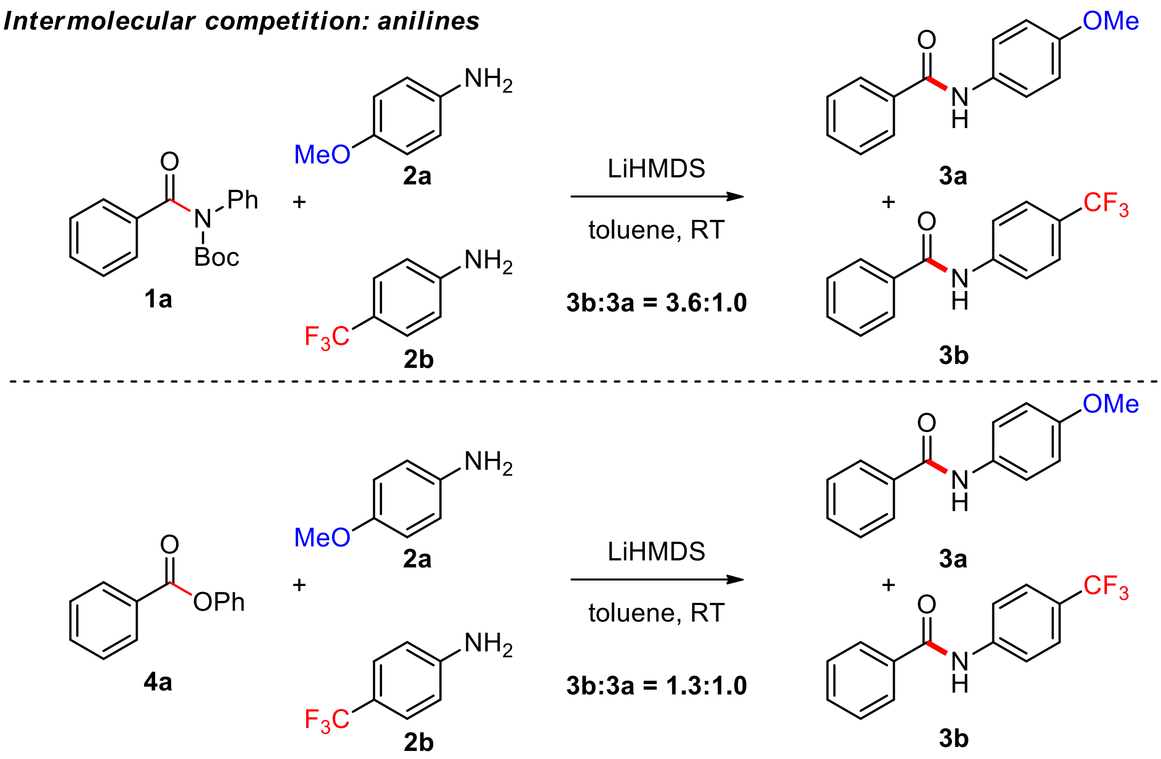

Supplementary Figure 1. Intermolecular Competition Experiments: Anilines. *Conditions:* ***1*** *or* ***4*** *(1.0 equiv), aniline (1.0 equiv each), LiHMDS (3.0 equiv), toluene (0.25 M), 23 °C, 15 h.*

(2) Furthermore, intermolecular competition experiments revealed that electron-deficient amides and esters are inherently more reactive than their electron-rich counterparts (Supplementary Figure 2).

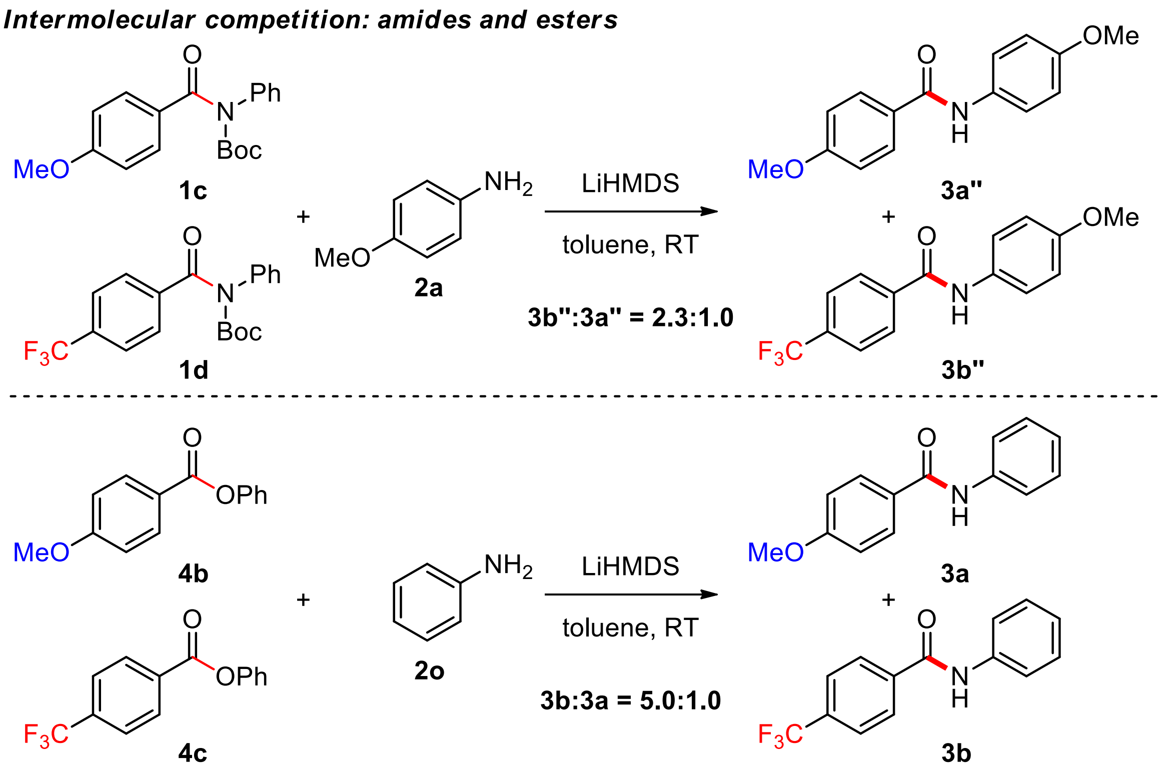

Supplementary Figure 2. Intermolecular Competition Experiments: Amides and Esters. *Conditions: **1** or **4** (1.0 equiv each), aniline (1.0 equiv), LiHMDS (3.0 equiv), toluene (0.25 M), 23 °C, 15 h.*

(3) Further competition experiments established a similar order of reactivity of amides and esters (Supplementary Figure 3).

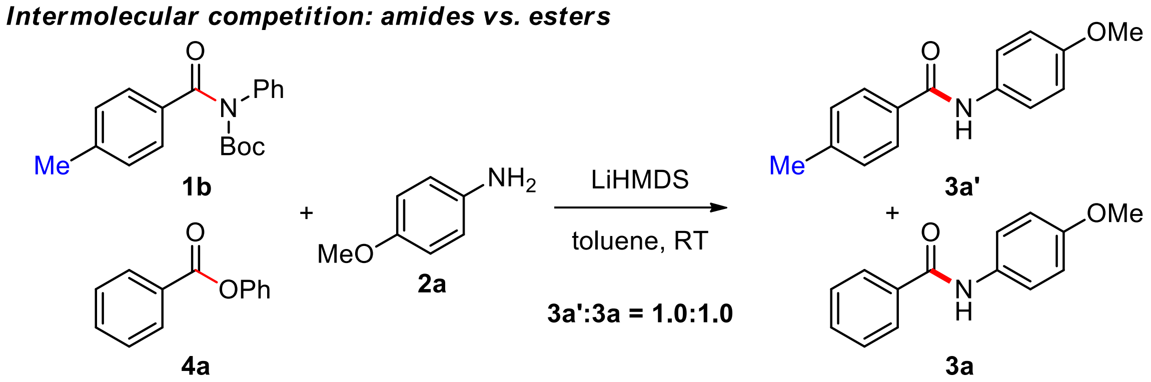

Supplementary Figure 3. Intermolecular Competition Experiments: Amides vs. Esters. *Conditions:* ***1*** *and* ***4*** *(1.0 equiv each), aniline (1.0 equiv), LiHMDS (3.0 equiv), toluene (0.25 M), 23 °C, 15 h.*

(4) Finally, intramolecular competition experiments revealed that anilines are inherently more reactive than aliphatic amines for both amide and ester substrates (Supplementary Figure 4).

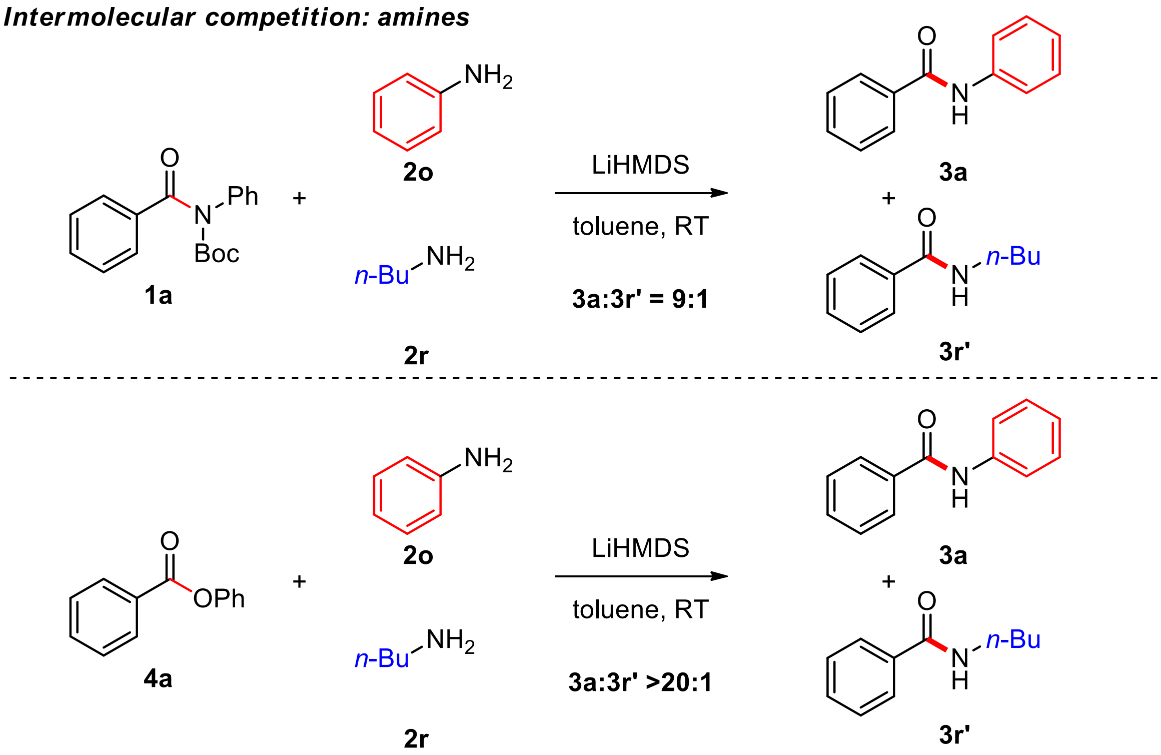

Supplementary Figure 4. Intermolecular Competition Experiments: Amines. *Conditions:* ***1*** *or* ***4*** *(1.0 equiv), amine (1.0 equiv each), LiHMDS (3.0 equiv), toluene (0.25 M), 23 °C, 15 h.*

Overall, these preliminary studies are consistent with the key role for amine deprotonation (cf. direct nucleophilic addition) and suggest a common manifold for transition-metal-free functionalization of amides and esters. Further studies to elucidate the mechanism of this and related transformations are ongoing and these results will be reported shortly.

Selected Optimization Studies Referred to from the Main Manuscript

Supplementary Table 1. Selected Optimization Studies in Transamidation of Amide **1a**.[a]

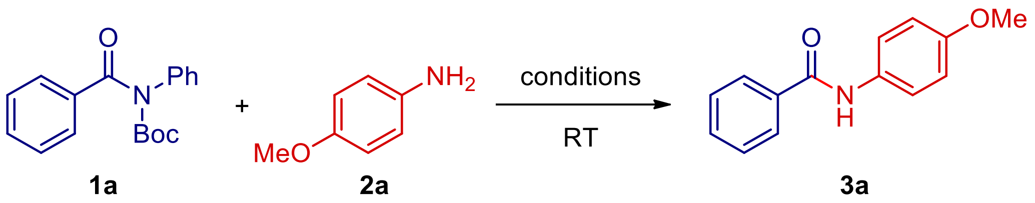

entry	base	solvent	temp (°C)	**2a** (equiv)	yield[b] (%)
1	KO*t*-Bu	THF	23	2.0	50
2	LiHMDS	toluene	23	2.0	>95
3	-	toluene	23	2.0	<5
4	LiHMDS	THF	23	2.0	61
5	NaHMDS	toluene	23	2.0	91
6	KHMDS	toluene	23	2.0	86
7	*n*-BuLi	toluene	23	2.0	62
8	LiHMDS	CH_3CN	23	2.0	91
9	LiHMDS	DMF	23	2.0	92
10	LiHMDS	Et_2O	23	2.0	87
11[c]	LiHMDS	toluene	23	1.0	36
12[d]	LiHMDS	toluene	23	1.5	83
13[e]	LiHMDS	toluene	23	1.5	87
14[e]	LiHMDS	toluene	23	2.0	90

[a]Conditions: amide (1.0 equiv), **2** (1.0-2.0 equiv), base (3.0 equiv), solvent (0.25 M), 23 °C, 15 h. [b]Determined by ^1H NMR and/or GC-MS. [c]LiHMDS (1.0 equiv). [d]LiHMDS (1.5 equiv). [e]LiHMDS (2.0 equiv).

Characterization Data of Amide and Ester Starting Materials

All amide and ester starting materials used in this study were prepared by procedures reported in the literature. **1a**,[1] **1b**,[1] **1c**,[1] **1d**,[1] **1e**,[1] **1f**,[1] **1g**,[1] **1h**,[1] **1i**,[2] **1j**,[3] **1k**,[1] **1l**,[4] **1m**,[3] **1n**,[3] **1o**,[3] **1p**,[5] **1q**,[6] **4a**,[7] **4b**,[8] **4c**,[9] **4d**,[1] **4e**,[1] **4f**,[9] **4g**,[1] **4h**,[10] **4i**,[11] **4j**,[12] **4k**,[13] **4l**,[14] **5a**,[15] **5b**,[16] **5c**,[17] **5d**,[16] **5e**,[17] **5f**,[16] **5g**[15] have been previously reported. Spectroscopic data match those reported in the literature.

Note: The availability of operationally-simple, straightforward and high-yielding synthetic methods for the direct site-selective N-Boc and N-Ts protection of the amide bond (primary and secondary) renders the transition-metal-catalyzed and transition-metal-free manifold of the amide bond highly valuable to practitioners of synthesis. In our hands, N-tert-butoxycarbonylation of the amide bond typically proceeds in >90% yields on gram scale.[18–20] Direct preparation of N-sulfonyl amides (Ts, Ms) from secondary amides[5,21] an N-acyl-pyrroles from primary amides is well-established.[6] N,N-Boc_2-benzamides are routinely prepared by site-selective double N-acylation of the amide bond.[22–25] Methods for the synthesis of aromatic esters are well-established, and have found broad application in transition-metal-catalyzed C–O activation by Ni, Pd and Rh catalysis.[26]

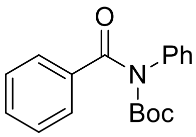

***tert*-Butyl benzoyl(phenyl)carbamate (1a).** White solid. ^{1}H NMR (500 MHz, $CDCl_3$) δ 7.76 (d, *J* = 7.1 Hz, 2 H), 7.55 (t, *J* = 7.4 Hz, 1 H), 7.49-7.43 (m, 4 H), 7.37 (t, *J* = 7.4 Hz, 1 H), 7.30 (d, *J* = 7.4 Hz, 2 H), 1.26 (s, 9 H). ^{13}C NMR (125 MHz, $CDCl_3$) δ 172.78, 153.30, 139.10, 136.98, 131.72, 129.21, 128.28, 128.14, 127.96, 127.80, 83.50, 27.49.

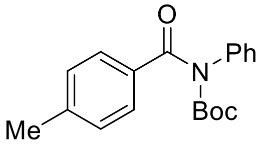

***tert*-Butyl (4-methylbenzoyl)(phenyl)carbamate (1b).** White solid. ^{1}H NMR (500 MHz, CDCl3) δ 7.67 (d, *J* = 7.9 Hz, 2 H), 7.44 (t, *J* = 7.7 Hz, 2 H), 7.35 (t, *J* = 7.2 Hz, 1 H), 7.30-7.25 (m, 4 H), 2.44 (s, 3 H), 1.29 (s, 9 H). ^{13}C NMR (125 MHz, CDCl3) δ 172.70, 153.46, 142.50, 139.32, 133.91, 129.16, 128.94, 128.50, 127.89, 127.64, 83.27, 27.56, 21.64.

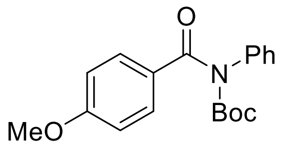

***tert*-Butyl (4-methoxybenzoyl)(phenyl)carbamate (1c).** Oil. ^{1}H NMR (500 MHz, $CDCl_3$) δ 7.77 (d, *J* = 7.6 Hz, 2 H), 7.43 (t, *J* = 7.2 Hz, 2 H), 7.33 (t, *J* = 7.3 Hz, 1 H), 7.28 (d, *J* = 7.9 Hz, 2 H), 6.95 (d, *J* = 7.7 Hz, 2 H), 3.88 (s, 3 H), 1.32 (s, 9 H). ^{13}C NMR (125 MHz, $CDCl_3$) δ 172.07, 162.78, 153.53, 139.48, 130.86, 129.12, 128.72, 127.75, 127.48, 113.56, 83.10, 55.49, 27.65.

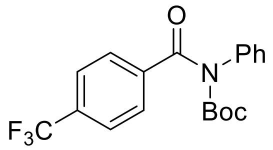

***tert*-Butyl phenyl(4-(trifluoromethyl)benzoyl)carbamate (1d).** White solid. ^{1}H NMR (500 MHz, $CDCl_3$) δ 7.84 (d, *J* = 8.0 Hz, 2 H), 7.74 (d, *J* = 8.2 Hz, 2 H), 7.47 (t, *J* = 7.6 Hz, 2 H), 7.40 (t, *J* = 7.4 Hz, 1 H), 7.29 (d, *J* = 7.2 Hz, 2 H), 1.27 (s, 9 H). ^{13}C NMR (125 MHz, $CDCl_3$) δ 171.38, 152.87, 140.33, 138.52, 133.07 (J^F = 65.0 Hz), 129.31, 128.16, 127.98, 125.31 (J^F = 7.5 Hz), 123.64 (J^F = 270.0 Hz), 84.12, 27.49. ^{19}F NMR (471 MHz, $CDCl_3$) δ -62.93.

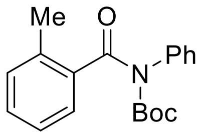

***tert*-Butyl (2-methylbenzoyl)(phenyl)carbamate (1e).** White solid. ^{1}H NMR (500 MHz, $CDCl_3$) δ 7.48 (q, *J* = 7.2 Hz, 3 H), 7.40 (d, *J* = 6.9 Hz, 1 H), 7.38-7.34 (m, 1 H), 7.32 (d, *J* = 7.9 Hz, 2 H), 7.27 (d, *J* = 7.4 Hz, 2 H), 2.52 (s, 3 H), 1.19 (s, 9 H). ^{13}C NMR (125 MHz, $CDCl_3$) δ 172.58, 152.85, 138.55, 137.86, 135.69, 130.73, 129.92, 129.22, 128.07, 127.98, 126.39, 125.53, 83.57, 27.34, 19.52.

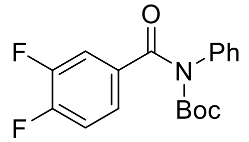

***tert*-Butyl phenyl(3,4-difluorobenzoyl)carbamate (1f).** White solid. ^{1}H NMR (500 MHz, $CDCl_3$) δ 7.61-7.56 (t, *J* = 7.3 Hz, 1 H), 7.52 (brs, 1 H), 7.45 (t, *J* = 7.4 Hz, 2 H), 7.38 (t, *J* = 7.2 Hz, 1 H), 7.25 (d, *J* = 7.9 Hz, 3 H), 1.33 (s, 9 H). ^{13}C NMR (125 MHz $CDCl_3$) δ 170.31, 152.56 (J^1 = 253.8 Hz), 153.00, 150.07 (J^F = 250.0 Hz), 138.70, 133.51 (J^F = 5.0 Hz), 129.28, 128.05, 127.84, 124.92 (J^F = 15.0, 7.5 Hz), 117.81 (J^F = 18.8 Hz), 117.26 (J^F = 17.5 Hz), 83.98, 27.60. ^{19}F NMR (471 MHz, $CDCl_3$) δ -131.63, -136.54.

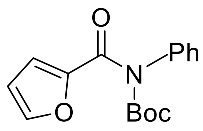

***tert*-Butyl (furan-2-carbonyl)(phenyl)carbamate (1g).** White solid. ^{1}H NMR (500 MHz, $CDCl_3$) δ 7.57-7.54 (m, 1 H), 7.43 (t, *J* = 7.6 Hz, 2 H), 7.35 (t, *J* = 7.4 Hz, 1 H), 7.28 (d, *J* = 7.2 Hz, 2 H), 7.04 (d, *J* = 3.5 Hz, 1 H), 6.53 (dd, *J* =

3.5, 1.7 Hz, 1 H), 1.42 (s, 9 H). ^{13}C NMR (125 MHz, $CDCl_3$) δ 161.23, 152.84, 148.49, 145.05, 138.64, 129.16, 127.96, 127.88, 118.13, 112.29, 83.34, 27.70.

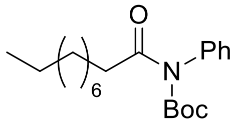

***tert*-Butyl (1-decanoyl)(phenyl)carbamate (1h).** White solid. ^{1}H NMR (500 MHz, $CDCl_3$) δ 7.41 (t, *J* = 7.2 Hz, 2 H), 7.34 (t, *J* = 7.3 Hz, 1 H), 7.09 (d, *J* = 7.7 Hz, 2 H), 2.92 (t, *J* = 7.4 Hz, 2 H), 1.70 (p, *J* = 7.3, 6.8 Hz, 2 H), 1.40 (s, 9 H), 1.29 (s, 12 H), 0.90 (t, *J* = 6.4 Hz, 3 H). ^{13}C NMR (125 MHz, $CDCl_3$) δ 175.97, 152.75, 139.17, 128.90, 128.22, 127.66, 82.93, 38.03, 31.90, 29.48, 29.30, 29.23, 27.84, 25.01, 22.69, 14.13.

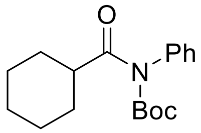

***tert*-Butyl (cyclohexanecarbonyl)(phenyl)carbamate (1i).** White solid. ^{1}H NMR (500 MHz, $CDCl_3$) δ 7.40 (t, *J* = 7.5 Hz, 2 H), 7.33 (t, *J* = 7.4 Hz, 1 H), 7.08 (d, *J* = 7.1 Hz, 2 H), 3.38 (tt, *J* = 11.4, 3.3 Hz, 1 H), 1.99 (d, *J* = 11.9 Hz, 2 H), 1.86-1.79 (m, 2 H), 1.74-1.67 (m, 1 H), 1.58-1.48 (m, 2 H), 1.43 (d, *J* = 7.9 Hz, 9 H), 1.40-1.22 (m, 3 H). ^{13}C NMR (125 MHz, $CDCl_3$) δ 179.41, 152.78, 139.49, 128.90, 128.17, 127.56, 82.94, 44.57, 29.65, 27.86, 25.88, 25.71.

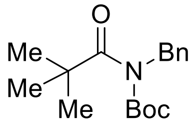

***tert*-Butyl benzyl(pivaloyl)carbamate (1j).** Oil. ^{1}H NMR (500 MHz, $CDCl_3$) δ 7.35-7.25 (m, 5 H), 4.74 (s, 2 H), 1.41 (s, 9 H), 1.29 (s, 9 H). ^{13}C NMR (125 MHz, $CDCl_3$) δ 185.69, 153.87, 138.33, 128.31, 127.93, 127.21, 82.58, 50.60, 43.35, 28.08, 27.88.

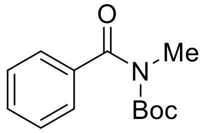

***tert*-Butyl benzoyl(methyl)carbamate (1k).** Oil. ^{1}H NMR (500 MHz, $CDCl_3$) δ 7.53 (d, *J* = 7.2 Hz, 2 H), 7.48 (t, *J* = 7.4 Hz, 1 H), 7.40 (t, *J* = 7.5 Hz, 2 H), 3.33 (s, 3 H), 1.17 (s, 9 H). ^{13}C NMR (125 MHz, $CDCl_3$) δ 173.59, 153.54, 137.91, 130.85, 127.99, 127.41, 82.98, 32.55, 27.37**.**

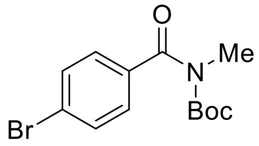

***tert*-Butyl (4-bromobenzoyl)(methyl)carbamate (1l).** White solid. ^{1}H NMR (500 MHz, $CDCl_3$) δ 7.56 (d, *J* = 8.2 Hz, 2 H), 7.41 (d, *J* = 8.1 Hz, 2 H), 3.31 (s, 3 H), 1.24 (s, 9 H). ^{13}C NMR (125 MHz, $CDCl_3$) δ 172.55,

153.27, 136.65, 131.22, 129.04, 125.32, 83.41, 32.60, 27.50.

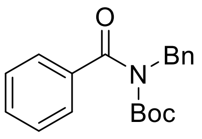

***tert*-Butyl benzoyl(benzyl)carbamate (1m).** Oil. ^{1}H NMR (500 MHz, $CDCl_3$) δ 7.54 (d, *J* = 7.0 Hz, 2 H), 7.47 (dd, *J* = 10.9, 7.4 Hz, 3 H), 7.41 (d, *J* = 7.7 Hz, 2 H), 7.37 (s, 2 H), 7.30 (s, 1 H), 5.02 (s, 2 H), 1.15 (s, 9 H). ^{13}C NMR (125 MHz, $CDCl_3$) δ 173.09, 153.46, 137.86, 137.73, 131.04, 128.45, 128.16, 128.06, 127.46, 127.40, 83.18, 48.88, 27.35.

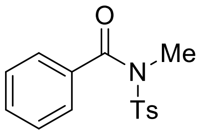

***N*-Methyl-*N*-tosylbenzamide (1n).** Oil. ^{1}H NMR (500 MHz, $CDCl_3$) δ 7.85 (d, *J* = 7.4 Hz, 2 H), 7.57 (d, *J* = 7.3 Hz, 2 H), 7.53 (t, *J* = 7.3 Hz, 1 H), 7.43 (t, *J* = 7.3 Hz, 2 H), 7.35 (d, *J* = 7.6 Hz, 2 H), 3.30 (s, 3 H), 2.47 (s, 3 H). ^{13}C NMR (125 MHz, $CDCl_3$) δ 171.48, 144.92, 135.21, 134.52, 131.96, 129.63, 128.48, 128.42, 128.30, 35.61, 21.68.

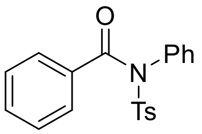

***N*-Phenyl-*N*-tosylbenzamide (1o).** White solid. ^{1}H NMR (500 MHz, $CDCl_3$) δ 7.86 (d, *J* = 8.3 Hz, 2 H), 7.46 (d, *J* = 8.3 Hz, 2 H), 7.34 (d, *J* = 8.1 Hz, 2 H), 7.30 (d, *J* = 8.7 Hz, 4 H), 7.19 (t, *J* = 7.1 Hz, 4 H), 2.48 (s, 3 H). ^{13}C NMR (125 MHz, $CDCl_3$) δ 169.90, 144.81, 137.43, 135.25, 133.67, 131.74, 130.40, 129.49, 129.25, 129.10, 129.03, 128.60, 127.98, 21.73.

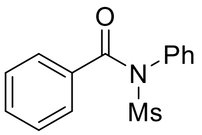

***N*-(Methylsulfonyl)-*N*-phenylbenzamide (1p).** White solid. ^{1}H NMR (500 MHz, $CDCl_3$) δ 7.57 (d, *J* = 7.2 Hz, 2 H), 7.38-7.28 (m, 6 H), 7.25 (t, *J* = 7.9 Hz, 2 H), 3.46 (s, 3 H). ^{13}C NMR (125 MHz, $CDCl_3$) δ 171.10, 136.96, 133.14, 132.24, 129.82, 129.68, 129.47, 129.26, 128.21, 40.39.

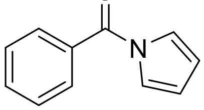

Phenyl(1*H*-pyrrol-1-yl)methanone (1q). Oil. ^{1}H NMR (500 MHz, $CDCl_3$) δ 7.77 (d, *J* = 7.4 Hz, 2 H), 7.63 (t, *J* = 7.4 Hz, 1 H), 7.53 (t, *J* = 7.4 Hz, 2 H), 7.32 (s, 2 H), 6.38 (s, 2 H). ^{13}C NMR (125 MHz, $CDCl_3$) δ 167.71, 133.27, 132.28, 129.50, 128.49, 121.31, 113.16.

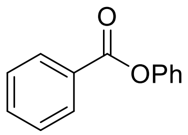

Phenyl benzoate (4a). White solid. ^{1}H NMR (500 MHz, $CDCl_3$) δ 8.29-8.20 (m, 2 H), 7.67 (t, *J* = 7.4 Hz, 1 H), 7.55 (t, *J* = 7.7 Hz, 2 H), 7.47 (t, *J* = 7.9 Hz, 2 H), 7.31 (t, *J* = 7.4 Hz, 1 H), 7.25 (d, *J* = 7.6 Hz, 2 H). ^{13}C NMR (125 MHz, $CDCl_3$) δ 165.21, 150.99, 133.60, 130.20, 129.62, 129.52, 128.59, 125.91, 121.74.

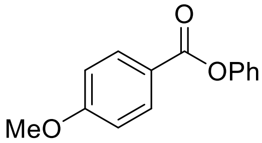

Phenyl 4-methoxybenzoate (4b). White solid. ^{1}H NMR (500 MHz, $CDCl_3$) δ 8.19 (d, *J* = 8.9 Hz, 2 H), 7.45 (t, *J* = 7.9 Hz, 2 H), 7.31-7.27 (m, 1 H), 7.24 (d, *J* = 7.9 Hz, 2 H), 7.02 (d, *J* = 8.8 Hz, 2 H), 3.92 (s, 3 H). ^{13}C NMR (125 MHz, $CDCl_3$) δ 164.92, 163.91, 151.11, 132.31, 129.45, 125.73, 121.93, 121.82, 113.86, 55.53.

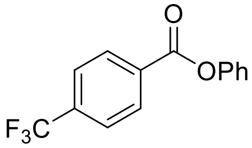

Phenyl 4-(trifluoromethyl)benzoate (4c). White solid. ^{1}H NMR (500 MHz, $CDCl_3$) δ 8.35 (d, *J* = 8.1 Hz, 2 H), 7.81 (d, *J* = 8.2 Hz, 2 H), 7.48 (t, *J* = 7.9 Hz, 2 H), 7.33 (t, *J* = 7.5 Hz, 1 H), 7.27-7.23 (m, 2 H). ^{13}C NMR (125 MHz, $CDCl_3$) δ 164.01, 150.68, 135.05 (J^F = 32.8 Hz), 132.86, 130.59, 129.63, 126.25, 125.64 (J^F = 3.7 Hz), 123.58 (J^F = 272.8 Hz), 121.55. ^{19}F NMR (471 MHz, $CDCl_3$) δ -63.15.

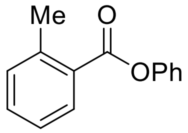

Phenyl 2-methylbenzoate (4d). Oil. ^{1}H NMR (500 MHz, $CDCl_3$) δ 8.20 (d, *J* = 7.7 Hz, 1 H), 7.51 (t, *J* = 7.5 Hz, 1 H), 7.47 (t, *J* = 7.3 Hz, 2 H), 7.36 (t, *J* = 8.0 Hz, 2 H), 7.32-7.28 (m, 1 H), 7.25 (d, *J* = 7.9 Hz, 2 H), 2.71 (s, 3 H). ^{13}C NMR (125 MHz, $CDCl_3$) δ 165.86, 150.96, 141.34, 132.73, 131.98, 131.18, 129.51, 128.62, 125.94, 125.84, 121.85, 21.96.

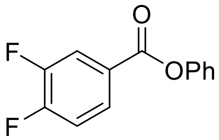

Phenyl 3,4-difluorobenzoate (4e). White solid. ^{1}H NMR (500 MHz, $CDCl_3$) δ 8.10-7.96 (m, 2 H), 7.47 (t, *J* = 7.3 Hz, 2 H), 7.38-7.29 (m, 2 H), 7.23 (d, *J* = 7.9 Hz, 2 H). ^{13}C NMR (125 MHz, $CDCl_3$) δ 163.28, 154.06 (J^F = 257.2

Hz), 150.67, 150.24 (J^F = 250.9 Hz), 129.60, 127.24 (dd, J^F = 254.5, 13.6 Hz), 126.63 (J^F = 7.7 Hz), 126.20, 121.53, 119.54 (J^F = 18.8 Hz), 117.63 (J^F = 17.9 Hz). ^{19}F NMR (471 MHz, $CDCl_3$) δ -128.92, -136.00.

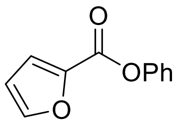

Phenyl furan-2-carboxylate (4f). Oil. ^{1}H NMR (500 MHz, $CDCl_3$) δ 7.70 (s, 1 H), 7.45 (t, J = 7.4 Hz, 2 H), 7.41 (d, J = 2.3 Hz, 1 H), 7.32-7.28 (m, 1 H), 7.24 (d, J = 7.9 Hz, 2 H), 6.62 (d, J = 1.4 Hz, 1 H). ^{13}C NMR (125 MHz, $CDCl_3$) δ 156.94, 150.23, 147.14, 144.06, 129.53, 126.08, 121.62, 119.43, 112.19.

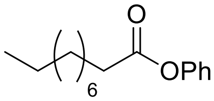

Phenyl decanoate (4g). Oil. ^{1}H NMR (500 MHz, $CDCl_3$) δ 7.40 (t, J = 7.8 Hz, 2 H), 7.25 (t, J = 7.4 Hz, 1 H), 7.10 (d, J = 7.7 Hz, 2 H), 2.58 (t, J = 7.5 Hz, 2 H), 1.85-1.73 (m, 2 H), 1.50-1.41 (m, 2 H), 1.40-1.25 (m, 10 H), 0.92 (t, J = 6.7 Hz, 3 H). ^{13}C NMR (125 MHz, $CDCl_3$) δ 172.4, 150.8, 129.4, 125.7, 121.6, 34.4, 31.9, 29.5, 29.3, 29.1, 25.0, 22.7, 14.1.

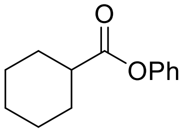

Phenyl cyclohexanecarboxylate (4h). Oil. ^{1}H NMR (500 MHz, $CDCl_3$) δ 7.40 (t, J = 7.8 Hz, 2 H), 7.25 (t, J = 7.3 Hz, 1 H), 7.10 (d, J = 8.3 Hz, 2 H), 2.60 (tt, J = 11.2, 3.6 Hz, 1 H), 2.12-2.09 (m, 2 H), 1.90-1.82 (m, 2 H), 1.74-1.71 (m, 1 H), 1.68-1.58 (m, 2 H), 1.45-1.26 (m, 3 H). ^{13}C NMR (125 MHz, $CDCl_3$) δ 174.61, 150.93, 129.38, 125.64, 121.59, 43.23, 28.99, 25.76, 25.40.

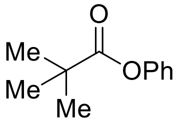

Phenyl pivalate (4i). Oil. ^{1}H NMR (500 MHz, $CDCl_3$) ^{1}H NMR (500 MHz, $CDCl_3$) δ 7.40 (t, J = 7.8 Hz, 2 H), 7.25 (t, J = 7.2 Hz, 1 H), 7.09 (d, J = 8.1 Hz, 2 H), 1.40 (s, 9 H). ^{13}C NMR (125 MHz, $CDCl_3$) δ 177.09, 151.15, 129.36, 125.59, 121.51, 39.08, 27.16.

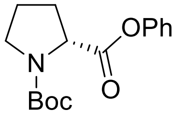

1-*tert*-Butyl 2-phenylpyrrolidine-1,2-dicarboxylate (4j). White solid. ^{1}H NMR (500 MHz, $CDCl_3$) δ 7.45-7.35 (m, 2 H), 7.24 (dd, J = 14.7, 7.4 Hz, 1 H), 7.12 (dd, J = 14.4, 8.0 Hz, 2 H), 4.48 (dd, J = 8.6, 4.2 Hz, 1 H), 3.70-3.43 (m, 2 H), 2.38 (ddd, J = 17.9, 12.9, 7.7 Hz, 1 H), 2.26-2.15 (m, 1 H), 2.13-2.03 (m, 1 H), 1.97 (td, J = 12.4,

5.5 Hz, 1 H), 1.49 (s, 9 H). ^{13}C NMR (125 MHz, $CDCl_3$) δ 171.62, 153.78, 150.61, 129.51, 125.94, 121.15, 80.23, 59.21, 46.46, 31.07, 30.03, 28.44.

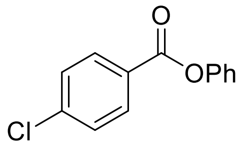

Phenyl 4-chlorobenzoate (4k). White solid. ^{1}H NMR (500 MHz, $CDCl_3$) δ 8.14-8.08 (m, 2 H), 7.47-7.38 (m, 4 H), 7.31-7.29 (m, 1 H), 7.28-7.18 (m, 2 H). ^{13}C NMR (125 MHz, $CDCl_3$) δ 164.61, 150.94, 140.41, 131.80, 129.81, 129.10, 128.31, 126.28, 121.87.

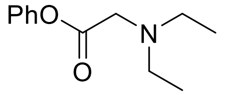

Phenyl diethylglycinate (4l). Oil. ^{1}H NMR (500 MHz, $CDCl_3$) δ 7.40 (t, *J* = 7.8 Hz, 2 H), 7.25 (t, *J* = 7.3 Hz, 1 H), 7.12 (d, *J* = 8.3 Hz, 2 H), 3.62 (s, 2 H), 2.78 (q, *J* = 7.2 Hz, 4 H), 1.15 (t, *J* = 7.2 Hz, 6 H). ^{13}C NMR (125 MHz, $CDCl_3$) δ 170.04, 150.51, 129.42, 125.82, 121.55, 54.19, 47.83, 12.43.

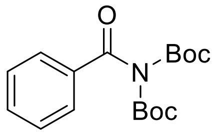

***N,N*-Boc$_2$-Benzamide (5a).** White solid. ^{1}HNMR (500 MHz, $CDCl_3$) δ 7.85 (d, *J* = 7.7 Hz, 2H), 7.61 (t, *J* = 7.4 Hz, 1 H), 7.49 (t, *J* = 7.3 Hz, 2 H), 1.39 (s, 18 H). ^{13}C NMR (125 MHz, $CDCl_3$) δ 149.77, 133.41, 129.09, 128.67, 84.27, 27.59.

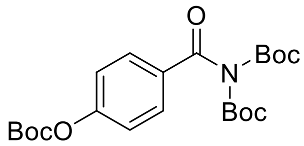

***N,N*-Boc$_2$-4-(*tert*-Butoxycarbonylhydroxy)benzamide (5b).** Oil. ^{1}H NMR (500 MHz, $CDCl_3$) δ 7.87 (d, *J* = 8.4 Hz, 2 H), 7.31 (d, *J* = 8.5 Hz, 2 H), 1.58 (s, 9 H), 1.39 (s, 18 H). ^{13}C NMR (125 MHz, $CDCl_3$) δ 168.26, 150.75, 149.61, 131.40, 130.71, 121.42, 84.38, 84.29, 27.65, 27.61.

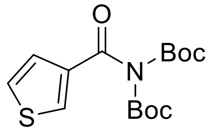

***N,N*-Boc$_2$-Thiophene-3-carboxamide (5c).** White solid. ^{1}H NMR (500 MHz, $CDCl_3$) δ 8.03 (s, 1 H), 7.44 (d, *J* = 5.0 Hz, 1 H), 7.35 (dd, *J* = 4.7, 2.8 Hz, 1 H), 1.40 (s, 18 H). ^{13}C NMR (125 MHz, $CDCl_3$) δ 163.34, 149.75, 137.69, 133.51, 127.76, 126.86, 84.37, 27.79.

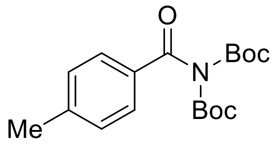

***N,N*-Boc2-4-Methylbenzamide (5d).** White solid. ^{1}H NMR (500 MHz, $CDCl_3$) δ 7.75 (d, *J* = 8.1 Hz, 2 H), 7.28 (d, *J* = 8.2 Hz, 2 H), 2.44 (s, 3

H), 1.39 (s, 18 H). ^{13}C NMR (125 MHz, $CDCl_3$) δ 169.02, 149.81, 144.53, 129.41, 129.35, 84.05, 27.62, 21.73.

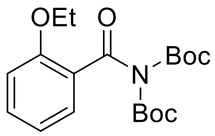

***N,N*-Boc$_2$-2-Ethoxybenzamide (5e).** White solid. ^{1}H NMR (500 MHz, $CDCl_3$) δ 7.61 (d, *J* = 6.3 Hz, 1 H), 7.43 (t, *J* = 7.0 Hz, 1 H), 6.99 (t, *J* = 7.5 Hz, 1 H), 6.87 (d, *J* = 8.3 Hz, 1 H), 4.03 (q, *J* = 6.7 Hz, 2 H), 1.43 (t, *J* = 6.9 Hz, 3 H), 1.39 (s, 18 H). ^{13}C NMR (125 MHz, $CDCl_3$) δ 168.02, 156.92, 149.91, 133.48, 130.98, 124.67, 120.69, 112.04, 83.84, 64.40, 27.64, 14.71.

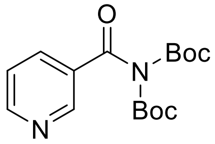

***N,N*-Boc$_2$-Nicotinamide (5f).** White solid. ^{1}H NMR (500 MHz, $CDCl_3$) δ 8.97 (s, 1 H), 8.78 (d, *J* = 4.8 Hz, 1 H), 8.07 (d, *J* = 7.9 Hz, 1 H), 7.42 (dd, *J* = 7.8, 4.9 Hz, 1 H), 1.41 (s, 18 H). ^{13}C NMR (125 MHz, $CDCl_3$) δ 167.95, 153.47, 149.79, 149.49, 136.23, 130.17, 123.42, 84.97, 27.56.

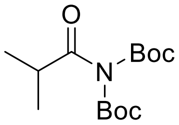

***N,N*-Boc$_2$-Isobutyramide (5g).** White solid. ^{1}H NMR (500 MHz, $CDCl_3$) δ 3.45 (dt, *J* = 13.6, 6.8 Hz, 1 H), 1.53 (s, 18 H), 1.20 (d, *J* = 6.9 Hz, 6 H). ^{13}C NMR (125 MHz, $CDCl_3$) δ 178.74, 149.68, 84.48, 34.42, 27.62, 18.90.

Characterization Data of Transamidation Products

All products reported in the manuscript are known compounds. **3a**,[27] **3b**,[27] **3c**,[27] **3d**,[28] **3e**,[27] **3f**,[27] **3g**,[27] **3h**,[29] **3i**,[30] **3j**,[31] **3k**,[28] **3l**,[32] **3m**,[27] **3n**,[30] **3o**,[27] **3p**,[27] **3q**,[27] **3r**,[27] **3s**,[27] **3t**,[27] **3u**,[27] **3v**,[33] **3w**,[34] **3x**,[35] **3y**,[28] **3z**,[36] **3aa**,[30] **3ab**,[28] **3ac**,[37] **3ad**,[28] **3ae**,[38] **3af**,[39] **3ag**,[40] **3ah**,[41] **3ai**,[27] **3aj**,[42] **3ak**,[43] **3al**,[44] **3am**,[28] **3an**,[45] **3ao**,[46] **3ap**,[47] **3aq**[48] have been previously reported. Spectroscopic data match those reported in the literature.

N-(4-Methoxyphenyl)benzamide (3a, Figure 2)

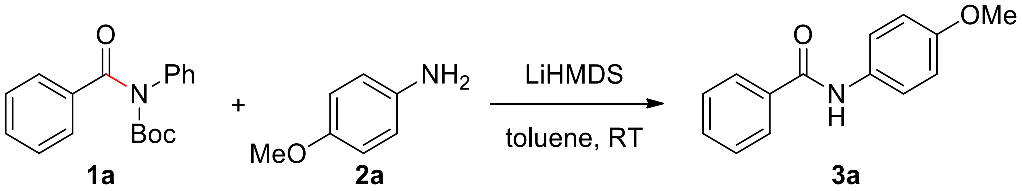

According to the general procedure, the reaction of *tert*-butyl benzoyl(phenyl)carbamate (3.36 mmol), *p*-anisidine (2.0 equiv) and LiHMDS (1.0 M in THF, 3.0 equiv) in toluene (0.25 M) for 15 h at room temperature, afforded after work-up and chromatography the title compound in 94% yield (0.720 g). White solid. ^{1}H NMR (500 MHz, $CDCl_3$) δ 7.88 (d, *J* = 7.6 Hz, 2 H), 7.82 (s, 1H), 7.57-7.55 (m, 3 H), 7.49 (t, *J* = 7.6 Hz, 2 H), 6.92 (d, *J* = 8.9 Hz, 2 H), 3.84 (s, 3 H). ^{13}C NMR (125 MHz, $CDCl_3$) δ 165.64, 156.65, 135.06, 131.71, 131.02, 128.76, 127.00, 122.13, 114.26, 55.53.

N-(4-(Trifluoromethyl)phenyl)benzamide (3b, Figure 2)

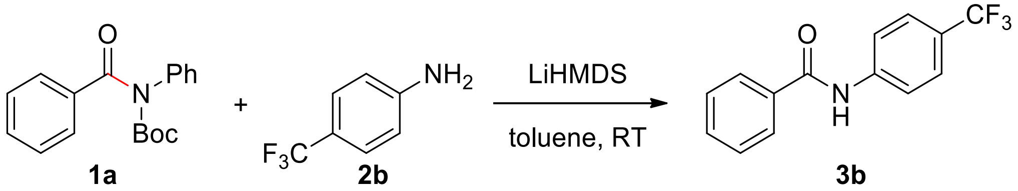

According to the general procedure, the reaction of *tert*-butyl benzoyl(phenyl)carbamate (0.10 mmol), 4-(trifluoromethyl)aniline (2.0 equiv) and LiHMDS (1.0 M in THF, 3.0 equiv) in toluene (0.25 M) for 15 h at room temperature, afforded after work-up and chromatography the title compound in 90% yield (23.9 mg). White solid. ^{1}H NMR (500 MHz, DMSO-d^6) δ 8.03 (d, *J* = 8.4 Hz, 2 H), 7.98 (d, *J* = 7.8 Hz, 2 H), 7.73 (d, *J* = 8.4 Hz, 2 H), 7.63 (t, *J* = 7.2 Hz, 1 H), 7.56

(t, *J* = 7.5 Hz, 2 H). ^{13}C NMR (125 MHz, DMSO-d^6) δ 166.55, 143.37, 135.00, 132.39, 128.94, 128.28, 126.39 (q, J^F = 3.8 Hz), 125.07 (d, J^F = 224.5 Hz), 123.86 (d, J^F = 14.9 Hz), 120.59. ^{19}F NMR (471 MHz, DMSO-d^6) δ -60.24.

Ethyl 4-benzamidobenzoate (3c, Figure 2)

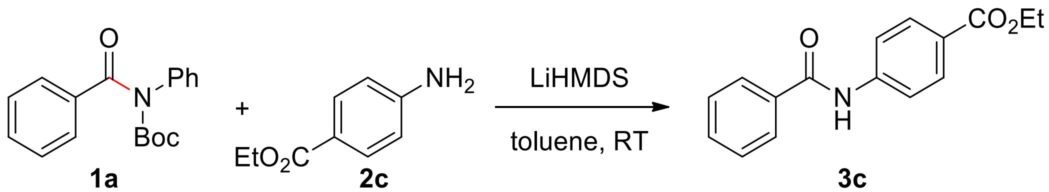

1a **2c** **3c**

According to the general procedure, the reaction of *tert*-butyl benzoyl(phenyl)carbamate (0.10 mmol), ethyl 4-aminobenzoate (1.5equiv) and LiHMDS (1.0 M in THF, 2.3 equiv) in toluene (0.25 M) for 15 h at room temperature, afforded after work-up and chromatography the title compound in 78% yield (20.0 mg). White solid. ^{1}H NMR (500 MHz, $CDCl_3$) δ 8.08 (d, *J* = 8.4 Hz, 3 H), 7.90 (d, *J* = 7.6 Hz, 2 H), 7.77 (d, *J* = 8.4 Hz, 2 H), 7.59 (t, *J* = 7.3 Hz, 1 H), 7.51 (t, *J* = 7.5 Hz, 2 H), 4.39 (q, *J* = 7.1 Hz, 2 H), 1.42 (t, *J* = 7.1 Hz, 3 H). ^{13}C NMR (125 MHz, $CDCl_3$) δ 166.14, 165.82, 142.07, 134.57, 132.23, 130.87, 128.92, 127.10, 126.23, 119.17, 60.93, 14.37.

***N*-(4-Hydroxyphenyl)benzamide (3d, Figure 2)**

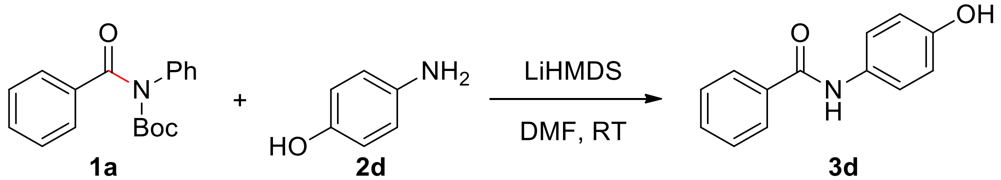

1a **2d** **3d**

According to the general procedure, the reaction of *tert*-butyl benzoyl(phenyl)carbamate (0.10 mmol, 4-aminophenol (2.0 equiv) and LiHMDS (1.0 M in THF, 4.0 equiv) in DMF (0.25 M) for 15 h at room temperature, afforded after work-up and chromatography the title compound in 62% yield (13.4 mg). White solid. ^{1}H NMR (500 MHz, DMSO-d^6) δ 10.02 (s, 1 H), 9.25 (s, 1 H), 7.93 (d, *J* = 7.4 Hz, 2 H), 7.62-7.46 (m, 5 H), 6.74 (d, *J* = 8.8 Hz, 2 H). ^{13}C NMR (125 MHz, DMSO-d^6) δ 165.40, 154.17, 135.64, 131.73, 131.16, 128.78, 127.96, 122.72, 115.42.

***N*-([1,1'-Biphenyl]-2-yl)benzamide (3e, Figure 2)**

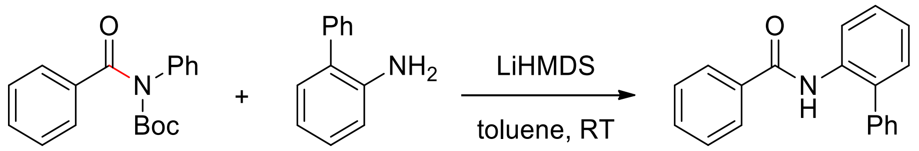

1a **2e** **3e**

According to the general procedure, the reaction of *tert*-butyl benzoyl(phenyl)carbamate (0.10 mmol), [1,1'-biphenyl]-2-amine (2.0 equiv) and LiHMDS (1.0 M in THF, 3.0 equiv) in toluene (0.25 M) for 15 h at room temperature, afforded after work-up and chromatography the title compound in 87% yield (23.8 mg). White solid. ^{1}H NMR (500 MHz, $CDCl_3$) δ 8.47 (d, J = 8.2 Hz, 1 H), 7.93 (s, 1 H), 7.53 (d, J = 7.9 Hz, 2 H), 7.46-7.42 (m, 2 H), 7.41-7.34 (m, 5 H), 7.31 (t, J = 7.6 Hz, 2 H), 7.23 (d, J = 7.4 Hz, 1 H), 7.15 (t, J = 7.5 Hz, 1 H). ^{13}C NMR (125 MHz, $CDCl_3$) 166.58, 138.68, 135.55, 135.41, 132.94, 132.34, 130.61, 129.99, 129.85, 129.37, 129.24, 128.81, 127.43, 124.97, 121.73.

N-(2,6-Dimethylphenyl)benzamide (3f, Figure 2)

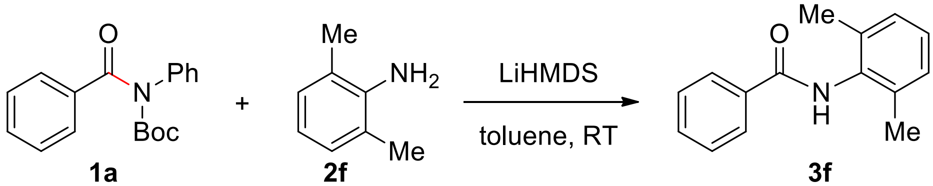

1a **2f** **3f**

According to the general procedure, the reaction of *tert*-butyl benzoyl(phenyl)carbamate (0.10 mmol), 2,6-dimethylaniline (2.0 equiv) and LiHMDS (1.0 M in THF, 3.0 equiv) in toluene (0.25 M) for 15 h at room temperature, afforded after work-up and chromatography the title compound in 92% yield (20.7 mg). White solid. ^{1}H NMR (500 MHz, $CDCl_3$) δ 7.95 (d, J = 7.6 Hz, 2 H), 7.59 (t, J = 7.3 Hz, 1 H), 7.52 (t, J = 7.6 Hz, 2 H), 7.45 (s, 1 H), 7.20-7.12 (m, 3 H), 2.31 (s, 6 H). ^{13}C NMR (125 MHz, $CDCl_3$) δ 165.88, 135.58, 134.55, 133.89, 131.82, 128.79, 128.32, 127.46, 127.23, 18.52.

N-(2,6-Diisopropylphenyl)benzamide (3g, Figure 2)

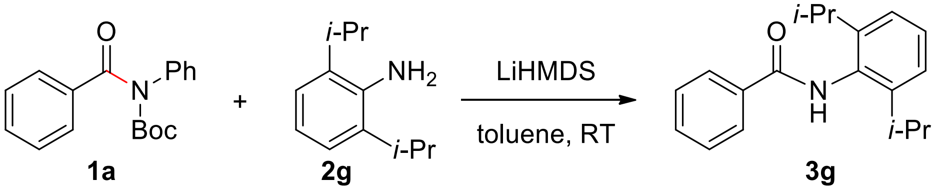

1a **2g** **3g**

According to the general procedure, the reaction of *tert*-butyl benzoyl(phenyl)carbamate (0.10 mmol), 2,6-diisopropylaniline (2.0 equiv) and LiHMDS (1.0 M in THF, 3.0equiv) in toluene (0.25 M) for 15 h at room temperature, afforded after work-up and chromatography the title compound in 98% yield (27.5 mg). White solid. ^{1}H NMR (500 MHz, $CDCl_3$) δ 7.95 (d, *J* = 7.7 Hz, 2 H), 7.60 (t, *J* = 7.4 Hz, 1 H), 7.53 (t, *J* = 7.6 Hz, 2 H), 7.42-7.34 (m, 2 H), 7.25 (d, *J* = 7.7 Hz, 2 H), 3.17 (dt, *J* = 13.7, 6.9 Hz, 2 H), 1.25 (d, *J* = 6.9 Hz, 12 H). ^{13}C NMR (125 MHz, $CDCl_3$) δ 166.95, 146.41, 134.66, 131.79, 131.17, 128.84, 128.52, 127.20, 123.59, 28.93, 23.68.

_N_-(3-Chlorophenyl)benzamide (3h, Figure 2)

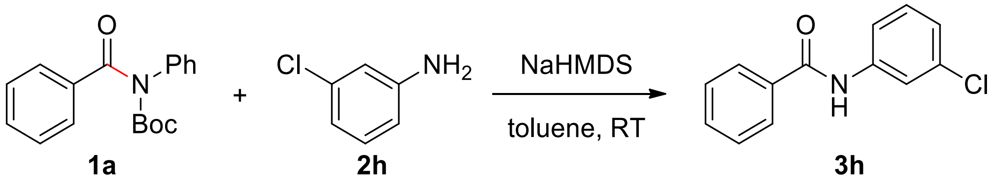

According to the general procedure, the reaction of *tert*-butyl benzoyl(phenyl)carbamate (0.10 mmol), 3-chloroaniline (2.0 equiv) and NaHMDS (1.0 M in THF, 3.0 equiv) in toluene (0.25 M) for 15 h at room temperature, afforded after work-up and chromatography the title compound in 90% yield (20.8 mg). White solid. ^{1}H NMR (500 MHz, $CDCl_3$) δ 7.99 (s, 1 H), 7.87 (d, *J* = 7.6 Hz, 2 H), 7.79 (s, 1 H), 7.57 (t, *J* = 7.3 Hz, 1 H), 7.50 (dd, *J* = 14.0, 7.0 Hz, 3 H), 7.33-7.25 (m, 1 H), 7.14 (d, *J* = 7.8 Hz, 1 H). ^{13}C NMR (125 MHz, $CDCl_3$) δ 165.85, 139.08, 134.75, 134.54, 132.12, 130.06, 128.87, 127.06, 124.63, 120.36, 118.21.

_N_-(9-Ethyl-9*H*-carbazol-3-yl)benzamide (3i, Figure 2)

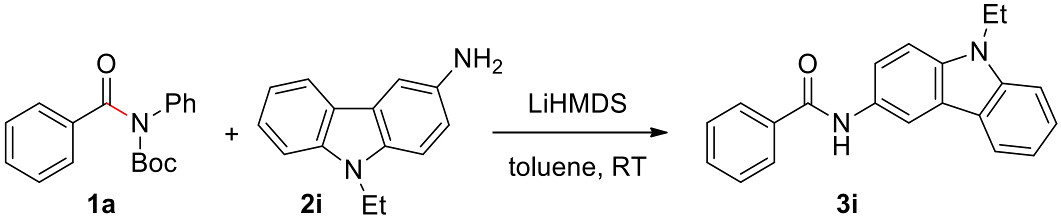

According to the general procedure, the reaction of *tert*-butyl benzoyl(phenyl)carbamate (0.10 mmol), 9-ethyl-9*H*-carbazol-3-amine (2.0 equiv) and LiHMDS (1.0 M in THF, 3.0 equiv) in toluene (0.25 M) for 15 h at room temperature, afforded after work-up and chromatography the title compound in 80% yield (25.1 mg). White solid. ^{1}H NMR (500 MHz, $CDCl_3$) δ 8.46 (s, 1 H),

8.10 (d, *J* = 7.7 Hz, 1 H), 8.05 (s, 1 H), 7.96 (d, *J* = 7.5 Hz, 2 H), 7.66 (d, *J* = 8.4 Hz, 1 H), 7.58 (t, *J* = 7.2 Hz, 1 H), 7.55-7.47 (m, 3 H), 7.43-7.38 (m, 2 H), 7.24 (t, *J* = 7.4 Hz, 1 H), 4.37 (q, *J* = 7.2 Hz, 2 H), 1.45 (t, *J* = 7.2 Hz, 3 H). ^{13}C NMR (125 MHz, $CDCl_3$) δ 165.79, 140.49, 137.41, 135.30, 131.63, 129.71, 128.77, 127.05, 125.92, 123.12, 122.80, 120.74, 119.75, 118.81, 113.20, 108.58, 37.64, 13.84.

N-(Perfluorophenyl)benzamide (3j, Figure 2)

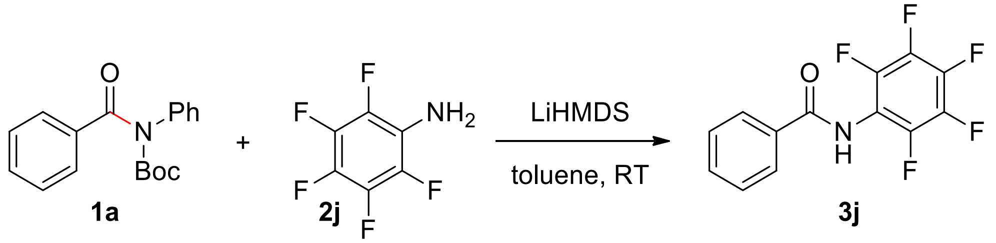

According to the general procedure, the reaction of *tert*-butyl benzoyl(phenyl)carbamate (0.10 mmol), 2,3,4,5,6-pentafluoroaniline (2.0 equiv) and LiHMDS (1.0 M in THF, 3.0 equiv) in toluene (0.25 M) for 15 h at room temperature, afforded after work-up and chromatography the title compound in 96% yield (27.6 mg). White solid. ^{1}H NMR (500 MHz, $CDCl_3$) δ 7.94 (d, *J* = 7.4 Hz, 2 H), 7.65 (t, *J* = 7.4 Hz, 1 H), 7.55 (t, *J* = 7.7 Hz, 2 H), 7.43 (s, 1 H). ^{13}C NMR (125 MHz, $CDCl_3$) δ 165.54, 144.05 (m), 142.06 (m), 138.90 (m), 136.87 (m), 132.92, 132.39, 128.99, 127.66. ^{19}F NMR (471 MHz, $CDCl_3$) δ -144.55 – -144.65 (m), -156.36 (t, *J* = 21.3 Hz), -162.04 – -162.12 (m).

N-(Pyridin-2-yl)benzamide (3k, Figure 2)

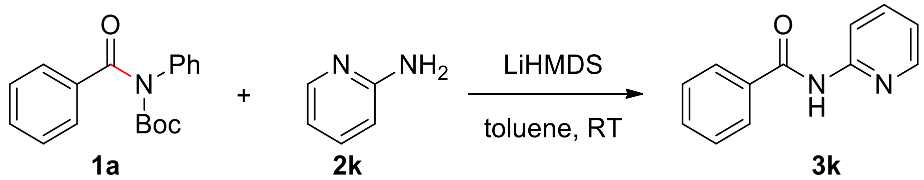

According to the general procedure, the reaction of *tert*-butyl benzoyl(phenyl)carbamate (0.10 mmol), pyridin-2-amine (2.0 equiv) and LiHMDS (1.0 M in THF, 3.0equiv) in toluene (0.25 M) for 15 h at room temperature, afforded after work-up and chromatography the title compound in 90% yield (17.8 mg). White solid. ^{1}H NMR (500 MHz, DMSO-d^6) δ 10.77 (s, 1 H), 8.40 (d, *J* = 3.9 Hz, 1 H), 8.20 (d, *J* = 8.3 Hz, 1 H), 8.04 (d, *J* = 7.5 Hz, 2 H), 7.89-7.83 (m, 1 H), 7.60 (t, *J* =

7.3 Hz, 1 H), 7.52 (t, *J* = 7.6 Hz, 2 H), 7.17 (dd, *J* = 7.2, 4.9 Hz, 1 H). ^{13}C NMR (125 MHz, DMSO-d^6) δ 166.45, 152.67, 148.41, 138.57, 134.56, 132.39, 128.82, 128.46, 120.28, 115.21.

N-(Quinolin-8-yl)benzamide (3l, Figure 2)

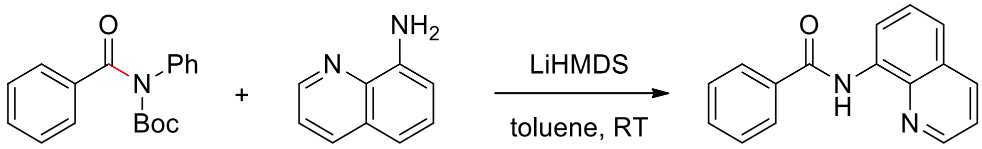

1a **2l** **3l**

According to the general procedure, the reaction of *tert*-butyl benzoyl(phenyl)carbamate (0.10 mmol), quinolin-8-amine (2.0 equiv) and LiHMDS (1.0 M in THF, 3.0 equiv) in toluene (0.25 M) for 15 h at room temperature, afforded after work-up and chromatography the title compound in 87% yield (21.6 mg). White solid. ^{1}H NMR (500 MHz, $CDCl_3$) δ 10.77 (s, 1 H), 8.97 (dd, *J* = 7.6, 1.1 Hz, 1 H), 8.87 (dd, *J* = 4.2, 1.6 Hz, 1 H), 8.20 (dd, *J* = 8.2, 1.5 Hz, 1 H), 8.12 (dd, *J* = 8.0, 1.4 Hz, 2 H), 7.64-7.55 (m, 5 H), 7.49 (dd, *J* = 8.2, 4.2 Hz, 1 H). ^{13}C NMR (125 MHz, $CDCl_3$) δ 165.49, 148.31, 138.81, 136.41, 135.19, 134.62, 131.86, 128.82, 128.02, 127.49, 127.32, 121.71, 116.57.

***N*-Methyl-*N*-phenylbenzamide (3m, Figure 2)**

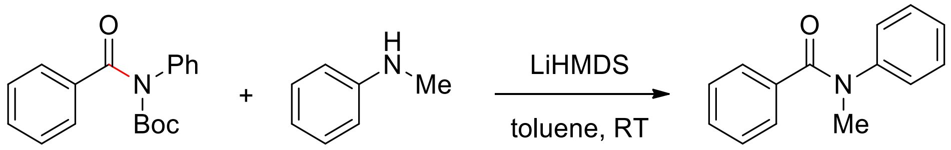

1a **2m** **3m**

According to the general procedure, the reaction of *tert*-butyl benzoyl(phenyl)carbamate (0.10 mmol), *N*-methylaniline (2.0 equiv) and LiHMDS (1.0 M in THF, 3.0 equiv) in toluene (0.25 M) for 15 h at room temperature, afforded after work-up and chromatography the title compound in 96% yield (20.2 mg). Colorless oil. ^{1}H NMR (500 MHz, $CDCl_3$) δ 7.32 (d, *J* = 7.5 Hz, 2 H), 7.26-7.21 (m, 3 H), 7.19-7.13 (m, 3 H), 7.05 (d, *J* = 7.8 Hz, 2 H), 3.52 (s, 3 H). ^{13}C NMR (125 MHz, $CDCl_3$) δ 170.71, 144.93, 135.93, 129.60, 129.15, 128.73, 127.73, 126.93, 126.50, 38.42.

Morpholino(phenyl)methanone (3n, Figure 2)

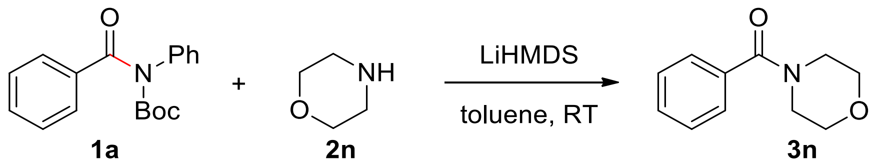

According to the general procedure, the reaction of *tert*-butyl benzoyl(phenyl)carbamate (0.10 mmol), morpholine (2.0 equiv) and LiHMDS (1.0 M in THF, 3.0 equiv) in toluene (0.25 M) for 15 h at room temperature, afforded after work-up and chromatography the title compound in 87% yield (16.6 mg). White solid. ^{1}H NMR (500 MHz, $CDCl_3$) δ 7.43-7.42 (m, 5 H), 3.90-3.38 (m, 8 H). ^{13}C NMR (125 MHz, $CDCl_3$) δ 170.45, 135.35, 129.89, 128.58, 127.10, 66.93.

Methyl 4-(phenylcarbamoyl)benzoate (3o, Figure 2)

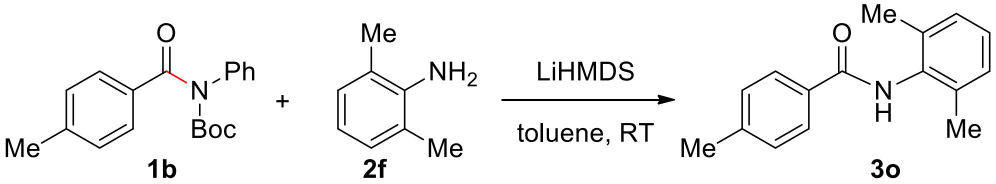

According to the general procedure, the reaction of *tert*-butyl (4-methylbenzoyl)(phenyl) carbamate (0.10 mmol), 2,6-dimethylaniline (2.0 equiv) and LiHMDS (1.0 M in THF, 3.0 equiv) in toluene (0.25 M) for 15 h at room temperature, afforded after work-up and chromatography the title compound in 83% yield (19.8 mg). White solid. ^{1}H NMR (500 MHz, $CDCl_3$) δ 7.85 (d, J = 7.9 Hz, 2 H), 7.36-7.28 (m, 3 H), 7.19-7.12 (m, 3 H), 2.47 (s, 3 H), 2.31 (s, 6 H). ^{13}C NMR (125 MHz, $CDCl_3$) δ 165.76, 142.33, 135.57, 133.97, 131.68, 129.44, 128.30, 127.37, 127.24, 21.52, 18.53.

***N*-(2,6-Dimethylphenyl)-4-methoxybenzamide (3p, Figure 2)**

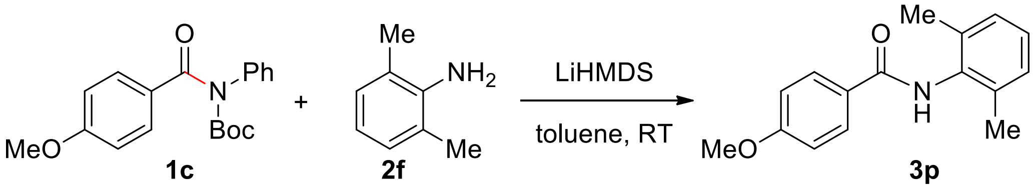

According to the general procedure, the reaction of *tert*-butyl (4-methoxybenzoyl)(phenyl) carbamate (0.10 mmol), 2,6-dimethylaniline (2.0equiv) and LiHMDS (1.0 M in THF, 3.0 equiv) in toluene (0.25 M) for 15 h at room temperature, afforded after work-up and chromatography

the title compound in 93% yield (21.1 mg). White solid. ^{1}H NMR (500 MHz, $CDCl_3$) δ 7.92 (d, *J* = 8.7 Hz, 2 H), 7.35 (s, 1 H), 7.19-7.11 (m, 3 H), 7.01 (d, *J* = 8.6 Hz, 2 H), 3.91 (s, 3 H), 2.30 (s, 6 H). ^{13}C NMR (125 MHz, $CDCl_3$) δ 165.36, 162.47, 135.58, 134.09, 129.09, 128.28, 127.31, 126.73, 113.96, 55.50, 18.53.

N-(2,6-Dimethylphenyl)-4-(trifluoromethyl)benzamide (3q, Figure 2)

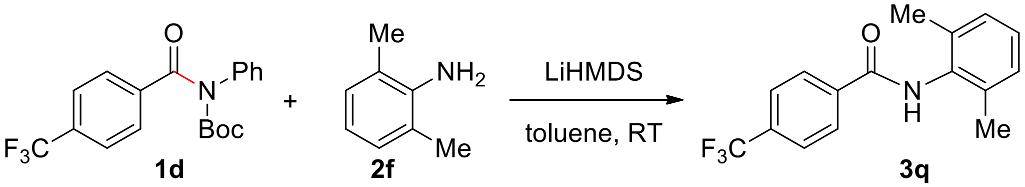

According to the general procedure, the reaction of *tert*-butyl phenyl(4-(trifluoromethyl)benzoyl) carbamate (0.10 mmol), 2,6-dimethylaniline (2.0 equiv) and LiHMDS (1.0 M in THF, 3.0 equiv) in toluene (0.25 M) for 15 h at room temperature, afforded after work-up and chromatography the title compound in 84% yield (24.6 mg). White solid. ^{1}H NMR (500 MHz, $CDCl_3$) δ 8.03 (d, *J* = 8.1 Hz, 2 H), 7.75 (d, *J* = 8.1 Hz, 2 H), 7.58 (s, 1 H), 7.21-7.14 (m, 3 H), 2.29 (s, 6 H). ^{13}C NMR (125 MHz, $CDCl_3$) δ 164.70, 137.71, 135.50, 133.45, 128.41, 127.76, 127.71, 125.82 (q, J^F = 3.7 Hz), 122.57, 18.45. ^{19}F NMR (471 MHz, $CDCl_3$) δ -62.91.

N-(2,6-Dimethylphenyl)-2-methylbenzamide (3r, Figure 2)

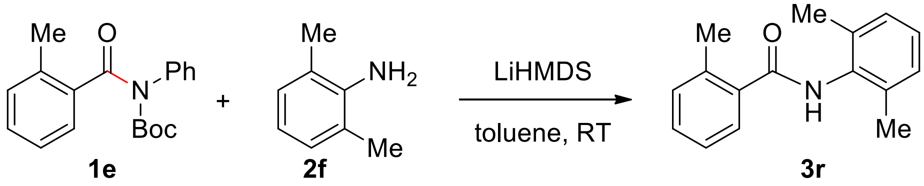

According to the general procedure, the reaction of *tert*-butyl (2-methylbenzoyl)(phenyl) carbamate (0.10 mmol), 2,6-dimethylaniline (2.0equiv) and LiHMDS (1.0 M in THF, 3.0 equiv) in toluene (0.25 M) for 15 h at room temperature, afforded after work-up and chromatography the title compound in 80% yield (19.1 mg). White solid. ^{1}H NMR (500 MHz, $CDCl_3$) δ 7.60 (d, *J* = 7.5 Hz, 1 H), 7.40 (t, *J* = 7.4 Hz, 1 H), 7.35-7.28 (m, 2 H), 7.20-7.14 (m, 3 H), 7.04 (s, 1 H), 2.58 (s, 3 H), 2.37 (s, 6 H). ^{13}C NMR (125 MHz, $CDCl_3$) δ 168.44, 136.65, 136.38, 135.56, 133.64, 131.31, 130.22, 128.40, 127.59, 126.68, 125.88, 19.97, 18.70.

N-(2,6-Dimethylphenyl)-3,4-difluorobenzamide (3s, Figure 2)

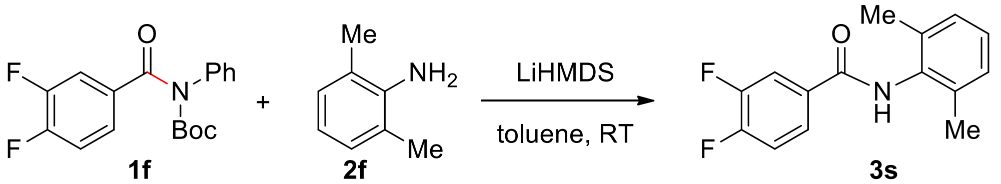

According to the general procedure, the reaction of *tert*-butyl (3,4-difluorobenzoyl)(phenyl) carbamate (0.10 mmol), 2,6-dimethylaniline (2.0equiv) and LiHMDS (1.0 M in THF, 3.0 equiv) in toluene (0.25 M) for 15 h at room temperature, afforded after work-up and chromatography the title compound in 92% yield (24.0 mg). White solid. ^{1}H NMR (500 MHz, $CDCl_3$) δ 7.79 (dd, J = 13.1, 4.9 Hz, 1 H), 7.68 (d, J = 8.2 Hz, 1 H), 7.39 (s, 1 H), 7.32-7.28 (m, 1 H), 7.17 (dt, J = 16.6, 6.5 Hz, 3 H), 2.28 (s, 6 H). ^{13}C NMR (125 MHz, $CDCl_3$) δ 163.75, 152.60 (dd, J^F = 290.5, 9.8 Hz), 135.45, 133.42, 132.51-130.72 (m), 128.41, 127.73, 123.57 (dd, J^F = 7.1, 3.7 Hz), 117.42 (dd, J^F= 66.4, 18.3 Hz), 18.44. ^{19}F NMR (471 MHz, $CDCl_3$) δ -131.99, -135.58.

N-(2,6-Dimethylphenyl)furan-2-carboxamide (3t, , Figure 2)

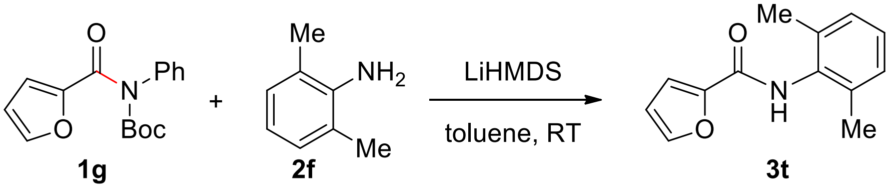

According to the general procedure, the reaction of *tert*-butyl (furan-2-carbonyl)(phenyl) carbamate (0.10 mmol), 2,6-dimethylaniline (2.0 equiv) and LiHMDS (1.0 M in THF, 3.0 equiv) in toluene (0.25 M) for 15 h at room temperature, afforded after work-up and chromatography the title compound in 94% yield (20.2 mg). White solid. ^{1}H NMR (500 MHz, $CDCl_3$) δ 7.63 (s, 1 H), 7.55 (s, 1 H), 7.25 (d, J = 3.4 Hz, 1 H), 7.19-7.14 (m, 3 H), 6.62-6.57 (m, 1 H), 2.31 (s, 6 H). ^{13}C NMR (125 MHz, $CDCl_3$) δ 156.45, 147.93, 144.16, 135.71, 132.91, 128.29, 127.56, 115.03, 112.42, 18.56.

N-(2,6-Dimethylphenyl)decanamide (3u, Figure 2)

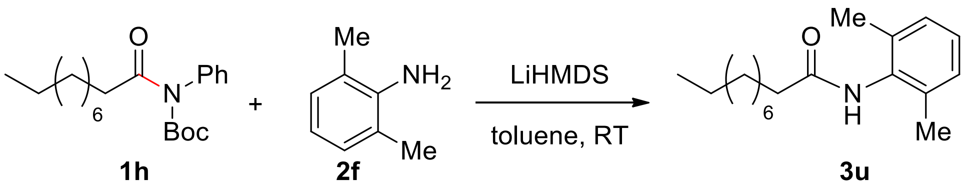

According to the general procedure, the reaction of *tert*-butyl decanoyl(phenyl)carbamate (0.10 mmol), 2,6-dimethylaniline (2.0equiv) and LiHMDS (1.0 M in THF, 3.0 equiv) in toluene (0.25 M) for 15 h at room temperature, afforded after work-up and chromatography the title compound in 81% yield (22.3 mg). White solid. ^{1}H NMR (500 MHz, $CDCl_3$) δ 7.14-7.05 (m, 3 H), 6.75 (s, 1 H), 2.42 (t, *J* = 7.6 Hz, 2 H), 2.24 (s, 6 H), 1.82-1.74 (m, 2 H), 1.45-1.41 (m, 2 H), 1.38-1.31 (m, 10 H), 0.91 (t, *J* = 6.8 Hz, 3 H). ^{13}C NMR (125 MHz, $CDCl_3$) δ 171.48, 135.49, 133.90, 128.21, 127.32, 36.96, 31.87, 29.49, 29.45, 29.40, 29.28, 26.14, 22.69, 18.51, 14.12.

N-(2,6-Dimethylphenyl)cyclohexanecarboxamide (3v, Figure 2)

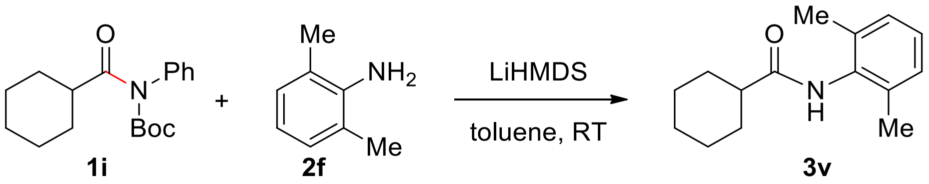

According to the general procedure, the reaction of *tert*-butyl (cyclohexanecarbonyl)(phenyl) carbamate (0.10 mmol), 2,6-dimethylaniline (2.0 equiv) and LiHMDS (1.0 M in THF, 3.0 equiv) in toluene (0.25 M) for 15 h at room temperature, afforded after work-up and chromatography the title compound in 85% yield (19.6 mg). White solid. ^{1}H NMR (500 MHz, $CDCl_3$) δ 7.17-7.03 (m, 3 H), 6.75 (s, 1 H), 2.36 (tt, *J* = 11.7, 3.4 Hz, 1 H), 2.22 (s, 6 H), 2.05 (d, *J* = 12.9 Hz, 2 H), 1.91-1.83 (m, 2 H), 1.75 (d, *J* = 12.0 Hz, 1 H), 1.64-1.56 (m, 2 H), 1.34 (td, *J* = 25.3, 12.4 Hz, 3 H). ^{13}C NMR (125 MHz, $CDCl_3$) δ 174.29, 135.47, 133.85, 128.15, 127.16, 45.87, 30.04, 25.79, 25.77, 18.41.

***N*-Phenylpivalamide (3w, Figure 2)**

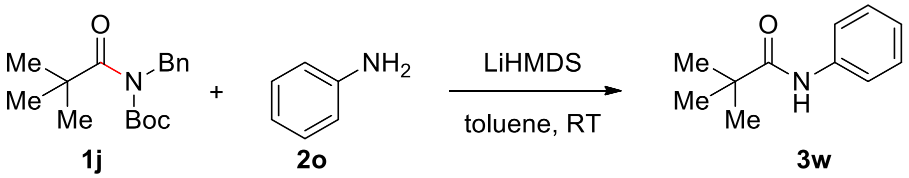

According to the general procedure, the reaction of *tert*-butyl benzyl(pivaloyl)carbamate (0.10 mmol), aniline (2.0 equiv) and LiHMDS (1.0 M in THF, 3.0 equiv) in toluene (0.25 M) for 15h at room temperature, afforded after work-up and chromatography the title compound in 93% yield (16.5 mg). White solid. ^{1}H NMR (500 MHz, $CDCl_3$) δ 7.55 (d, *J* = 8.2 Hz, 2 H), 7.34 (t, *J* = 7.8 Hz, 3 H), 7.12 (t, *J* = 7.4 Hz, 1 H), 1.34 (s, 9 H). ^{13}C NMR (125 MHz, $CDCl_3$) δ 176.58, 138.05, 128.96, 124.20, 119.98, 39.62, 27.65.

N-(2,6-Dimethylphenyl)pivalamide (3x, Figure 2)

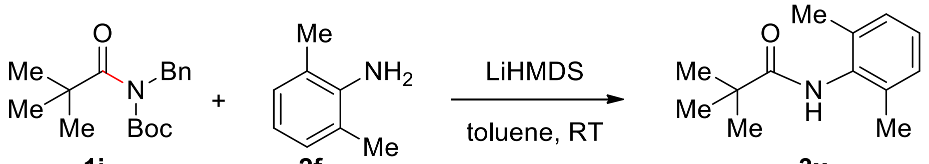

According to the general procedure, the reaction of *tert*-butyl benzyl(pivaloyl)carbamate (0.10 mmol), 2,6-dimethylaniline (2.0 equiv) and LiHMDS (1.0 M in THF, 3.0 equiv) in toluene (0.25 M) for 15 h at room temperature, afforded after work-up and chromatography the title compound in 87% yield (17.8 mg). White solid. ^{1}H NMR (500 MHz, $CDCl_3$) δ 7.14-7.02 (m, 3 H), 6.90 (s, 1 H), 2.23 (s, 6 H), 1.38 (s, 9 H). ^{13}C NMR (125 MHz, $CDCl_3$) δ 176.45, 135.41, 133.97, 128.16, 127.10, 39.30, 27.84, 18.30.

N-Phenylbenzamide (3aa, Figure 3)

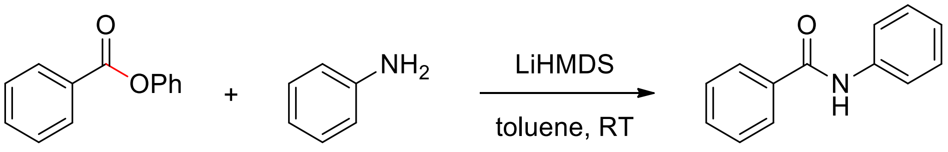

According to the general procedure, the reaction of phenyl benzoate (5.05 mmol), aniline (2.0 equiv) and LiHMDS (1.0 M in THF, 3.0 equiv) in toluene (0.25 M) for 15 h at room temperature, afforded after work-up and chromatography the title compound in 90% yield (0.895 g). White solid. ^{1}H NMR (500 MHz, $CDCl_3$) δ 7.90 (d, *J* = 7.5 Hz, 3 H), 7.67 (d, *J* = 8.1 Hz, 2 H), 7.58 (t, *J* = 7.3 Hz, 1 H), 7.51 (t, *J* = 7.5 Hz, 2 H), 7.40 (t, *J* = 7.8 Hz, 2 H), 7.18 (t, *J* = 7.4 Hz, 1 H). ^{13}C NMR (125 MHz, $CDCl_3$) δ 165.75, 137.94, 135.03, 131.87, 129.13, 128.82, 127.03, 124.60, 120.21.

N-(4-Methoxyphenyl)benzamide (3a, Figure 3)

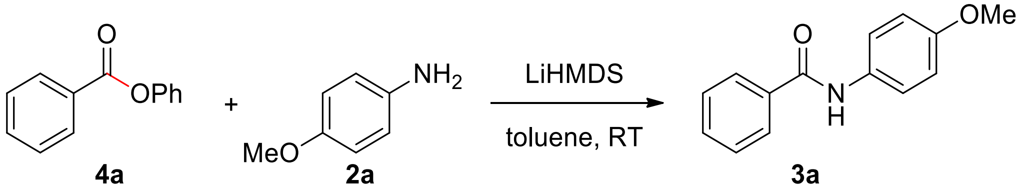

According to the general procedure, the reaction of phenyl benzoate (0.10 mmol), *p*-anisidine (2.0 equiv) and LiHMDS (1.0 M in THF, 3.0 equiv) in toluene (0.25 M) or 15h at room temperature, afforded after work-up and chromatography the title compound in 98% yield (22.2 mg). White solid. ^{1}H NMR (500 MHz, $CDCl_3$) δ 7.88 (d, J = 7.6 Hz, 2 H), 7.82 (s, 1 H), 7.57-7.55 (m, 3 H), 7.49 (t, J = 7.6 Hz, 2 H), 6.92 (d, J = 8.9 Hz, 2 H). ^{13}C NMR (125 MHz, $CDCl_3$) δ 165.64, 156.65, 135.06, 131.71, 131.02, 128.76, 127.00, 122.13, 114.26, 55.53.

N-(4-(trifluoromethyl)phenyl)benzamide (3b, Figure 3)

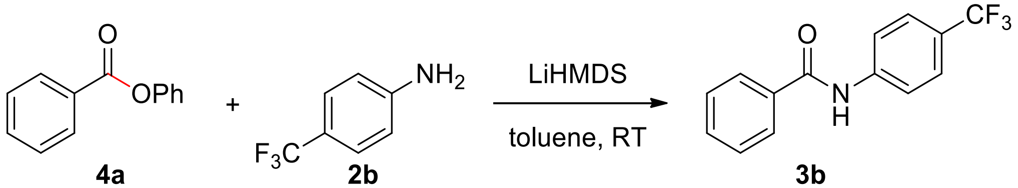

According to the general procedure, the reaction of phenyl benzoate (0.10 mmol), 4-(trifluoromethyl)aniline (2.0 equiv) and LiHMDS (1.0 M in THF, 3.0 equiv) in toluene (0.25 M) for 15 at room temperature, afforded after work-up and chromatography the title compound in 92% yield (24.4 mg). White solid. ^{1}H NMR (500 MHz, DMSO-d^6) δ 8.03 (d, J = 8.4 Hz, 2 H), 7.98 (d, J = 7.8 Hz, 2 H), 7.73 (d, J = 8.4 Hz, 2 H), 7.63 (t, J = 7.2 Hz, 1 H), 7.56 (t, J = 7.5 Hz, 2 H). ^{13}C NMR (125 MHz, DMSO-d^6) δ 166.55, 143.37, 135.00, 132.39, 128.94, 128.28, 126.39 (q, J^F = 3.8 Hz), δ 125.07 (d, J^F = 224.5 Hz), 123.86 (d, J^F = 14.9 Hz), 120.59. ^{19}F NMR (471 MHz, DMSO-d^6) δ -60.24.

Ethyl 4-benzamidobenzoate (3c, Figure 3)

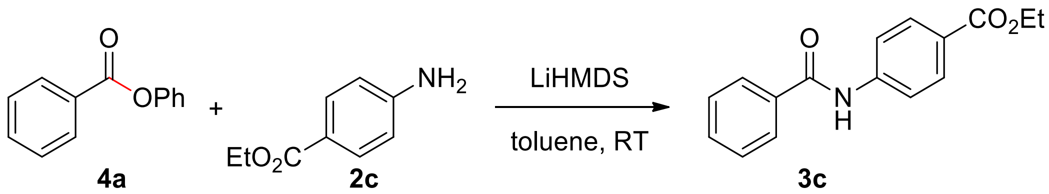

According to the general procedure, the reaction of phenyl benzoate (0.10 mmol), methyl 4-aminobenzoate (1.0 equiv) and LiHMDS (1.0 M in THF, 2.0 equiv) in toluene (0.25 M) for 15 h at room temperature, afforded after work-up and chromatography the title compound in 93% yield (23.7 mg). White solid. ^{1}H NMR (500 MHz, $CDCl_3$) δ 8.08 (d, J = 8.4 Hz, 3 H), 7.90 (d, J = 7.6 Hz, 2 H), 7.77 (d, J = 8.4 Hz, 2 H), 7.59 (t, J = 7.3 Hz, 1 H), 7.51 (t, J = 7.5 Hz, 2 H), 4.39 (q, J = 7.1 Hz, 2 H), 1.42 (t, J = 7.1 Hz, 3 H). ^{13}C NMR (125 MHz, $CDCl_3$) δ 166.14, 165.82, 142.07, 134.57, 132.23, 130.87, 128.92, 127.10, 126.23, 119.17, 60.93, 14.37.

N-(4-Hydroxyphenyl)benzamide (3d, Figure 3)

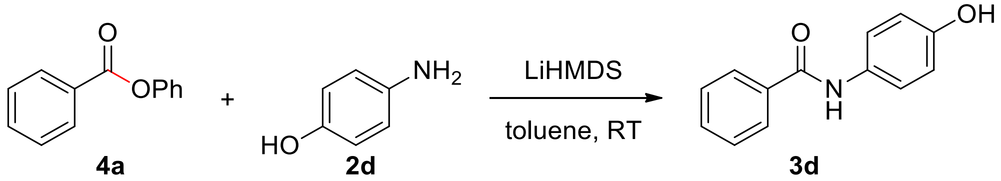

According to the general procedure, the reaction of phenyl benzoate (0.10 mmol), 4-aminophenol (2.0 equiv) and LiHMDS (1.0 M in THF, 4.0 equiv) in toluene (0.25 M) for 15 h at room temperature, afforded after work-up and chromatography the title compound in 72% yield (15.3 mg). White solid. ^{1}H NMR (500 MHz, DMSO-d^6) δ 10.02 (s, 1 H), 9.25 (s, 1 H), 7.93 (d, J = 7.4 Hz, 2 H), 7.62-7.46 (m, 5 H), 6.74 (d, J = 8.8 Hz, 2 H). ^{13}C NMR (125 MHz, DMSO-d^6) δ 165.40, 154.17, 135.64, 131.73, 131.16, 128.78, 127.96, 122.72, 115.42.

N-([1,1'-Biphenyl]-2-yl)benzamide (3e, Figure 3)

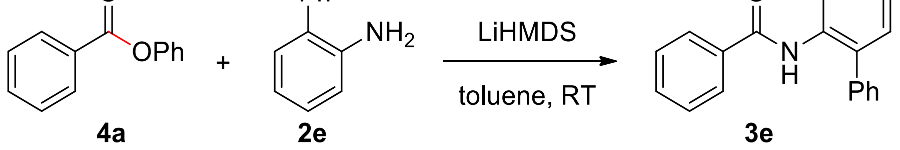

According to the general procedure, the reaction of phenyl benzoate (0.10 mmol), [1,1'-biphenyl]-2-amine (2.0 equiv) and LiHMDS (1.0 M in THF, 3.0 equiv) in toluene (0.25 M) for

15 h at room temperature, afforded after work-up and chromatography the title compound in 95% yield (25.9 mg). White solid. ^{1}H NMR (500 MHz, $CDCl_3$) δ 8.47 (d, *J* = 8.2 Hz, 1 H), 7.93 (s, 1 H), 7.53 (d, *J* = 7.9 Hz, 2 H), 7.46-7.42 (m, 2 H), 7.41-7.34 (m, 5 H), 7.31 (t, *J* = 7.6 Hz, 2 H), 7.23 (d, *J* = 7.4 Hz, 1 H), 7.15 (t, *J* = 7.5 Hz, 1 H). ^{13}C NMR (125 MHz, $CDCl_3$) δ 166.58, 138.68, 135.55, 135.41, 132.94, 132.34, 130.61, 129.99, 129.85, 129.37, 129.24, 128.81, 127.43, 124.97, 121.73.

N-(2,6-Dimethylphenyl)benzamide (3f, Figure 3)

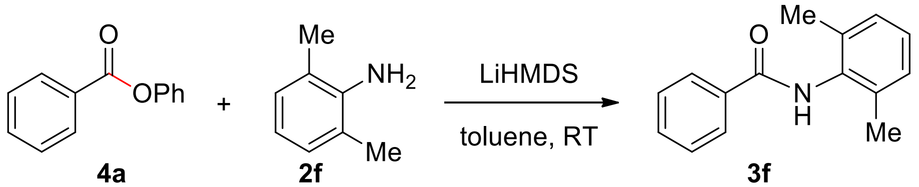

According to the general procedure, the reaction of phenyl benzoate (0.10 mmol), 2,6-dimethylaniline (2.0 equiv) and LiHMDS (1.0 M in THF, 3.0 equiv) in toluene (0.25 M) for 15 h at room temperature, afforded after work-up and chromatography the title compound in 96% yield (21.6 mg). White solid. ^{1}H NMR (500 MHz, $CDCl_3$) δ 7.95 (d, *J* = 7.6 Hz, 2 H), 7.59 (t, *J* = 7.3 Hz, 1 H), 7.52 (t, *J* = 7.6 Hz, 2 H), 7.45 (s, 1 H), 7.20-7.12 (m, 3 H), 2.31 (s, 6 H).^{13}C NMR (125 MHz, $CDCl_3$) δ 165.88, 135.58, 134.55, 133.89, 131.82, 128.79, 128.32, 127.46, 127.23, 18.52.

N-(2,6-Diisopropylphenyl)benzamide (3g, Figure 3)

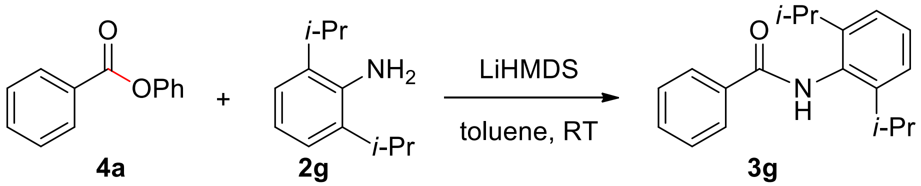

According to the general procedure, the reaction of phenyl benzoate (0.10 mmol), 2,6-diisopropylaniline (2.0 equiv) and LiHMDS (1.0 M in THF, 3.0 equiv) in toluene (0.25 M) or 15 h at room temperature, afforded after work-up and chromatography the title compound in 97% yield (27.3 mg). White solid. ^{1}H NMR (500 MHz, $CDCl_3$) δ 7.95 (d, *J* = 7.7 Hz, 2 H), 7.60 (t, *J* = 7.4 Hz, 1 H), 7.53 (t, *J* = 7.6 Hz, 2 H), 7.42-7.34 (m, 2 H), 7.25 (d, *J* = 7.7 Hz, 2 H), 3.17 (dt, *J* =

13.7, 6.9 Hz, 2 H), 1.25 (d, *J* = 6.9 Hz, 12 H). ^{13}C NMR (125 MHz, $CDCl_3$) δ 166.95, 146.41, 134.66, 131.79, 131.17, 128.84, 128.52, 127.20, 123.59, 28.93, 23.68.

N-(3-Chlorophenyl)benzamide (3h, Figure 3)

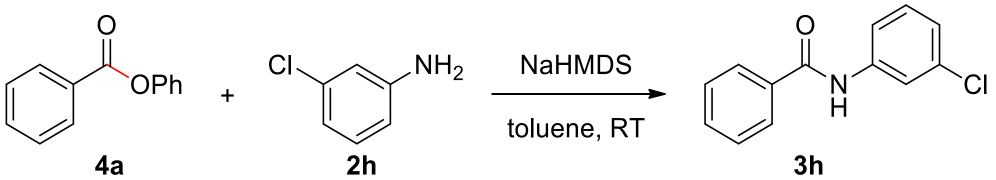

According to the general procedure, the reaction of phenyl benzoate (0.10 mmol), 3-chloroaniline (2.0 equiv) and NaHMDS (1.0 M in THF, 3.0 equiv) in toluene (0.25 M) for 15 h at room temperature, afforded after work-up and chromatography the title compound in 92% yield (21.3 mg). White solid. ^{1}H NMR (500 MHz, $CDCl_3$) δ 7.99 (s, 1 H), 7.87 (d, *J* = 7.6 Hz, 2 H), 7.79 (s, 1 H), 7.57 (t, *J* = 7.3 Hz, 1 H), 7.50 (dd, *J* = 14.0, 7.0 Hz, 3 H), 7.33-7.25 (m, 1 H), 7.14 (d, *J* = 7.8 Hz, 1 H). ^{13}C NMR (125 MHz, $CDCl_3$) δ 165.85, 139.08, 134.75, 134.54, 132.12, 130.06, 128.87, 127.06, 124.63, 120.36, 118.21.

N-(9-Ethyl-9*H*-carbazol-3-yl)benzamide (3i, Figure 3)

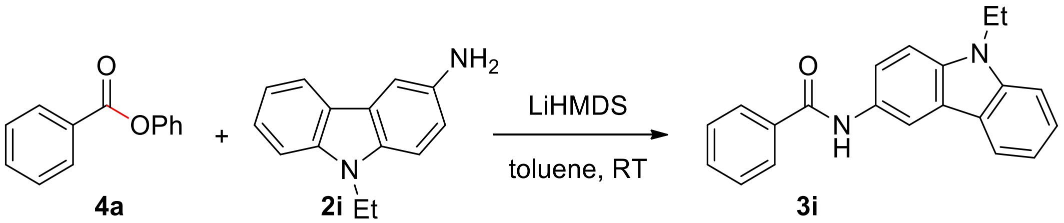

According to the general procedure, the reaction of phenyl benzoate (0.10 mmol), 9-ethyl-9*H*-carbazol-3-amine (2.0 equiv) and LiHMDS (1.0 M in THF, 3.0 equiv) in toluene (0.25 M) for 15 h at room temperature, afforded after work-up and chromatography the title compound in 85% yield (26.7 mg). White solid. ^{1}H NMR (500 MHz, $CDCl_3$) δ 8.46 (s, 1 H), 8.10 (d, *J* = 7.7 Hz, 1 H), 8.05 (s, 1 H), 7.96 (d, *J* = 7.5 Hz, 2 H), 7.66 (d, *J* = 8.4 Hz, 1 H), 7.58 (t, *J* = 7.2 Hz, 1 H), 7.55-7.47 (m, 3 H), 7.43-7.38 (m, 2 H), 7.24 (t, *J* = 7.4 Hz, 1 H), 4.37 (q, *J* = 7.2 Hz, 2 H), 1.45 (t, *J* = 7.2 Hz, 3 H). ^{13}C NMR (125 MHz, $CDCl_3$) δ 165.79, 140.49, 137.41, 135.30, 131.63, 129.71, 128.77, 127.05, 125.92, 123.12, 122.80, 120.74, 119.75, 118.81, 113.20, 108.58, 37.64, 13.84.

N-(Perfluorophenyl)benzamide (3j, Figure 3)

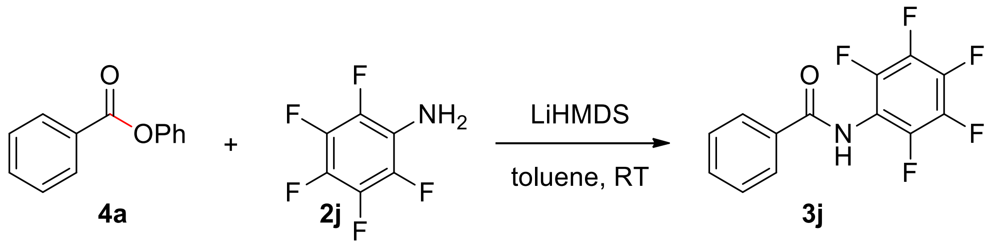

According to the general procedure, the reaction of phenyl benzoate (0.10 mmol), 2,3,4,5,6-pentafluoroaniline (2.0 equiv) and LiHMDS (1.0 M in THF, 3.0 equiv) in toluene (0.25 M) for 15 h at room temperature, afforded after work-up and chromatography the title compound in 98% yield (28.1 mg). White solid. ^{1}H NMR (500 MHz, $CDCl_3$) δ 7.94 (d, *J* = 7.4 Hz, 2 H), 7.65 (t, *J* = 7.4 Hz, 1 H), 7.55 (t, *J* = 7.7 Hz, 2 H), 7.43 (s, 1 H). ^{13}C NMR (125 MHz, $CDCl_3$) δ 165.54, 144.05 (m), 142.06 (m), 138.90 (m), 136.87 (m), 132.92, 132.39, 128.99, 127.66. ^{19}F NMR (471 MHz, $CDCl_3$) δ -144.55 – -144.65 (m), -156.36 (t, *J* = 21.3 Hz), -162.04 – -162.12 (m).

N-(Pyridin-2-yl)benzamide (3k, Figure 3)

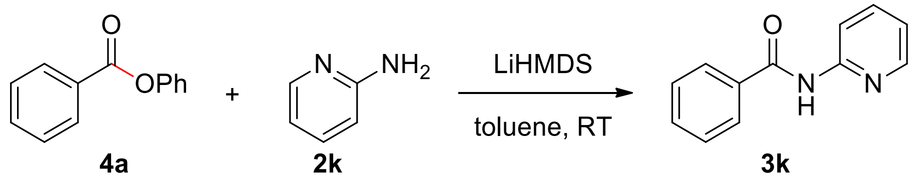

According to the general procedure, the reaction of phenyl benzoate (0.10 mmol), pyridin-2-amine (2.0 equiv) and LiHMDS (1.0 M in THF, 3.0 equiv) in toluene (0.25 M) for 15 h at room temperature, afforded after work-up and chromatography the title compound in 95% yield (18.8 mg). White solid. ^{1}H NMR (500 MHz, DMSO-d^6) δ 10.77 (s, 1 H), 8.40 (d, *J* = 3.9 Hz, 1 H), 8.20 (d, *J* = 8.3 Hz, 1 H), 8.04 (d, *J* = 7.5 Hz, 2 H), 7.89-7.83 (m, 1 H), 7.60 (t, *J* = 7.3 Hz, 1 H), 7.52 (t, *J* = 7.6 Hz, 2 H), 7.17 (dd, *J* = 7.2, 4.9 Hz, 1 H). ^{13}C NMR (125 MHz, DMSO-d^6) δ 166.45, 152.67, 148.41, 138.57, 134.56, 132.39, 128.82, 128.46, 120.28, 115.21.

N-(Quinolin-8-yl)benzamide (3l, Figure 3)

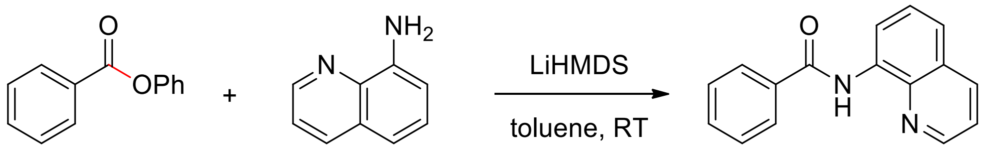

4a **2l** **3l**

According to the general procedure, the reaction of phenyl benzoate (0.10 mmol), quinolin-8-amine (2.0 equiv) and LiHMDS (1.0 M in THF, 3.0 equiv) in toluene (0.25 M) for 15 h at room temperature, afforded after work-up and chromatography the title compound in 82% yield (20.3 mg). White solid. ^{1}H NMR (500 MHz, $CDCl_3$) δ 10.77 (s, 1 H), 8.97 (dd, *J* = 7.6, 1.1 Hz, 1 H), 8.87 (dd, *J* = 4.2, 1.6 Hz, 1 H), 8.20 (dd, *J* = 8.2, 1.5 Hz, 1 H), 8.12 (dd, *J* = 8.0, 1.4 Hz, 2 H), 7.64-7.55 (m, 5 H), 7.49 (dd, *J* = 8.2, 4.2 Hz, 1 H). ^{13}C NMR (125 MHz, $CDCl_3$) δ 165.49, 148.31, 138.81, 136.41, 135.19, 134.62, 131.86, 128.82, 128.02, 127.49, 127.32, 121.71, 116.57.

_N_-Methyl-_N_-phenylbenzamide (3m, Figure 3)

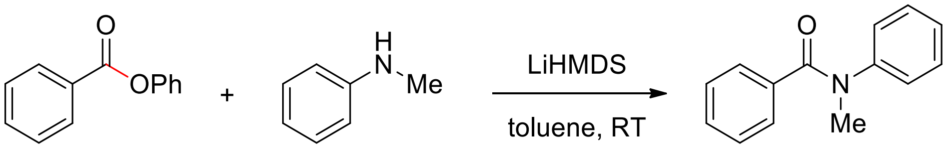

4a **2m** **3m**

According to the general procedure, the reaction of phenyl benzoate (0.10 mmol), *N*-methylaniline (2.0 equiv) and LiHMDS (1.0 M in THF, 3.0 equiv) in toluene (0.25 M) for 15 h at room temperature, afforded after work-up and chromatography the title compound in 98% yield (20.7 mg). Colorless oil. ^{1}H NMR (500 MHz, $CDCl_3$) δ 7.32 (d, *J* = 7.5 Hz, 2 H), 7.26-7.21 (m, 3 H), 7.19-7.13 (m, 3 H), 7.05 (d, *J* = 7.8 Hz, 2 H), 3.52 (s, 3 H). ^{13}C NMR (125 MHz, $CDCl_3$) δ 170.71, 144.93, 135.93, 129.60, 129.15, 128.73, 127.73, 126.93, 126.50, 38.42.

Morpholino(phenyl)methanone (3n, Figure 3)

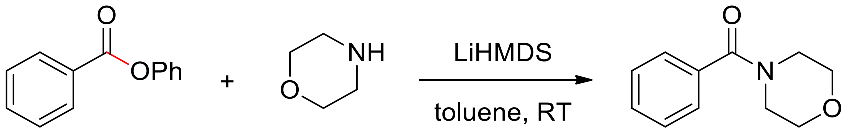

4a **2n** **3n**

According to the general procedure, the reaction of phenyl benzoate (0.10 mmol), morpholine (2.0 equiv) and LiHMDS (1.0 M in THF, 3.0 equiv) in toluene (0.25 M) for 15 h at room

temperature, afforded after work-up and chromatography the title compound in 92% yield (17.6 mg). White solid. ^{1}H NMR (500 MHz, $CDCl_3$) δ 7.43-7.42 (m, 5 H), 3.90-3.38 (m, 8 H). ^{13}C NMR (125 MHz, $CDCl_3$) δ 170.45, 135.35, 129.89, 128.58, 127.10, 66.93.

4-Methoxy-*N*-phenylbenzamide (3y, Figure 3)

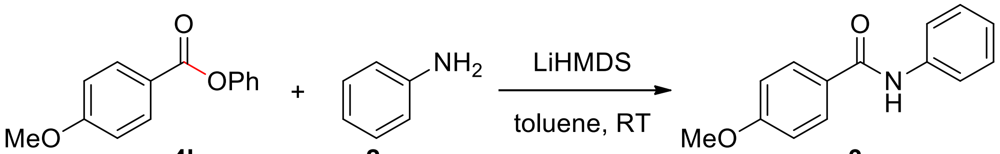

According to the general procedure, the reaction of phenyl 4-methoxybenzoate (0.10 mmol), aniline (2.0 equiv) and LiHMDS (1.0 M in THF, 3.0 equiv) in toluene (0.25 M) for 15 h at room temperature, afforded after work-up and chromatography the title compound in 97% yield (22.0 mg). White solid. ^{1}H NMR (500 MHz, $CDCl_3$) δ 7.87 (d, J = 8.7 Hz, 2 H), 7.80 (s, 1 H), 7.65 (d, J = 8.0 Hz, 2 H), 7.39 (t, J = 7.9 Hz, 2 H), 7.16 (t, J = 7.4 Hz, 1 H), 6.99 (d, J = 8.7 Hz, 2 H), 3.90 (s, 3 H). ^{13}C NMR (125 MHz, $CDCl_3$) δ 165.22, 162.50, 138.13, 129.09, 128.91, 127.18, 124.36, 120.15, 114.00, 55.49.

***N*-Phenyl-4-(trifluoromethyl)benzamide (3z, Figure 3)**

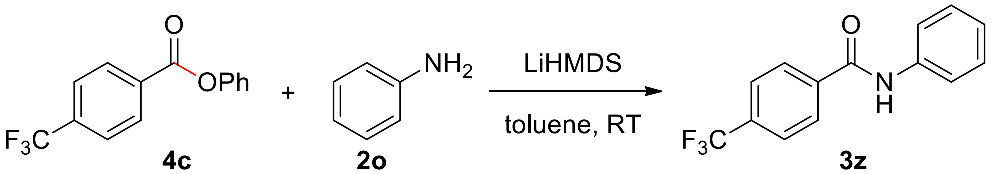

According to the general procedure, the reaction of phenyl 4-(trifluoromethyl)benzoate (0.10 mmol), aniline (2.0 equiv) and LiHMDS (1.0 M in THF, 3.0 equiv) in toluene (0.25 M) for 15 h at room temperature, afforded after work-up and chromatography the title compound in 98% yield (26.0 mg). White solid. ^{1}H NMR (500 MHz, $CDCl_3$) δ 10.47 (s, 1 H), 8.15 (d, J = 8.1 Hz, 2 H), 7.93 (d, J = 8.2 Hz, 2 H), 7.78 (d, J = 8.2 Hz, 2 H), 7.38 (t, J = 7.7 Hz, 2 H), 7.14 (t, J = 7.4 Hz, 1 H). ^{13}C NMR (125 MHz, $CDCl_3$) δ 164.86, 139.33, 139.28, 131.82 (d, J = 31.9 Hz), 129.14, 129.06, 125.84 (q, J^F = 3.7 Hz), 124.47, 120.93. ^{19}F NMR (471 MHz, $CDCl_3$) δ -61.24.

2-Methyl-*N*-phenylbenzamide (3ab, Figure 3)

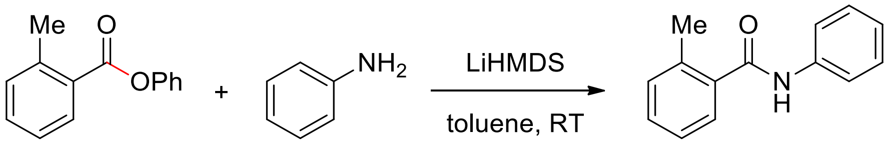

4d **2o** **3ab**

According to the general procedure, the reaction of phenyl 2-methylbenzoate (0.10 mmol), aniline (2.0 equiv) and LiHMDS (1.0 M in THF, 3.0 equiv) in toluene (0.25 M) for 15 h at room temperature, afforded after work-up and chromatography the title compound in 98% yield (20.7 mg). White solid. ^{1}H NMR (500 MHz, $CDCl_3$) δ 7.65 (d, J = 7.5 Hz, 2 H), 7.51 (d, J = 6.8 Hz, 2 H), 7.41-7.38 (m, 3 H), 7.30-7.27 (m, 2 H), 7.18 (t, J = 7.4 Hz, 1 H), 2.53 (s, 3 H). ^{13}C NMR (125 MHz, $CDCl_3$) δ 168.05, 137.99, 136.48, 131.31, 130.32, 129.15, 126.61, 125.94, 124.58, 119.87, 19.84.

3,4-Difluoro-*N*-phenylbenzamide (3ac, Figure 3)

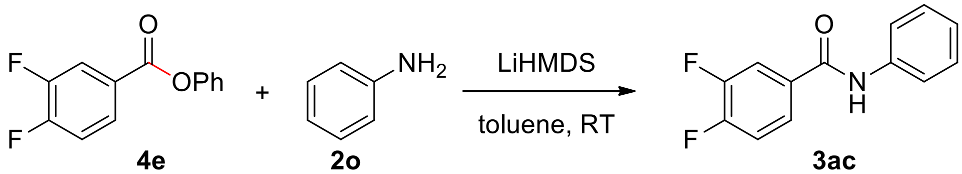

4e **2o** **3ac**

According to the general procedure, the reaction of phenyl 3,4-difluorobenzoate (0.10 mmol), aniline (2.0 equiv) and LiHMDS (1.0 M in THF, 3.0 equiv) in toluene (0.25 M) for 15 h at room temperature, afforded after work-up and chromatography the title compound in 90% yield (20.9 mg). White solid. ^{1}H NMR (500 MHz, $CDCl_3$) δ 7.82-7.71 (m, 2 H), 7.63 (d, J = 7.9 Hz, 3 H), 7.41 (t, J = 7.8 Hz, 2 H), 7.34-7.29 (m, 1 H), 7.21 (t, J = 7.4 Hz, 1 H). ^{13}C NMR (125 MHz, $CDCl_3$) δ 163.47, 151.39, 137.44, 132.03 (t, J^F = 4.3 Hz), 129.22, 125.02, 123.39 (dd, J^F = 7.1, 3.8 Hz), 120.33, 117.75 (d, J^F = 17.9 Hz), 117.03 (d, J^F = 18.6 Hz). ^{19}F NMR (471 MHz, $CDCl_3$) δ -131.70, -135.26.

***N*-Phenylfuran-2-carboxamide (3ad, Figure 3)**

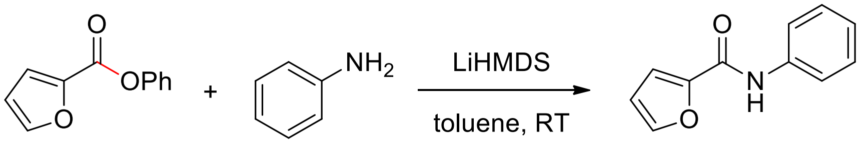

4f **2o** **3ad**

According to the general procedure, the reaction of phenyl furan-2-carboxylate (0.10 mmol), aniline (2.0 equiv) and LiHMDS (1.0 M in THF, 3.0 equiv) in toluene (0.25 M) for 15 h at room temperature, afforded after work-up and chromatography the title compound in 91% yield (17.0 mg). White solid. ^{1}H NMR (500 MHz, $CDCl_3$) δ 8.11 (s, 1 H), 7.68 (d, *J* = 7.8 Hz, 2 H), 7.53 (s, 1 H), 7.39 (t, *J* = 7.9 Hz, 2 H), 7.26 (d, *J* = 3.4 Hz, 1 H), 7.17 (t, *J* = 7.4 Hz, 1 H), 6.58 (dd, *J* = 3.3, 1.6 Hz, 1 H). ^{13}C NMR (125 MHz, $CDCl_3$) δ 156.08, 147.85, 144.18, 137.40, 129.13, 124.53, 119.93, 115.27, 112.65.

_N_-Phenyldecanamide (3ae, Figure 3)

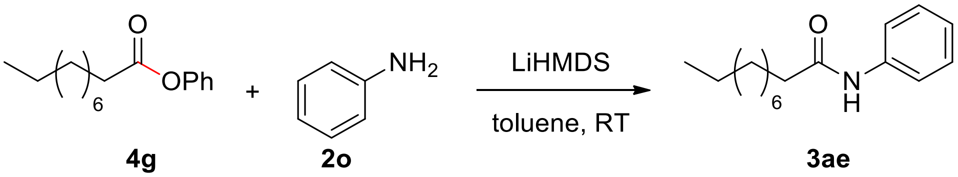

According to the general procedure, the reaction of phenyl decanoate (0.10 mmol), aniline (2.0 equiv) and LiHMDS (1.0 M in THF, 3.0 equiv) in toluene (0.25 M) for 15 h at room temperature, afforded after work-up and chromatography the title compound in 91% yield (22.5 mg). White solid. ^{1}H NMR (500 MHz, $CDCl_3$) δ 7.54 (d, *J* = 7.5 Hz, 2 H), 7.32 (d, *J* = 7.3 Hz, 2 H), 7.18-7.02 (m, 1 H), 2.37 (t, *J* = 7.5 Hz, 2 H), 1.74 (d, *J* = 5.6 Hz, 2 H), 1.37-1.28 (m, 12 H), 0.90 (*t*, *J* = 6.9 Hz, 3 H). ^{13}C NMR (125 MHz, $CDCl_3$) δ 171.48, 138.01, 128.98, 124.15, 119.79, 37.87, 31.88, 29.47, 29.41, 29.29, 25.68, 22.68, 14.12.

_N_-Phenylcyclohexanecarboxamide (3af, Figure 3)

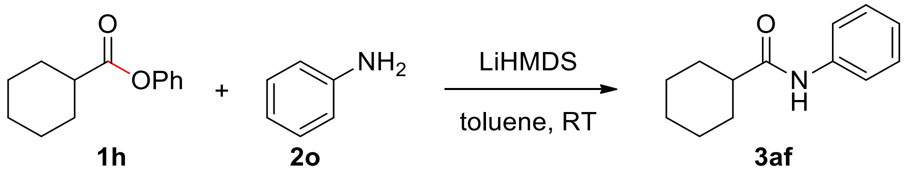

According to the general procedure, the reaction of phenyl cyclohexanecarboxylate (0.10 mmol), aniline (2.0 equiv) and LiHMDS (1.0 M in THF, 3.0 equiv) in toluene (0.25 M) for 15 h at room temperature, afforded after work-up and chromatography the title compound in 98% yield (19.9 mg). White solid. ^{1}H NMR (500 MHz, $CDCl_3$) δ 7.56 (d, *J* = 7.8 Hz, 2 H), 7.43 (s, 1 H), 7.32 (t, *J* = 7.2 Hz, 2 H), 7.11 (t, *J* = 7.3 Hz, 1 H), 2.26 (t, *J* = 11.7 Hz, 1 H), 1.97 (d, *J* = 12.7 Hz, 2 H),

1.85 (d, *J* = 10.4 Hz, 2 H), 1.72 (d, *J* = 8.3 Hz, 1H), 1.62-1.51 (m, 2 H), 1.39-1.21 (m, 3 H). ^{13}C NMR (125 MHz, $CDCl_3$) δ 174.53, 138.18, 128.96, 124.07, 119.83, 46.54, 29.68, 25.68.

_N_-Phenylpivalamide (3w, Figure 3)

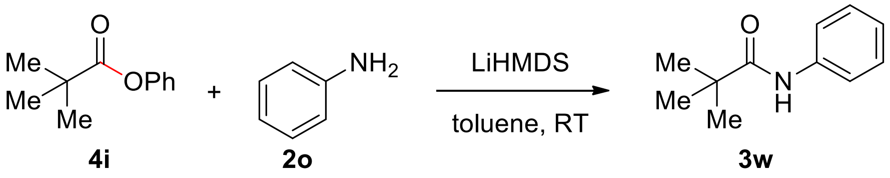

According to the general procedure, the reaction of phenyl pivalate (0.10 mmol), aniline (2.0 equiv) and LiHMDS (1.0 M in THF, 3.0 equiv) in toluene (0.25 M) for 15 h at room temperature, afforded after work-up and chromatography the title compound in 98% yield (17.3 mg). White solid. ^{1}H NMR (500 MHz, $CDCl_3$) δ 7.55 (d, *J* = 8.2 Hz, 2 H), 7.34 (t, *J* = 7.8 Hz, 3 H), 7.12 (t, *J* = 7.4 Hz, 1 H), 1.34 (s, 9 H). ^{13}C NMR (125 MHz, $CDCl_3$) δ 176.58, 138.05, 128.96, 124.20, 119.98, 39.62, 27.65.

_N_-(2,6-Dimethylphenyl)pivalamide (3x, Figure 3)

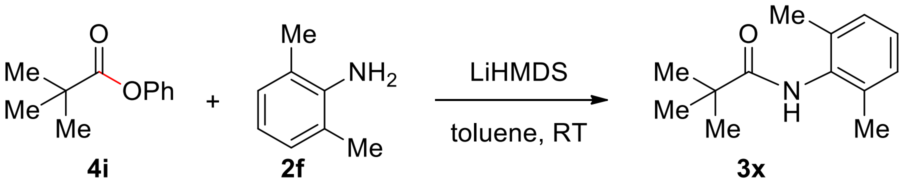

According to the general procedure, the reaction of phenyl pivalate (0.10 mmol), 2,6-dimethylaniline (2.0 equiv) and LiHMDS (1.0 M in THF, 3.0 equiv) in toluene (0.25 M) or 15 h at room temperature, afforded after work-up and chromatography the title compound in 85% yield (17.4 mg). White solid. ^{1}H NMR (500 MHz, $CDCl_3$) δ 7.14-7.02 (m, 3 H), 6.90 (s, 1 H), 2.23 (s, 6 H), 1.38 (s, 9 H). ^{13}C NMR (125 MHz, $CDCl_3$) δ 176.45, 135.41, 133.97, 128.16, 127.10, 39.30, 27.84, 18.30.

_N_-Phenylbenzamide (3aa, Figure 4)

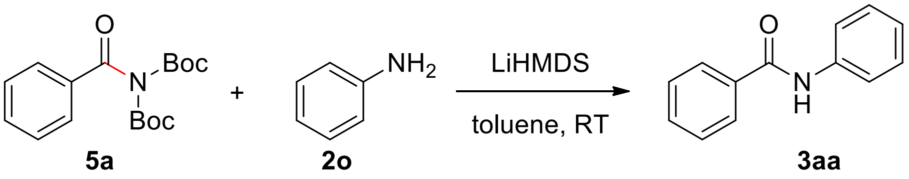

According to the general procedure, the reaction of *N*,*N*-Boc$_2$-benzamide (0.10 mmol), aniline (2.0 equiv) and LiHMDS (1.0 M in THF, 3.0 equiv) in toluene (0.25 M) for 15 h at room temperature, afforded after work-up and chromatography the title compound in 96% yield (18.9 mg). White solid. ^{1}H NMR (500 MHz, CDCl$_3$) δ 7.90 (d, *J* = 7.5 Hz, 3 H), 7.67 (d, *J* = 8.1 Hz, 2 H), 7.58 (t, *J* = 7.3 Hz, 1 H), 7.51 (t, *J* = 7.5 Hz, 2 H), 7.40 (t, *J* = 7.8 Hz, 2 H), 7.18 (t, *J* = 7.4 Hz, 1 H). ^{13}C NMR (125 MHz, CDCl$_3$) δ 165.75, 137.94, 135.03, 131.87, 129.13, 128.82, 127.03, 124.60, 120.21.

N-(4-Methoxyphenyl)benzamide (3a, Figure 4)

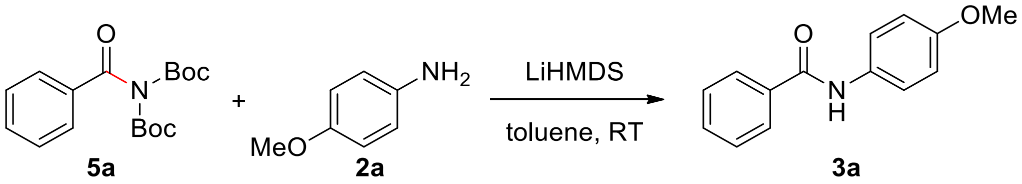

According to the general procedure, the reaction of *N*,*N*-Boc$_2$-benzamide (0.10 mmol), 4-methoxyaniline (2.0 equiv) and LiHMDS (1.0 M in THF, 3.0 equiv) in toluene (0.25 M) for 15 h at room temperature, afforded after work-up and chromatography the title compound in 97% yield (22.0 mg). White solid. ^{1}H NMR (500 MHz, CDCl$_3$) δ 7.88 (d, *J* = 7.6 Hz, 2 H), 7.82 (s, 1 H), 7.57-7.55 (m, 3 H), 7.49 (t, *J* = 7.6 Hz, 2 H), 6.92 (d, *J* = 8.9 Hz, 2 H), 3.84 (s, 3 H). ^{13}C NMR (125 MHz, CDCl$_3$) δ 165.64, 156.65, 135.06, 131.71, 131.02, 128.76, 127.00, 122.13, 114.26, 55.53.

N-(4-(Trifluoromethyl)phenyl)benzamide (3b, Figure 4)

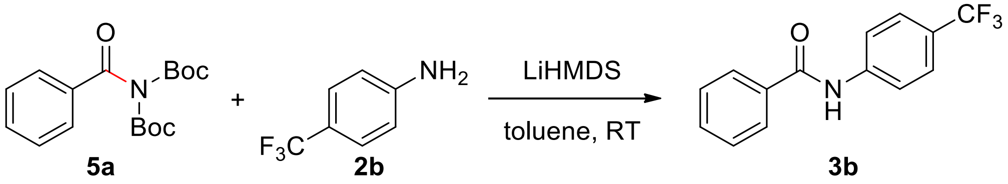

According to the general procedure, the reaction of *N*,*N*-Boc_2-benzamide (0.10 mmol), 4-(trifluoromethyl)aniline (2.0 equiv) and LiHMDS (1.0 M in THF, 3.0 equiv) in toluene (0.25 M) for 15 h at room temperature, afforded after work-up and chromatography the title compound in 91% yield (24.1 mg). ^{1}H NMR (500 MHz, DMSO-d^6) δ 8.03 (d, J = 8.4 Hz, 2 H), 7.98 (d, J = 7.8 Hz, 2 H), 7.73 (d, J = 8.4 Hz, 2 H), 7.63 (t, J = 7.2 Hz, 1 H), 7.56 (t, J = 7.5 Hz, 2 H). ^{13}C NMR (125 MHz, DMSO-d^6) δ 166.55, 143.37, 135.00, 132.39, 128.94, 128.28, 126.39 (q, J^F = 3.8 Hz), δ 125.07 (d, J^F = 224.5 Hz), 123.86 (d, J^F = 14.9 Hz), 120.59. ^{19}F NMR (471 MHz, DMSO) δ -60.24.

N-([1,1'-Biphenyl]-2-yl)benzamide (3e, Figure 4)

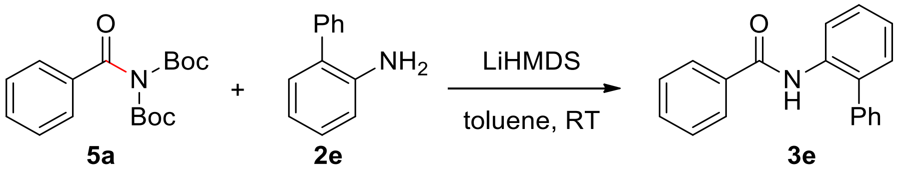

According to the general procedure, the reaction of *N*,*N*-Boc_2-benzamide (0.10 mmol), [1,1'-biphenyl]-2-amine (2.0 equiv) and LiHMDS (1.0 M in THF, 3.0 equiv) in toluene (0.25 M) for 15 h at room temperature, afforded after work-up and chromatography the title compound in 85% yield (23.2 mg). White solid. ^{1}H NMR (500 MHz, $CDCl_3$) δ 8.47 (d, J = 8.2 Hz, 1 H), 7.93 (s, 1 H), 7.53 (d, J = 7.9 Hz, 2 H), 7.46-7.42 (m, 2 H), 7.41-7.34 (m, 5 H), 7.31 (t, J = 7.6 Hz, 2 H), 7.23 (d, J = 7.4 Hz, 1 H), 7.15 (t, J = 7.5 Hz, 1 H). ^{13}C NMR (125 MHz, $CDCl_3$) δ 166.58, 138.68, 135.55, 135.41, 132.94, 132.34, 130.61, 129.99, 129.85, 129.37, 129.24, 128.81, 127.43, 124.97, 121.73.

N-(2,6-Dimethylphenyl)benzamide (3f, Figure 4)

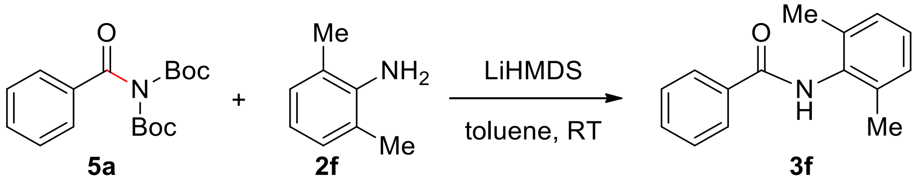

According to the general procedure, the reaction of *N*,*N*-Boc_2-benzamide (0.10 mmol), 2,6-dimethylaniline (2.0equiv) and LiHMDS (1.0 M in THF, 3.0 equiv) in toluene (0.25 M) for 15 h at room temperature, afforded after work-up and chromatography the title compound in 94%

yield (21.1 mg). White solid. ^{1}H NMR (500 MHz, $CDCl_3$) δ 7.95 (d, *J* = 7.6 Hz, 2 H), 7.59 (t, *J* = 7.3 Hz, 1 H), 7.52 (t, *J* = 7.6 Hz, 2 H), 7.45 (s, 1 H), 7.20-7.12 (m, 3 H), 2.31 (s, 6 H). ^{13}C NMR (125 MHz, $CDCl_3$) δ 165.88, 135.58, 134.55, 133.89, 131.82, 128.79, 128.32, 127.46, 127.23, 18.52.

N-(2,6-Diisopropylphenyl)benzamide (3g, Figure 4)

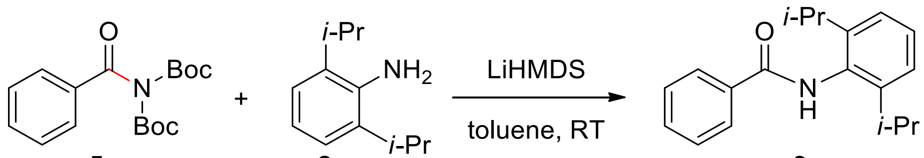

According to the general procedure, the reaction of *N,N*-Boc_2-benzamide (0.10 mmol), 2,6-diisopropylaniline (2.0 equiv) and LiHMDS (1.0 M in THF, 3.0 equiv) in toluene (0.25 M) for 15 h at room temperature, afforded after work-up and chromatography the title compound in 98% yield (24.6 mg). White solid. ^{1}H NMR (500 MHz, $CDCl_3$) δ 7.95 (d, *J* = 7.7 Hz, 2 H), 7.60 (t, *J* = 7.4 Hz, 1 H), 7.53 (t, *J* = 7.6 Hz, 2 H), 7.42-7.34 (m, 2 H), 7.25 (d, *J* = 7.7 Hz, 2 H), 3.17 (dt, *J* = 13.7, 6.9 Hz, 2 H), 1.25 (d, *J* = 6.9 Hz, 12 H). ^{13}C NMR (125 MHz, $CDCl_3$) δ 166.95, 146.41, 134.66, 131.79, 131.17, 128.84, 128.52, 127.20, 123.59, 28.93, 23.68.

N-(3-Chlorophenyl)benzamide (3h, Figure 4)

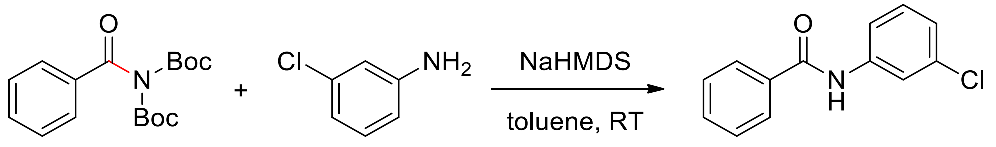

According to the general procedure, the reaction of *N,N*-Boc_2-benzamide (0.10 mmol), 3-chloroaniline (2.0 equiv) and NaHMDS (1.0 M in THF, 3.0 equiv) in toluene (0.25 M) for 15 h at room temperature, afforded after work-up and chromatography the title compound in 96% yield (22.3 mg). White solid. ^{1}H NMR (500 MHz, $CDCl_3$) δ 7.99 (s, 1 H), 7.87 (d, *J* = 7.6 Hz, 2 H), 7.79 (s, 1 H), 7.57 (t, *J* = 7.3 Hz, 1 H), 7.50 (dd, *J* = 14.0, 7.0 Hz, 3 H), 7.33-7.25 (m, 1 H), 7.14 (d, *J* = 7.8 Hz, 1 H). ^{13}C NMR (125 MHz, $CDCl_3$) δ 165.85, 139.08, 134.75, 134.54, 132.12, 130.06, 128.87, 127.06, 124.63, 120.36, 118.21.

***N*-(9-Ethyl-9*H*-carbazol-3-yl)benzamide (3i, Figure 4)**

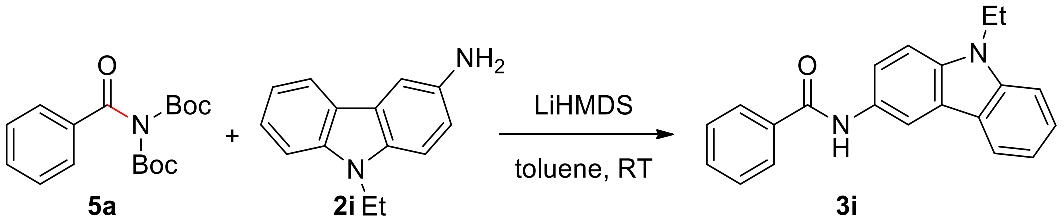

According to the general procedure, the reaction of *N*,*N*-Boc_2-benzamide (0.10 mmol), 9-ethyl-9*H*-carbazol-3-amine (2.0 equiv) and LiHMDS (1.0 M in THF, 3.0 equiv) in toluene (0.25 M) for 15 h at room temperature, afforded after work-up and chromatography the title compound in 87% yield (27.3 mg). White solid. ^1H NMR (500 MHz, $CDCl_3$) δ 8.46 (s, 1 H), 8.10 (d, *J* = 7.7 Hz, 1 H), 8.05 (s, 1 H), 7.96 (d, *J* = 7.5 Hz, 2 H), 7.66 (d, *J* = 8.4 Hz, 1 H), 7.58 (t, *J* = 7.2 Hz, 1 H), 7.55-7.47 (m, 3 H), 7.43-7.38 (m, 2 H), 7.24 (t, *J* = 7.4 Hz, 1 H), 4.37 (q, *J* = 7.2 Hz, 2 H), 1.45 (t, *J* = 7.2 Hz, 3 H). ^{13}C NMR (125 MHz, $CDCl_3$) δ 165.79, 140.49, 137.41, 135.30, 131.63, 129.71, 128.77, 127.05, 125.92, 123.12, 122.80, 120.74, 119.75, 118.81, 113.20, 108.58, 37.64, 13.84.

***N*-(Perfluorophenyl)benzamide (3j, Figure 4)**

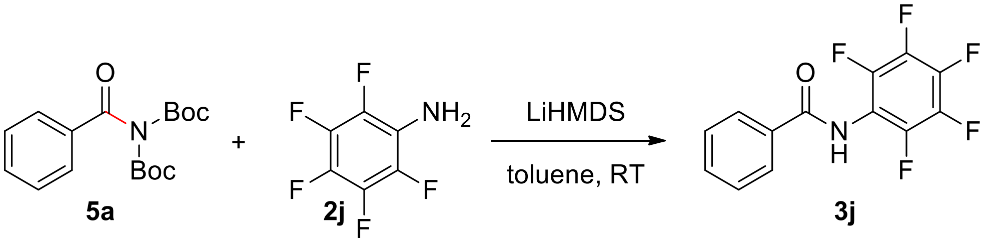

According to the general procedure, the reaction of *N*,*N*-Boc_2-benzamide (0.10 mmol), 2,3,4,5,6-pentafluoroaniline (2.0 equiv) and LiHMDS (1.0 M in THF, 3.0 equiv) in toluene (0.25 M) for 15 h at room temperature, afforded after work-up and chromatography the title compound in 88% yield (25.3 mg). White solid. ^1H NMR (500 MHz, $CDCl_3$) δ 7.94 (d, *J* = 7.4 Hz, 2 H), 7.65 (t, *J* = 7.4 Hz, 1 H), 7.55 (t, *J* = 7.7 Hz, 2 H), 7.43 (s, 1 H). ^{13}C NMR (125 MHz, $CDCl_3$) δ 165.54, 144.05 (m), 142.06 (m), 138.90 (m), 136.87 (m), 132.92, 132.39, 128.99, 127.66. ^{19}F NMR (471 MHz, $CDCl_3$) δ -144.55 – -144.65 (m), -156.36 (t, *J* = 21.3 Hz), -162.04 – -162.12 (m).

N-(Quinolin-8-yl)benzamide (3l, Figure 4)

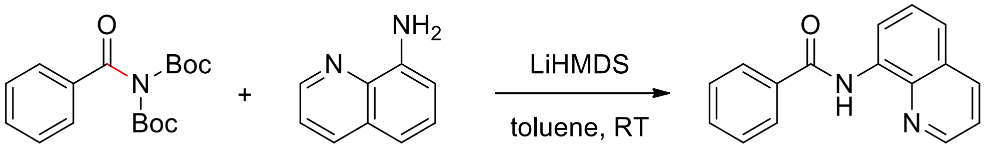

5a **2l** **3l**

According to the general procedure, the reaction of *N*,*N*-Boc$_2$-benzamide (0.10 mmol), quinolin-8-amine (2.0 equiv) and LiHMDS (1.0 M in THF, 3.0 equiv) in toluene (0.25 M) for 15 h at room temperature, afforded after work-up and chromatography the title compound in 92% yield (22.8 mg). White solid. ^{1}H NMR (500 MHz, CDCl$_3$) δ 10.77 (s, 1 H), 8.97 (dd, *J* = 7.6, 1.1 Hz, 1 H), 8.87 (dd, *J* = 4.2, 1.6 Hz, 1 H), 8.20 (dd, *J* = 8.2, 1.5 Hz, 1 H), 8.12 (dd, *J* = 8.0, 1.4 Hz, 2 H), 7.64-7.55 (m, 5 H), 7.49 (dd, *J* = 8.2, 4.2 Hz, 1 H). ^{13}C NMR (125 MHz, CDCl$_3$) δ 165.49, 148.31, 138.81, 136.41, 135.19, 134.62, 131.86, 128.82, 128.02, 127.49, 127.32, 121.71, 116.57.

N-Methyl-N-phenylbenzamide (3m, Figure 4)

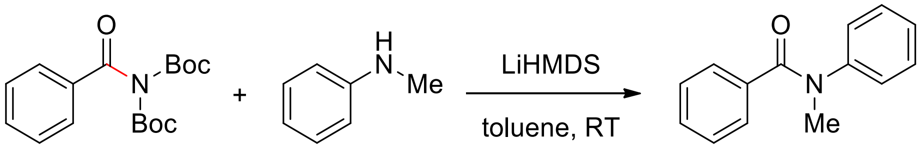

5a **2m** **3m**

According to the general procedure, the reaction of *N*,*N*-Boc$_2$-benzamide (0.10 mmol), *N*-methylaniline (2.0 equiv) and LiHMDS (1.0 M in THF, 3.0 equiv) in toluene (0.25 M) for 15 h at room temperature, afforded after work-up and chromatography the title compound in 96% yield (20.3 mg). Colorless oil. ^{1}H NMR (500 MHz, CDCl$_3$) δ 7.32 (d, *J* = 7.5 Hz, 2 H), 7.26-7.21 (m, 3 H), 7.19-7.13 (m, 3 H), 7.05 (d, *J* = 7.8 Hz, 2 H), 3.52 (s, 3 H). ^{13}C NMR (125 MHz, CDCl$_3$) δ 170.71, 144.93, 135.93, 129.60, 129.15, 128.73, 127.73, 126.93, 126.50, 38.42.

Morpholino(phenyl)methanone (3n, Figure 4)

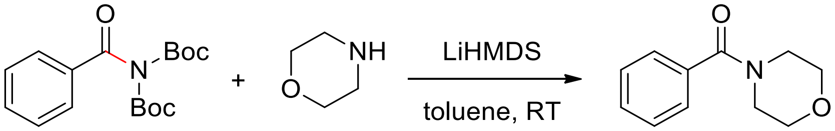

5a **2n** **3n**

According to the general procedure, the reaction of *N*,*N*-Boc$_2$-benzamide (0.10 mmol), morpholine (2.0 equiv) and LiHMDS (1.0 M in THF, 3.0 equiv) in toluene (0.25 M) for 15 h at room temperature, afforded after work-up and chromatography the title compound in 70% yield (13.4 mg). White solid. ^{1}H NMR (500 MHz, CDCl$_3$) δ 7.43-7.42 (m, 5 H), 3.90-3.38 (m, 8 H). ^{13}C NMR (125 MHz, CDCl$_3$) δ 170.45, 135.35, 129.89, 128.58, 127.10, 66.93.

Ethyl 4-benzamidobenzoate (3c, Figure 4)

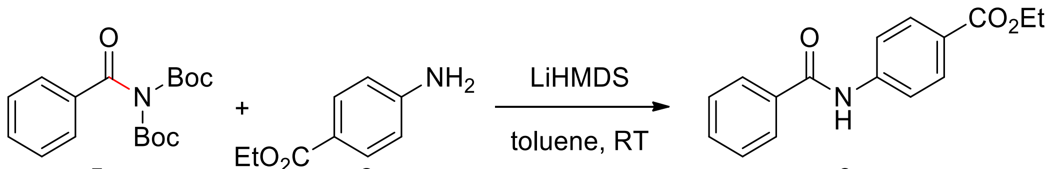

5a 2c 3c

According to the general procedure, the reaction of *N*,*N*-Boc$_2$-benzamide (0.10 mmol), ethyl 4-aminobenzoate (2.0 equiv) and LiHMDS (1.0 M in THF, 3.0 equiv) in toluene (0.25 M) for 15 h at room temperature, afforded after work-up and chromatography the title compound in 74% yield (18.0 mg). White solid. ^{1}H NMR (500 MHz, CDCl$_3$) δ 8.08 (d, *J* = 8.4 Hz, 3 H), 7.90 (d, *J* = 7.6 Hz, 2 H), 7.77 (d, *J* = 8.4 Hz, 2 H), 7.59 (t, *J* = 7.3 Hz, 1 H), 7.51 (t, *J* = 7.5 Hz, 2 H), 4.39 (q, *J* = 7.1 Hz, 2 H), 1.42 (t, *J* = 7.1 Hz, 3 H). ^{13}C NMR (125 MHz, CDCl$_3$) δ 166.14, 165.82, 142.07, 134.57, 132.23, 130.87, 128.92, 127.10, 126.23, 119.17, 60.93, 14.37.

4-Hydroxy-*N*-phenylbenzamide (3ag, Figure 4)

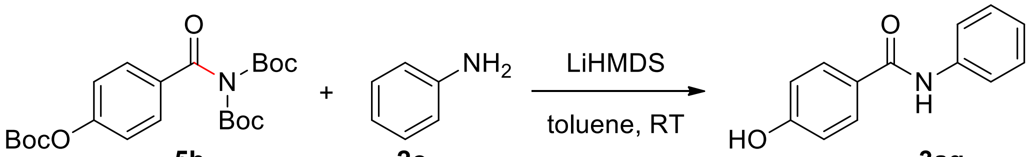

5b 2o 3ag

According to the general procedure, the reaction of *N*,*N*-Boc$_2$-4-(*tert*-butoxycarbonylhydroxy) benzamide (0.10 mmol), aniline (2.0 equiv) and LiHMDS (1.0 M in THF, 3.0 equiv) in toluene (0.25 M) for 15 h at room temperature, afforded after work-up and chromatography the title compound in 80% yield (18.9 mg). White solid. ^{1}H NMR (500 MHz, DMSO-d^6) δ 10.10 (s, 1 H), 9.98 (s, 1 H), 7.86 (d, *J* = 8.6 Hz, 2 H), 7.76 (d, *J* = 7.9 Hz, 2 H), 7.33 (t, *J* = 7.8 Hz, 2 H), 7.07 (t, *J* = 7.3 Hz, 1 H), 6.87 (d, *J* = 8.6 Hz, 2 H). ^{13}C NMR (125 MHz, DMSO-d^6) δ 165.56, 161.00, 139.94, 130.17, 128.98, 125.89, 123.72, 120.73, 115.36.

N-Phenylthiophene-3-carboxamide (3ah, Figure 4)

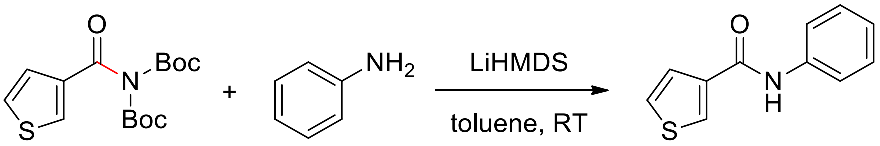

5c **2o** **3ah**

According to the general procedure, the reaction of *N,N*-Boc$_2$-thiophene-3-carboxamide (0.10 mmol), aniline (2.0 equiv) and LiHMDS (1.0 M in THF, 3.0 equiv) in toluene (0.25 M) for 15 h at room temperature, afforded after work-up and chromatography the title compound in 93% yield (18.9 mg). White solid. ^{1}H NMR (500 MHz, CDCl$_3$) δ 8.00 (d, *J* = 1.8 Hz, 1 H), 7.77 (s, 1 H), 7.64 (d, *J* = 7.9 Hz, 2 H), 7.52 (d, *J* = 4.4 Hz, 1 H), 7.43-7.35 (m, 3 H), 7.17 (t, *J* = 7.4 Hz, 1 H). ^{13}C NMR (125 MHz, CDCl$_3$) δ 161.16, 137.84, 137.76, 129.11, 128.69, 126.91, 126.14, 124.57, 120.25.

4-Methyl-*N*-phenylbenzamide (3ai, Figure 4)

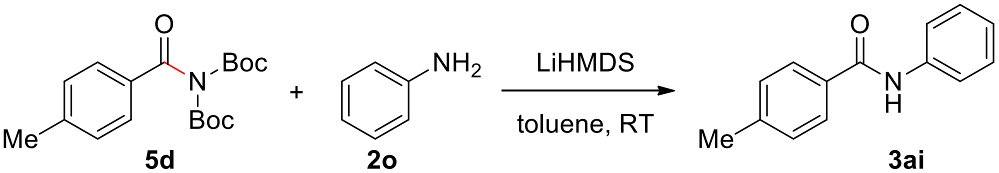

5d **2o** **3ai**

According to the general procedure, the reaction of *N,N*-Boc$_2$-4-methylbenzamide (0.10 mmol), aniline (2.0 equiv) and LiHMDS (1.0 M in THF, 3.0 equiv) in toluene (0.25 M) for 15 h at room temperature, afforded after work-up and chromatography the title compound in 85% yield (17.9 mg). White solid. ^{1}H NMR (500 MHz, CDCl$_3$) δ 7.88 (s, 1 H), 7.79 (d, *J* = 8.0 Hz, 2 H), 7.67 (d, *J* = 8.1 Hz, 2 H), 7.38 (t, *J* = 7.7 Hz, 2 H), 7.29 (d, *J* = 8.5 Hz, 2 H), 7.17 (t, *J* = 7.4 Hz, 1 H), 2.44 (s, 3 H). ^{13}C NMR (125 MHz, CDCl$_3$) δ 165.71, 142.38, 138.07, 132.14, 129.45, 129.09, 127.06, 124.44, 120.20, 21.52.

2-Ethoxy-*N*-phenylbenzamide (3aj, Figure 4)

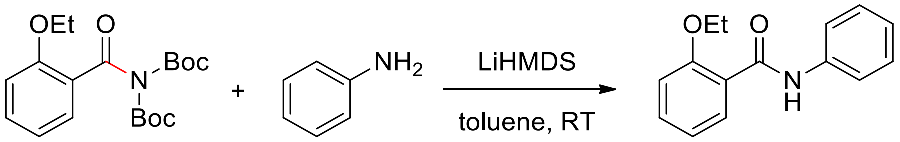

5e **2o** **3aj**

According to the general procedure, the reaction of *N,N*-Boc_2-ethenzamide (0.10 mmol), aniline (2.0 equiv) and LiHMDS (1.0 M in THF, 3.0 equiv) in toluene (0.25 M) for 15 h at room temperature, afforded after work-up and chromatography the title compound in 90% yield (21.7 mg). White solid. ^{1}H NMR (500 MHz, $CDCl_3$) δ 10.16 (s, 1 H), 8.34 (dd, *J* = 7.8, 1.6 Hz, 1 H), 7.71 (d, *J* = 8.1 Hz, 2 H), 7.52-7.47 (m, 1 H), 7.39 (t, *J* = 7.8 Hz, 2 H), 7.15 (dd, *J* = 10.5, 4.5 Hz, 2 H), 7.03 (d, *J* = 8.3 Hz, 1 H), 4.31 (q, *J* = 6.9 Hz, 2 H), 1.67 (t, *J* = 7.0 Hz, 3 H). ^{13}C NMR (125 MHz, $CDCl_3$) δ 163.22, 156.72, 138.69, 133.20, 132.54, 129.07, 124.00, 121.71, 121.71, 120.02, 112.44, 65.08, 14.98.

_N_-Phenylnicotinamide (3ak, Figure 4)

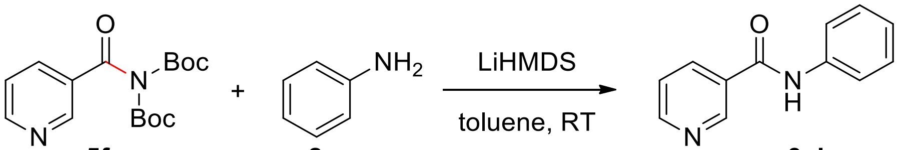

5f **2o** **3ak**

According to the general procedure, the reaction of *N,N*-Boc_2-nicotinamide (0.10 mmol), aniline (2.0 equiv) and LiHMDS (1.0 M in THF, 3.0 equiv) in toluene (0.25 M) for 15 h at room temperature, afforded after work-up and chromatography the title compound in 93% yield (18.4 mg). White solid. ^{1}H NMR (500 MHz, $CDCl_3$) δ 9.12 (s, 1 H), 8.78 (d, *J* = 4.0 Hz, 1 H), 8.23 (d, *J* = 7.9 Hz, 1 H), 8.12 (s, 1 H), 7.67 (d, *J* = 7.9 Hz, 2 H), 7.45 (dd, *J* = 7.7, 4.9 Hz, 1 H), 7.41 (t, *J* = 7.8 Hz, 2 H), 7.21 (t, *J* = 7.4 Hz, 1 H). ^{13}C NMR (125 MHz, $CDCl_3$) δ 163.88, 152.47, 147.85, 137.48, 135.44, 130.85, 129.21, 125.10, 123.74, 120.48.

_N_-Phenylbenzamide (3aa, Figure 5)

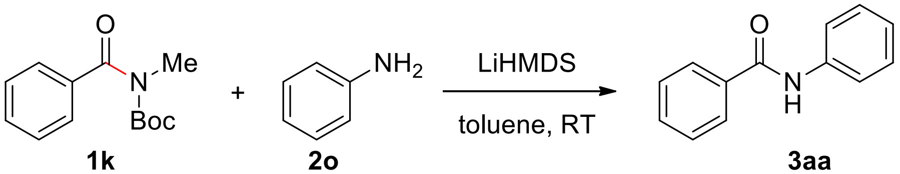

1k **2o** **3aa**

According to the general procedure, the reaction of *tert*-butyl benzoyl(methyl)carbamate (4.25 mmol), aniline (2.0 equiv) and LiHMDS (1.0 M in THF, 3.0 equiv) in toluene (0.25 M) for 15 h at room temperature, afforded after work-up and chromatography the title compound in 91% yield (0.770 g). White solid. ^{1}H NMR (500 MHz, $CDCl_3$) δ 7.90 (d, *J* = 7.5 Hz, 3 H), 7.67 (d, *J* =

8.1 Hz, 2 H), 7.58 (t, *J* = 7.3 Hz, 1 H), 7.51 (t, *J* = 7.5 Hz, 2 H), 7.40 (t, *J* = 7.8 Hz, 2H), 7.18 (t, *J* = 7.4 Hz, 1 H). ^{13}C NMR (125 MHz, $CDCl_3$) δ 165.75, 137.94, 135.03, 131.87, 129.13, 128.82, 127.03, 124.60, 120.21.

4-Bromo-*N*-phenylbenzamide (3al, Figure 5)

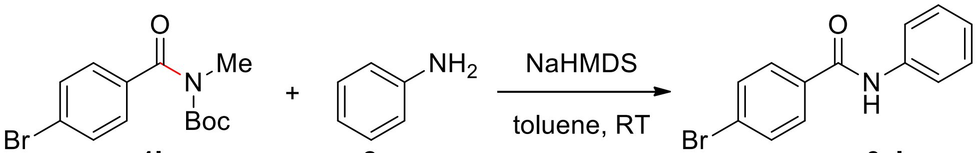

According to the general procedure, the reaction of *tert*-butyl (4-bromobenzoyl)(methyl) carbamate (0.10 mmol), aniline (2.0 equiv) and NaHMDS (1.0 M in THF, 3.0 equiv) in toluene (0.25 M) for 15 h at room temperature, afforded after work-up and chromatography the title compound in 98% yield (27.0 mg). White solid. ^{1}H NMR (500 MHz, $CDCl_3$) δ 7.77 (d, *J* = 8.1 Hz, 3 H), 7.66-7.64 (m, 4 H), 7.41 (t, *J* = 7.8 Hz, 2 H), 7.20 (t, *J* = 7.4 Hz, 1 H). ^{13}C NMR (125 MHz, $CDCl_3$) δ 164.72, 137.64, 133.83, 132.08, 129.19, 128.63, 126.63, 124.85, 120.25.

***N*-Phenylbenzamide (3aa, Figure 5)**

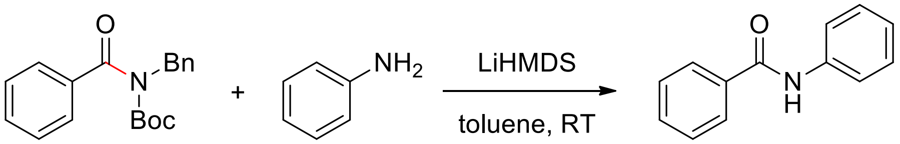

According to the general procedure, the reaction of *tert*-butyl benzoyl(benzyl)carbamate (0.10 mmol), aniline (2.0 equiv) and LiHMDS (1.0 M in THF, 3.0 equiv) in toluene (0.25 M) for 15 h at room temperature, afforded after work-up and chromatography the title compound in 90% yield (17.7 mg). White solid. ^{1}H NMR (500 MHz, $CDCl_3$) δ 7.90 (d, *J* = 7.5 Hz, 3 H), 7.67 (d, *J* = 8.1 Hz, 2 H), 7.58 (t, *J* = 7.3 Hz, 1 H), 7.51 (t, *J* = 7.5 Hz, 2 H), 7.40 (t, *J* = 7.8 Hz, 2 H), 7.18 (t, *J* = 7.4 Hz, 1 H). ^{13}C NMR (125 MHz, $CDCl_3$) δ 165.75, 137.94, 135.03, 131.87, 129.13, 128.82, 127.03, 124.60, 120.21.

***N*-Phenylbenzamide (3aa, Figure 5)**

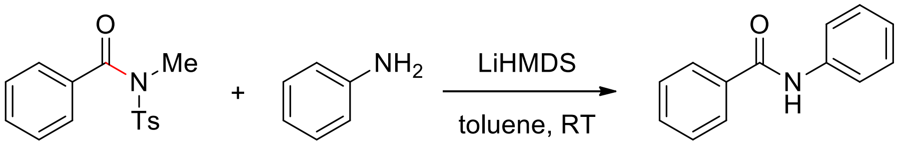

1n 2o 3aa

According to the general procedure, the reaction of *N*-methyl-*N*-tosylbenzamide (0.10 mmol), aniline (2.0 equiv) and LiHMDS (1.0 M in THF, 3.0 equiv) in toluene (0.25 M) for 15 h at room temperature, afforded after work-up and chromatography the title compound in 97% yield (19.1 mg). White solid. ^{1}H NMR (500 MHz, $CDCl_3$) δ 7.90 (d, *J* = 7.5 Hz, 3 H), 7.67 (d, *J* = 8.1 Hz, 2 H), 7.58 (t, *J* = 7.3 Hz, 1 H), 7.51 (t, *J* = 7.5 Hz, 2 H), 7.40 (t, *J* = 7.8 Hz, 2 H), 7.18 (t, *J* = 7.4 Hz, 1 H). ^{13}C NMR (125 MHz, $CDCl_3$) δ 165.75, 137.94, 135.03, 131.87, 129.13, 128.82, 127.03, 124.60, 120.21.

_N_-(4-Methoxyphenyl)benzamide (3a, Figure 5)

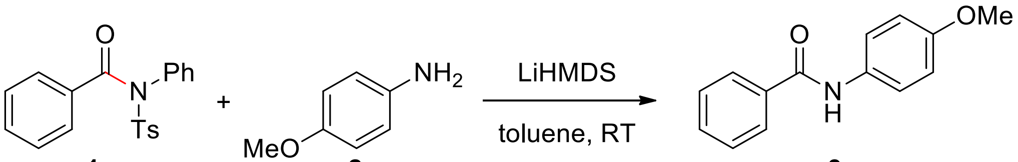

1o 2a 3a

According to the general procedure, the reaction of *N*-phenyl-*N*-tosylbenzamide (0.10 mmol), 4-methoxyaniline (2.0 equiv) and LiHMDS (1.0 M in THF, 3.0 equiv) in toluene (0.25 M) for 15 h at room temperature, afforded after work-up and chromatography the title compound in 90% yield (20.4 mg). White solid. ^{1}H NMR (500 MHz, $CDCl_3$) δ 7.88 (d, *J* = 7.6 Hz, 2 H), 7.82 (s, 1 H), 7.57-7.55 (m, 3 H), 7.49 (t, *J* = 7.6 Hz, 2 H), 6.92 (d, *J* = 8.9 Hz, 2 H). ^{13}C NMR (125 MHz, $CDCl_3$) δ 165.64, 156.65, 135.06, 131.71, 131.02, 128.76, 127.00, 122.13, 114.26, 55.53.

_N_-(4-Methoxyphenyl)benzamide (3a, Figure 5)

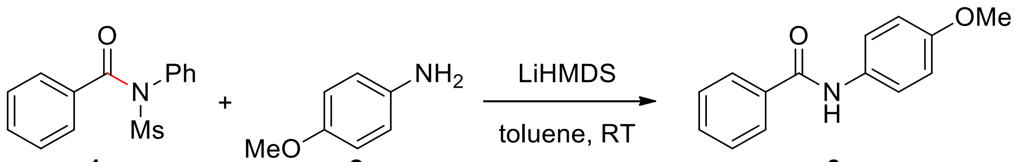

1p 2a 3a

According to the general procedure, the reaction of *N*-(methylsulfonyl)-*N*-phenylbenzamide (0.10 mmol), 4-methoxyaniline (2.0 equiv) and LiHMDS (1.0 M in THF, 3.0 equiv) in toluene

(0.25 M) for 15 h at room temperature, afforded after work-up and chromatography the title compound in 70% yield (15.9 mg). White solid. ^{1}H NMR (500 MHz, $CDCl_3$) δ 7.88 (d, *J* = 7.6 Hz, 2 H), 7.82 (s, 1 H), 7.57-7.55 (m, 3 H), 7.49 (t, *J* = 7.6 Hz, 2 H), 6.92 (d, *J* = 8.9 Hz, 2 H), 3.84 (s, 3 H). ^{13}C NMR (125 MHz, $CDCl_3$) δ 165.64, 156.65, 135.06, 131.71, 131.02, 128.76, 127.00, 122.13, 114.26, 55.53.

_N_-Phenylbenzamide (3aa, Figure 5)

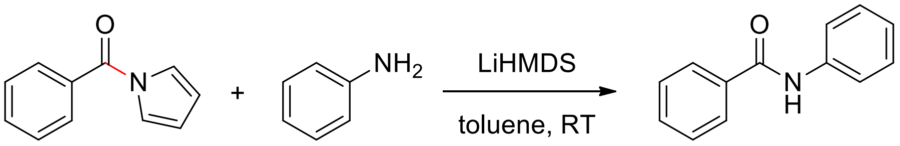

According to the general procedure, the reaction of phenyl(1*H*-pyrrol-1-yl)methanone (0.10 mmol), aniline (2.0 equiv) and LiHMDS (1.0 M in THF, 3.0 equiv) in toluene (0.25 M) for 15 h at room temperature, afforded after work-up and chromatography the title compound in 98% yield (19.3 mg). White solid. ^{1}H NMR (500 MHz, $CDCl_3$) δ 7.90 (d, *J* = 7.5 Hz, 3 H), 7.67 (d, *J* = 8.1 Hz, 2 H), 7.58 (t, *J* = 7.3 Hz, 1 H), 7.51 (t, *J* = 7.5 Hz, 2 H), 7.40 (t, *J* = 7.8 Hz, 2 H), 7.18 (t, *J* = 7.4 Hz, 1 H). ^{13}C NMR (125 MHz, $CDCl_3$) δ 165.75, 137.94, 135.03, 131.87, 129.13, 128.82, 127.03, 124.60, 120.21.

(_R_)-_tert_-Butyl 2-(phenylcarbamoyl)pyrrolidine-1-carboxylate (3am, Figure 6)

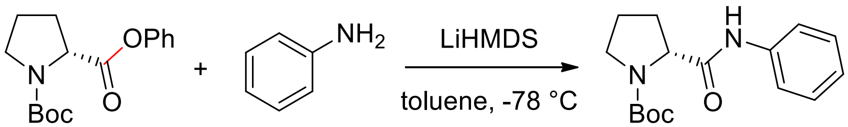

According to the general procedure, the reaction of (*R*)-1-*tert*-butyl 2-phenyl pyrrolidine-1,2-dicarboxylate (0.10 mmol), aniline (1.0 equiv) and LiHMDS (1.0 M in THF, 2.0 equiv) in toluene (0.25 M) for 30 min at -78 °C, afforded after work-up and chromatography the title compound in 90% yield (26.1 mg). 97.5% ee. White solid. ^{1}H NMR (500 MHz, $CDCl_3$) δ 9.50 (s, 1 H), 7.54 (d, *J* = 8.0 Hz, 2 H), 7.35-7.29 (m, 2 H), 7.10 (s, 1 H), 4.50 (s, 1 H), 3.60-3.38 (m, 2 H), 2.57 (s, 1 H), 2.07-1.94 (m, 3 H), 1.52 (s, 9 H). ^{13}C NMR (125 MHz, $CDCl_3$) δ 169.83, 156.69, 138.45, 128.95, 123.83, 119.67, 80.94, 60.50, 47.24, 28.41, 27.00, 24.66. HPLC analysis

(chiralcel OD-H, *n*-hexane/*i*-PrOH 90/10, 1.0 mL/min, 254 nm) indicated 97.5% ee: t_R (major) = 6.14 minutes, t_R (minor) = 7.27 minutes.

One-pot *N*-Activation/Transamidation

N-(2,4,6-Trichlorophenyl)benzamide (3an, Figure 7)

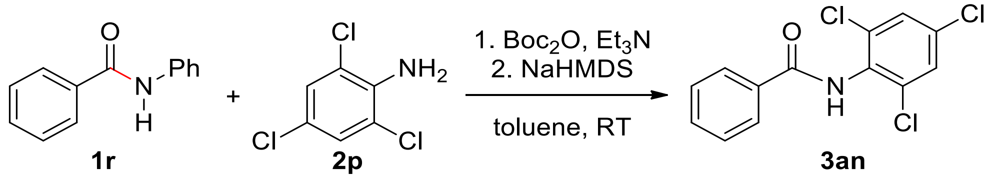

An oven-dried round-bottomed flask (25 mL) was charged with *N*-phenylbenzamide (2.0 mmol, 1.0 equiv), DMAP (0.10 equiv) and dichloromethane (0.20 M). Boc_2O (1.3 equiv) was added in one portion and the reaction mixture was stirred for 15 h at room temperature. After the indicated time, the reaction mixture was quenched with $NaHCO_3$ (aq., sat., 10 mL), extracted with CH_2Cl_2 (2 x 20 mL), washed with water (1 x 20 mL), brine (1 x 20 mL), the organic layers were combined, dried, and concentrated. The crude product was used in next step without further purification. According to the general procedure, an oven-dried vial equipped with a stir bar was charged with the above crude product and 2,4,6-trichloroaniline (2.0 equiv, 4.0 mmol) placed under a positive pressure of argon, and subjected to three evacuation/backfilling cycles. Toluene (0.25 M) and NaHMDS (1.0 M in THF, 3.0 equiv) were added with vigorous stirring at room temperature, and the reaction mixture was stirred for 15 h at room temperature After the indicated time, the reaction mixture was quenched with NH_4Cl (aq., 1.0 M, 20 mL), extracted with EtOAc (3 x 50 mL), the organic layers were combined, washed with water (1 x 50 mL), brine (1 x 50 mL), dried over Na_2SO_4 and concentrated. Purification by chromatography on silica gel (EtOAc/hexanes) afforded the title product. Yield 93% (560 mg). White solid. ^1H NMR (500 MHz, $CDCl_3$) δ 7.95 (d, J = 7.7 Hz, 2 H), 7.87 (s, 1 H), 7.60 (t, J = 7.3 Hz, 1 H), 7.49 (t, J = 7.4 Hz, 2 H), 7.40 (s, 2 H). ^{13}C NMR (125 MHz, $CDCl_3$) δ 165.76, 134.33, 133.44, 133.24, 132.41, 131.24, 128.78, 128.43, 127.66.

Model for Post-Polymer Modification 3ao (Figure 8)

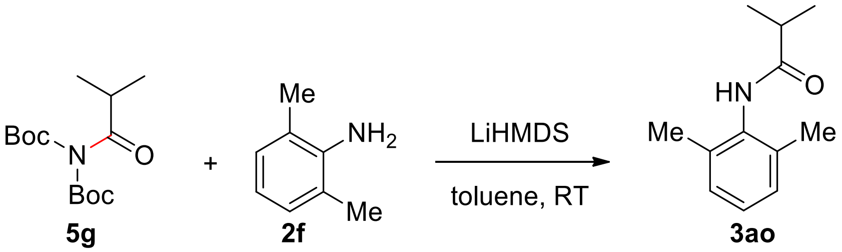

According to the general procedure, an oven-dried vial equipped with a stir bar was charged with of *N*,*N*-Boc_2-isobutyramide (0.10 mmol), 2,6-dimethylaniline (2.0 equiv) placed under a positive pressure of argon, and subjected to three evacuation/backfilling cycles. Toluene (0.25 M) and LiHMDS (1.0 M in THF, 3.0 equiv) were added with vigorous stirring at room temperature, and the reaction mixture was stirred for 15 h at room temperature. After the indicated time, the reaction mixture was quenched with NH_4Cl (aq., 1.0 M, 5 mL), extracted with EtOAc (3 x 10 mL), the organic layers were combined, washed with water (1 x 5 mL), brine (1 x 5 mL), dried over Na_2SO_4 and concentrated. Purification by chromatography on silica gel (EtOAc/hexanes) afforded the title product. Yield 82% (15.7 mg). White solid. ^{1}H NMR (500 MHz, $CDCl_3$) δ 7.16-7.06 (m, 3 H), 6.80 (s, 1 H), 2.63 (dt, *J* = 13.8, 6.9 Hz, 1 H), 2.22 (s, 6 H), 1.31 (d, *J* = 6.9 Hz, 6 H). ^{13}C NMR (125 MHz, $CDCl_3$) δ 175.21, 135.47, 133.79, 128.15, 127.18, 35.94, 19.90, 18.34. *Note:* analogous reaction using aniline (2.0 equiv) instead of 2,6-dimethylaniline, afforded the transamidation product in 90% yield, demonstrating that both sterically-hindered and sterically-non-hindered, non-nucleophilic amines are competent nucleophiles in the reaction.

Synthesis of Moclobemide 3ap (Figure 9)

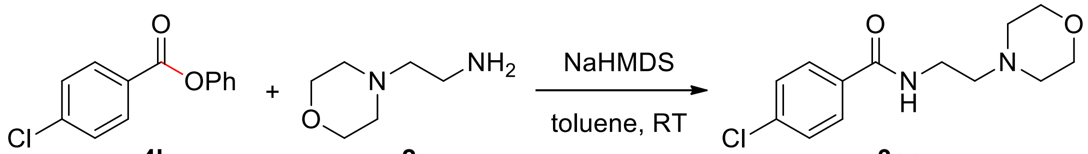

According to the general procedure, an oven-dried vial equipped with a stir bar was charged with phenyl 4-chlorobenzoate (0.10 mmol), 2-morpholinoethanamine (1.0 equiv) placed under a positive pressure of argon, and subjected to three evacuation/backfilling cycles. Toluene (0.25 M) and NaHMDS (1.0 M in THF, 2.0 equiv) were added with vigorous stirring at room temperature, and the reaction mixture was stirred for 15 h at room temperature. After the indicated time, the reaction mixture was quenched with NH_4Cl (aq., 1.0 M, 5 mL), extracted with EtOAc (3 x 10 mL), the organic layers were combined, washed with water (1 x 5 mL), brine (1 x 5 mL), dried over Na_2SO_4 and concentrated. Purification by chromatography on silica gel (EtOAc/hexanes) afforded the title product. Yield 88% (23.7 mg). White solid. ^{1}H NMR (500 MHz, $CDCl_3$) δ 7.79 (d, J = 8.1 Hz, 2 H), 7.44 (d, J = 8.2 Hz, 2 H), 7.11 (s, 1 H), 3.84-3.76 (m, 4 H), 3.63 (dd, J = 10.8, 5.3 Hz, 2 H), 2.73 (t, J = 5.4 Hz, 2 H), 2.65 (s, 4 H). ^{13}C NMR (125 MHz, $CDCl_3$) δ 166.40, 137.76, 132.71, 128.84, 128.50, 66.43, 57.16, 53.29, 35.74.

Synthesis of Lidocaine 3aq (Figure 9)

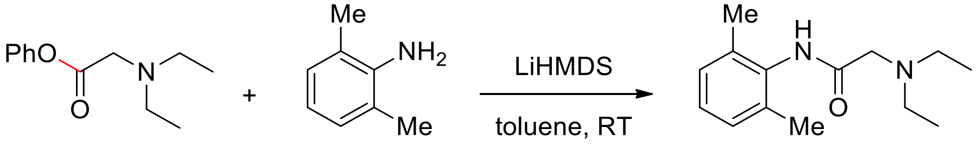

4l **2f** **3aq**

According to the general procedure, an oven-dried vial equipped with a stir bar was charged with phenyl diethylglycinate (0.10 mmol), 2,6-dimethylaniline (1.0 equiv) placed under a positive pressure of argon, and subjected to three evacuation/backfilling cycles. Toluene (0.25 M) and LiHMDS (1.0 M in THF, 2.0 equiv) were added with vigorous stirring at room temperature, and the reaction mixture was stirred for 15 h at room temperature. After the indicated time, the reaction mixture was quenched with NH_4Cl (aq., 1.0 M, 5 mL), extracted with EtOAc (3 x 10 mL), the organic layers were combined, washed with water (1 x 5 mL), brine (1 x 5 mL), dried over Na_2SO_4 and concentrated. Purification by chromatography on silica gel (EtOAc/hexanes) afforded the title product. Yield 91% (21.3 mg). White solid.^{1}H NMR (500 MHz, $CDCl_3$) δ 8.94 (s, 1 H), 7.11 (s, 3 H), 3.25 (s, 2 H), 2.72 (q, J = 7.1 Hz, 4 H), 2.26 (s, 6 H), 1.16 (t, J = 7.1 Hz, 6 H). ^{13}C NMR (125 MHz, $CDCl_3$) δ 170.29, 135.10, 133.99, 128.24, 127.08, 57.55, 48.97, 18.59, 12.68.

HPLC Traces

Compound **3am**: HPLC analysis (chiralcel OD-H, *n*-hexane/*i*-PrOH 90/10, 1.0 mL/min, 254 nm), 97.5% ee: t_R (major) = 6.14 minutes, t_R (minor) = 7.27 minutes.

Supplementary Figure 5. HPLC trace of (*R*)-3am

(*R*)-**3am**:

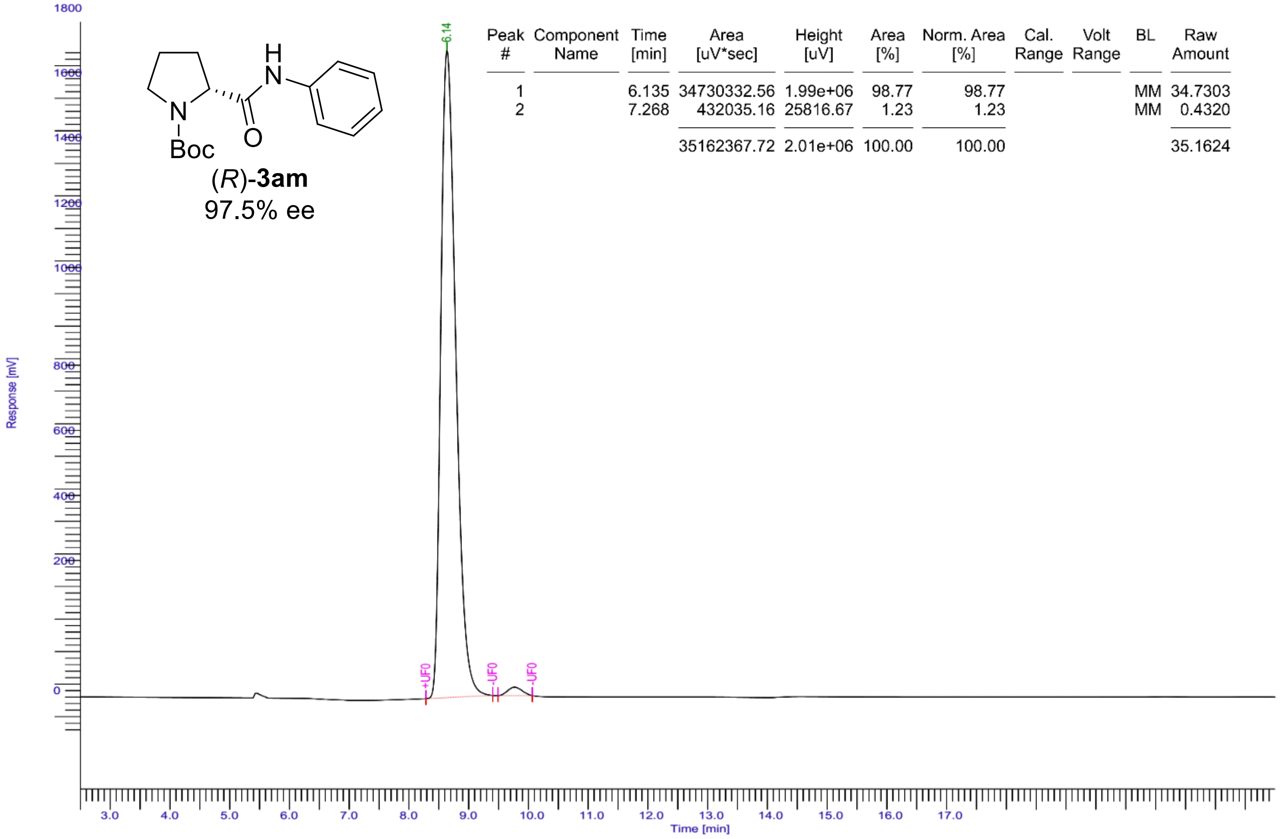

Supplementary Figure 6. HPLC trace of (*rac*)-3am

(*rac*)-**3am**:

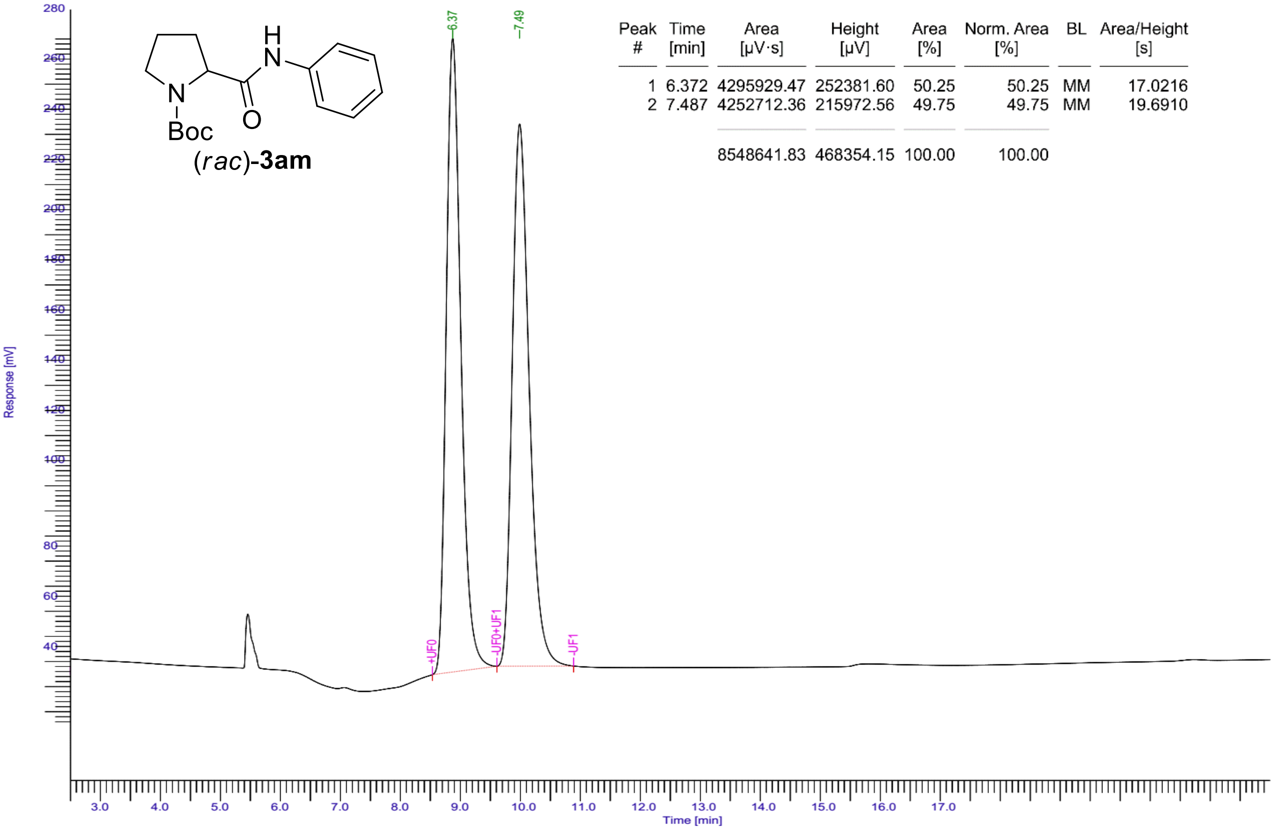

Peak #	Time [min]	Area [μV·s]	Height [μV]	Area [%]	Norm. Area [%]	BL	Area/Height [s]
1	6.372	4295929.47	252381.60	50.25	50.25	MM	17.0216
2	7.487	4252712.36	215972.56	49.75	49.75	MM	19.6910
		8548641.83	468354.15	100.00	100.00		

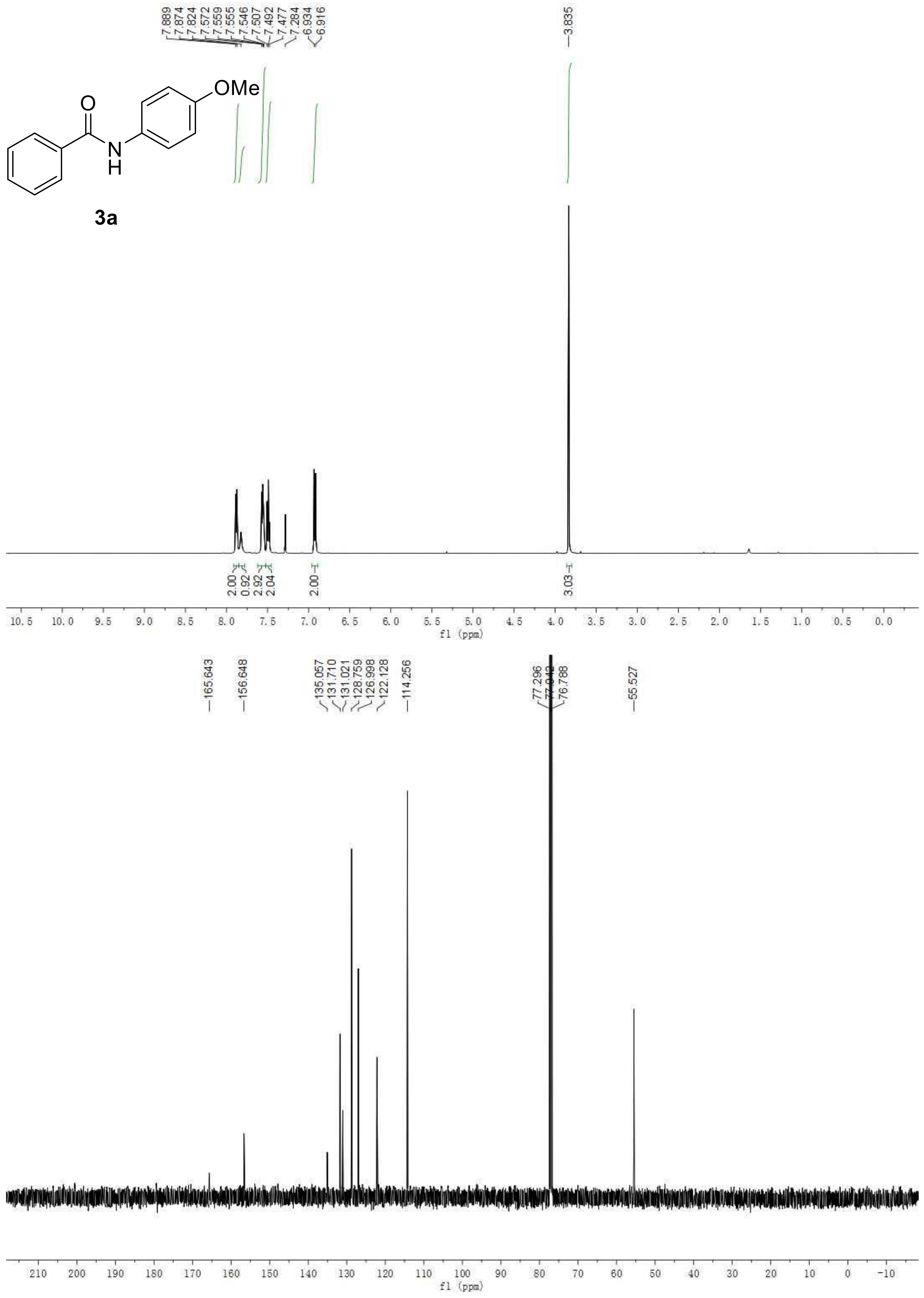

Supplementary Figure 7. ^{1}H and ^{13}C NMR spectra of 3a

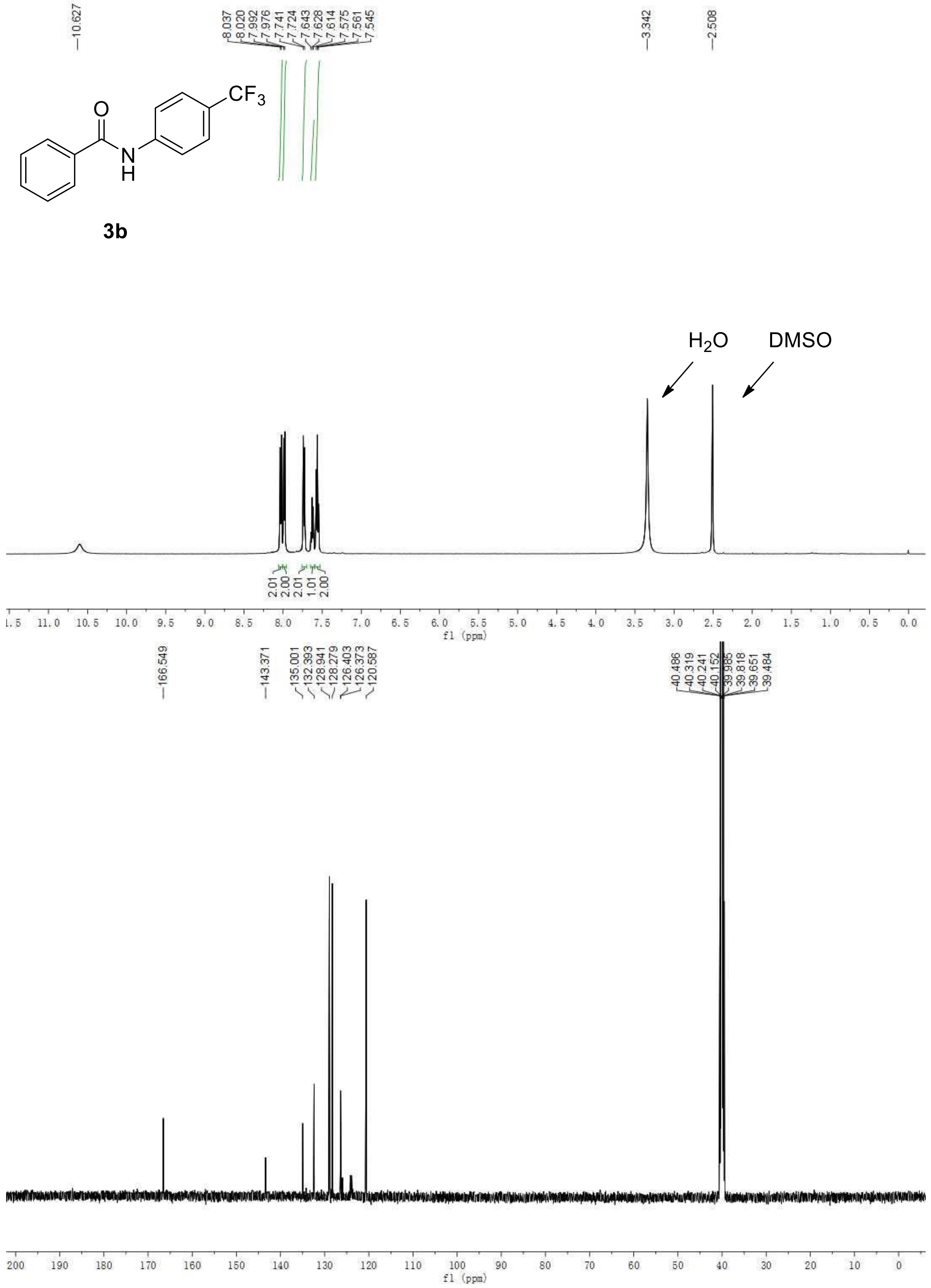

Supplementary Figure 8. ^{1}H and ^{13}C NMR spectra of 3b

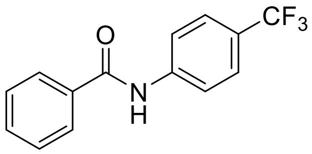

3b

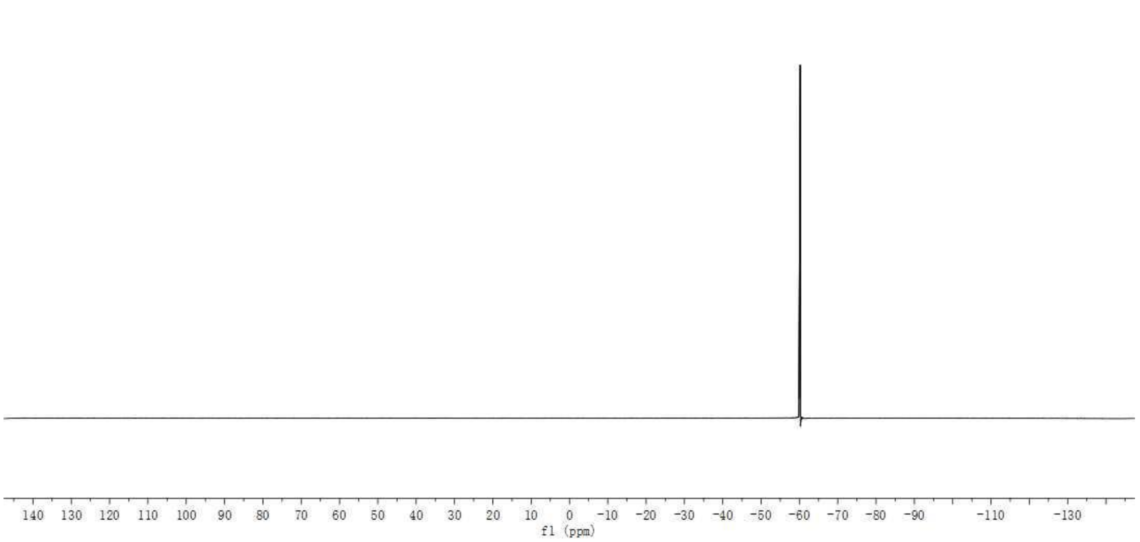

Supplementary Figure 9. ^{19}F NMR spectrum of 3b

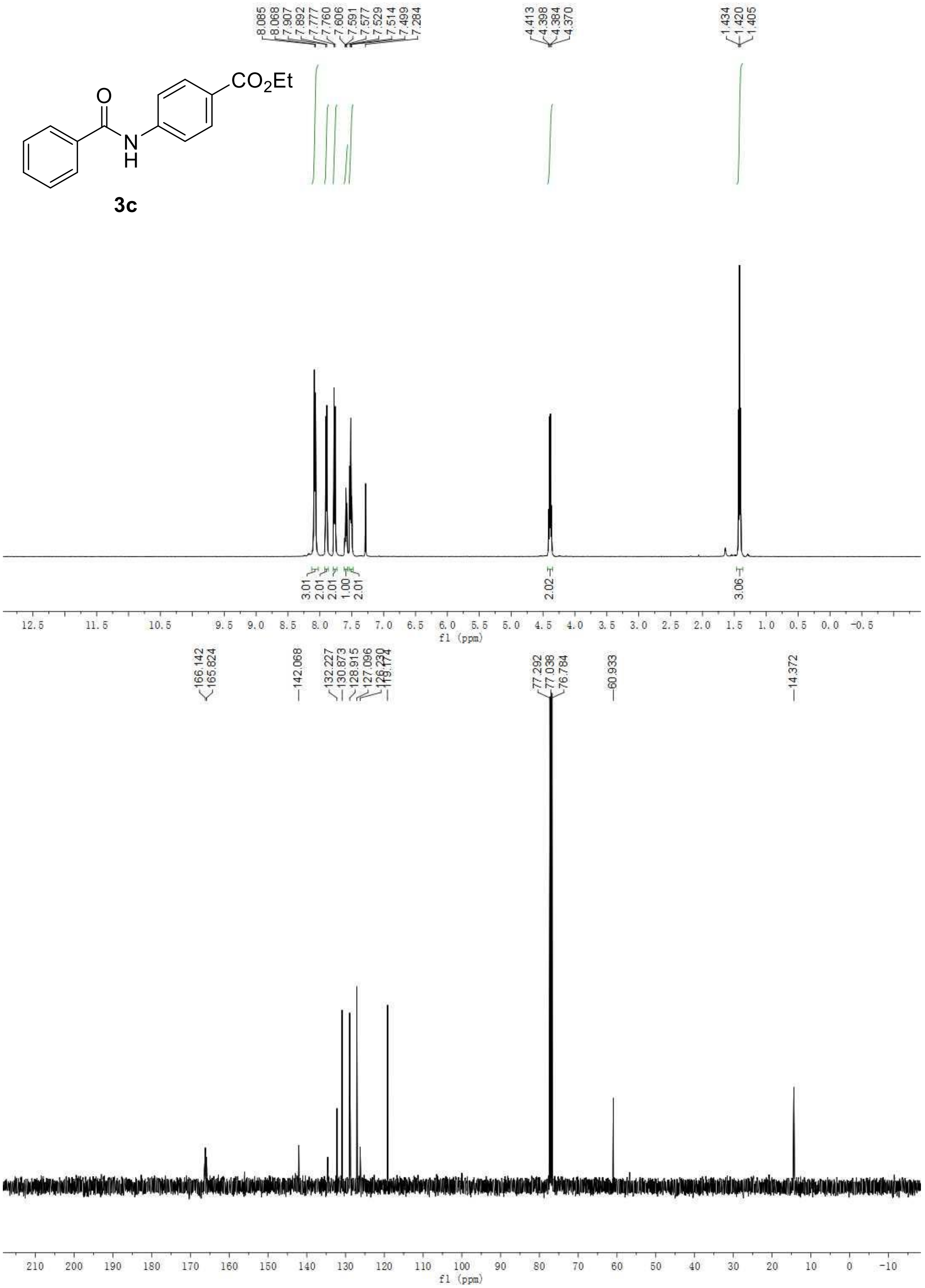

Supplementary Figure 10. ^{1}H and ^{13}C NMR spectra of 3c

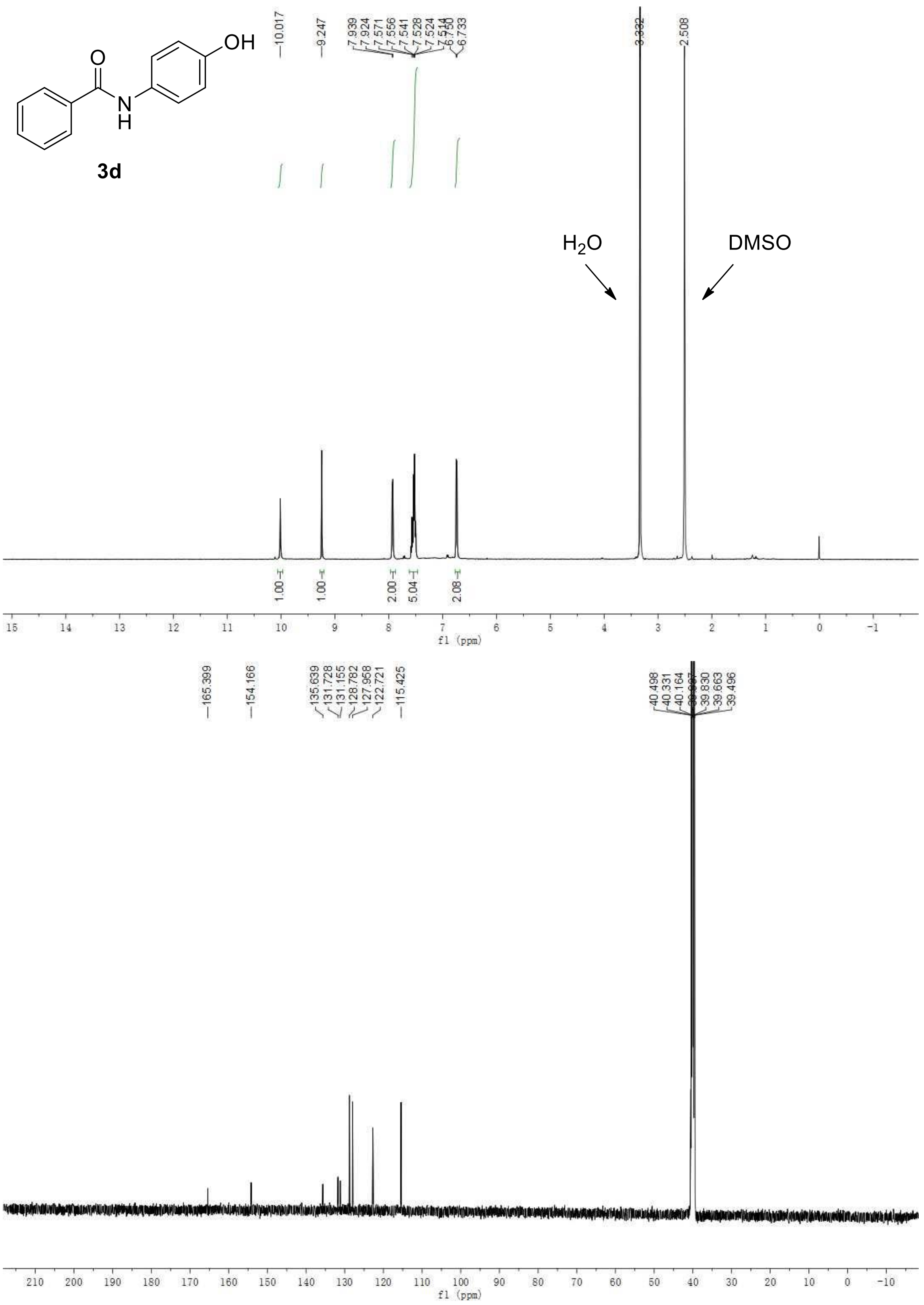

Supplementary Figure 11. ^{1}H and ^{13}C NMR spectra of 3d

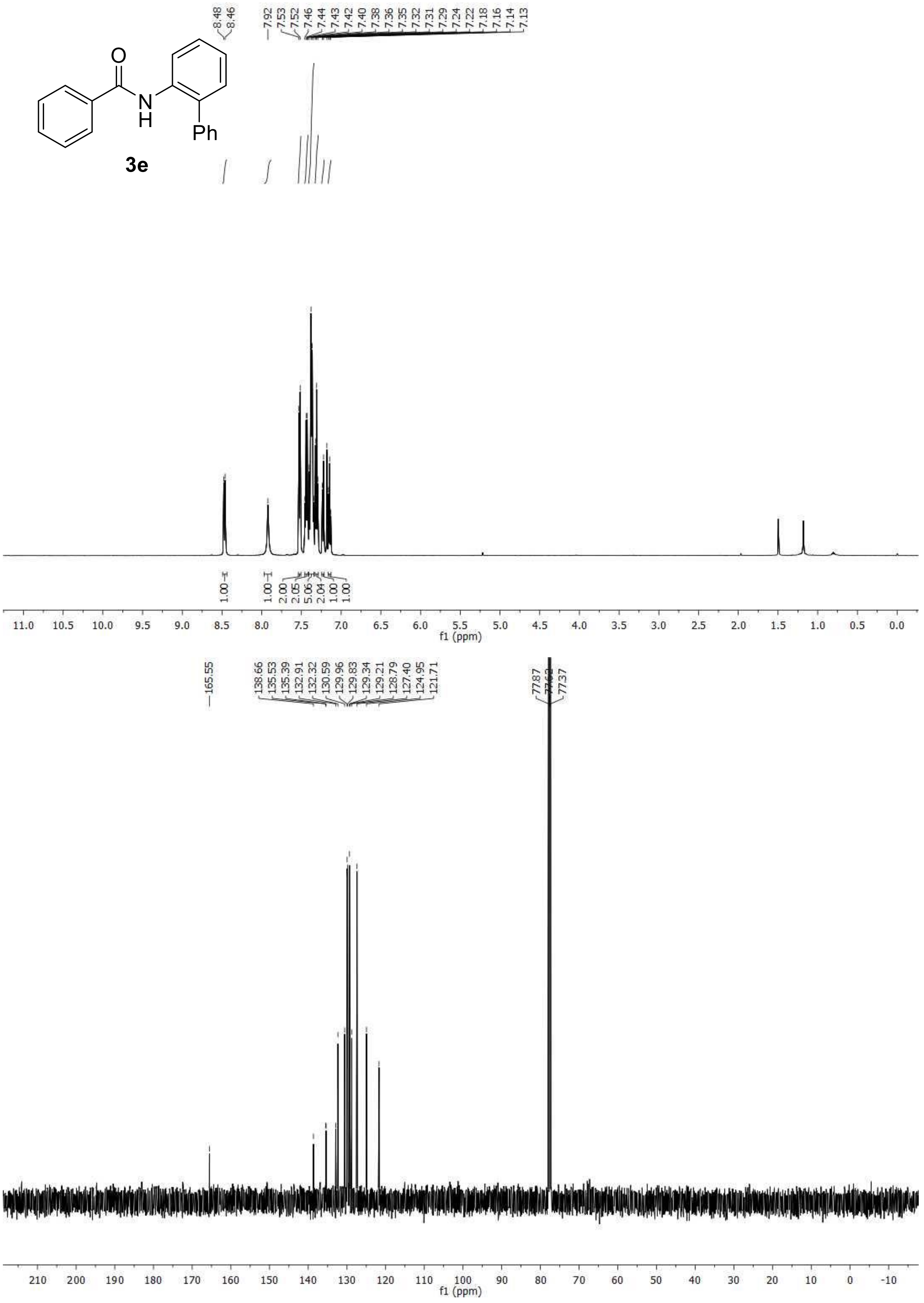

Supplementary Figure 12. ^{1}H and ^{13}C NMR spectra of 3e

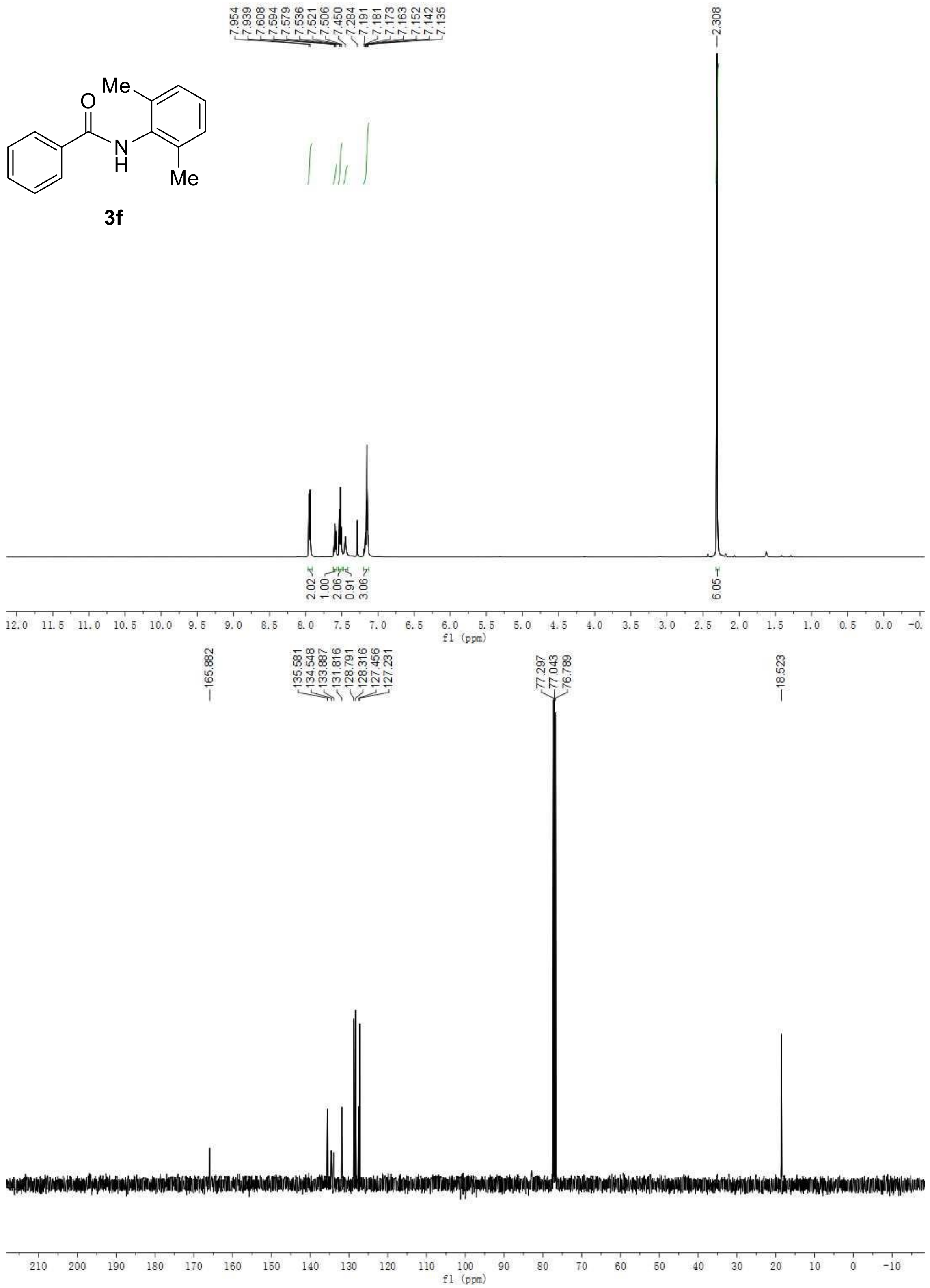

Supplementary Figure 13. ^{1}H and ^{13}C NMR spectra of 3f

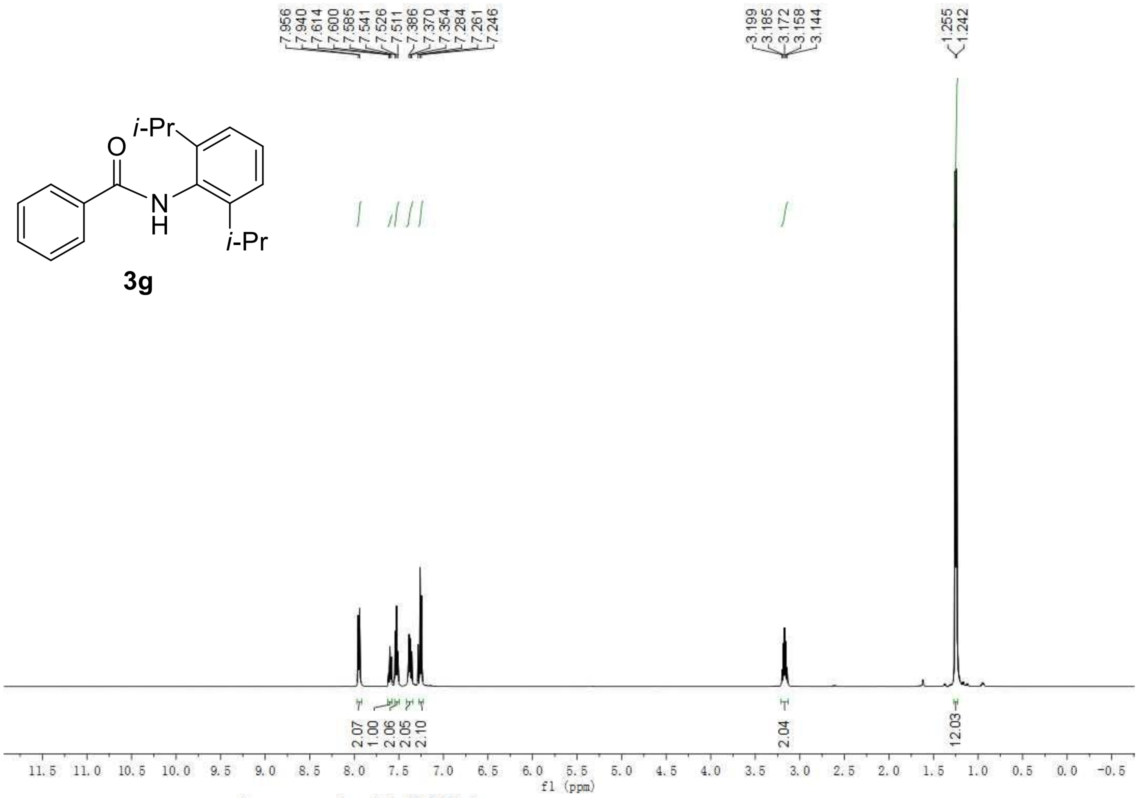

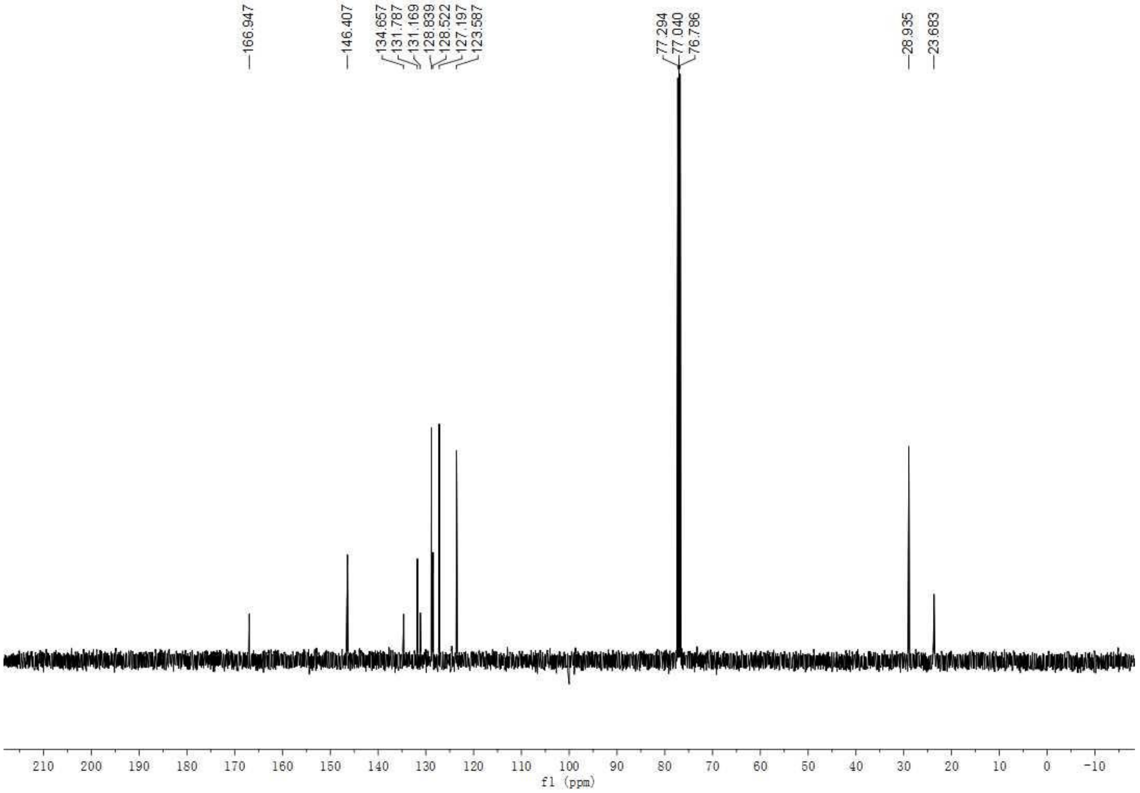

Supplementary Figure 14. ^{1}H and ^{13}C NMR spectra of 3g

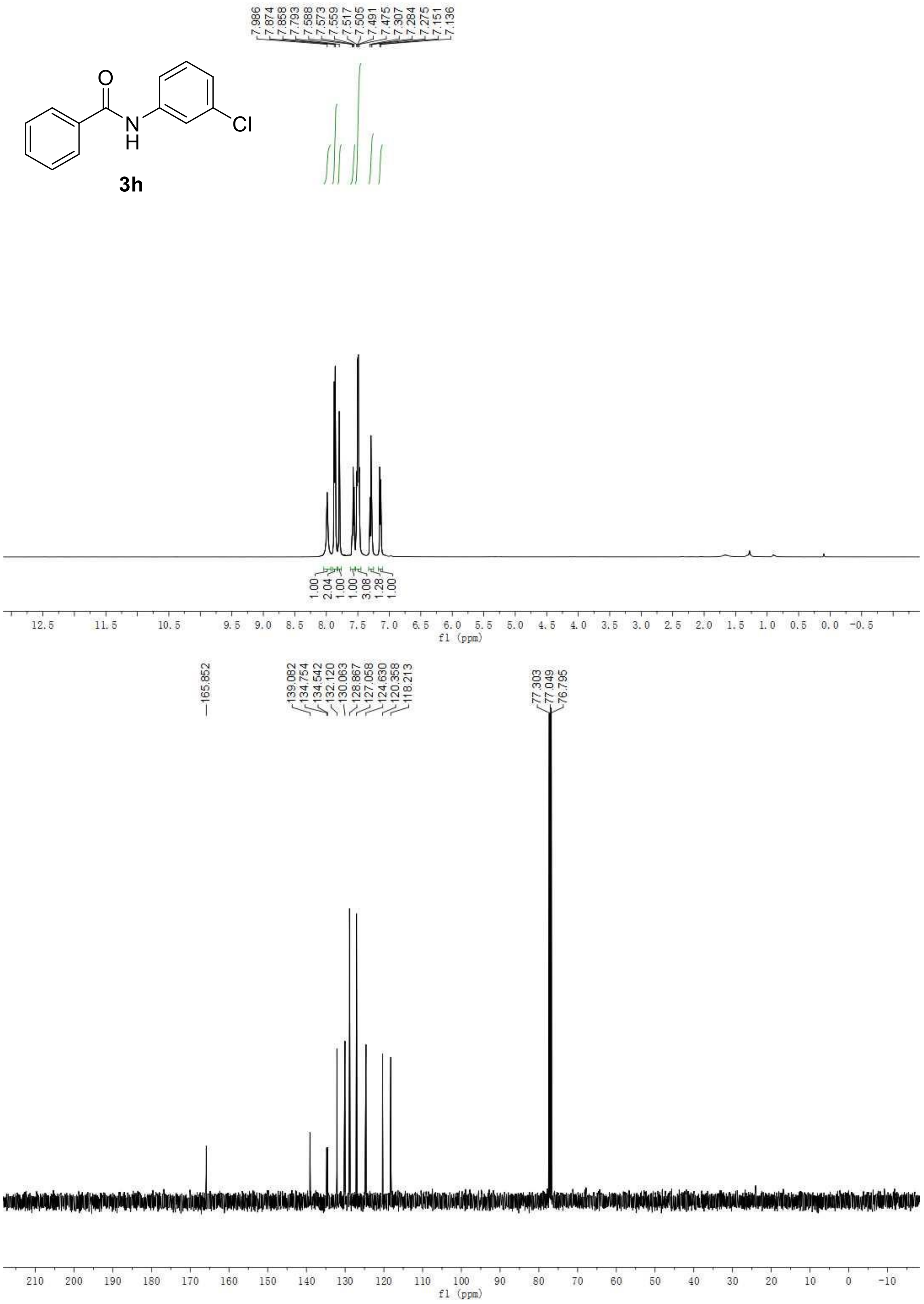

Supplementary Figure 15. ^{1}H and ^{13}C NMR spectra of 3h

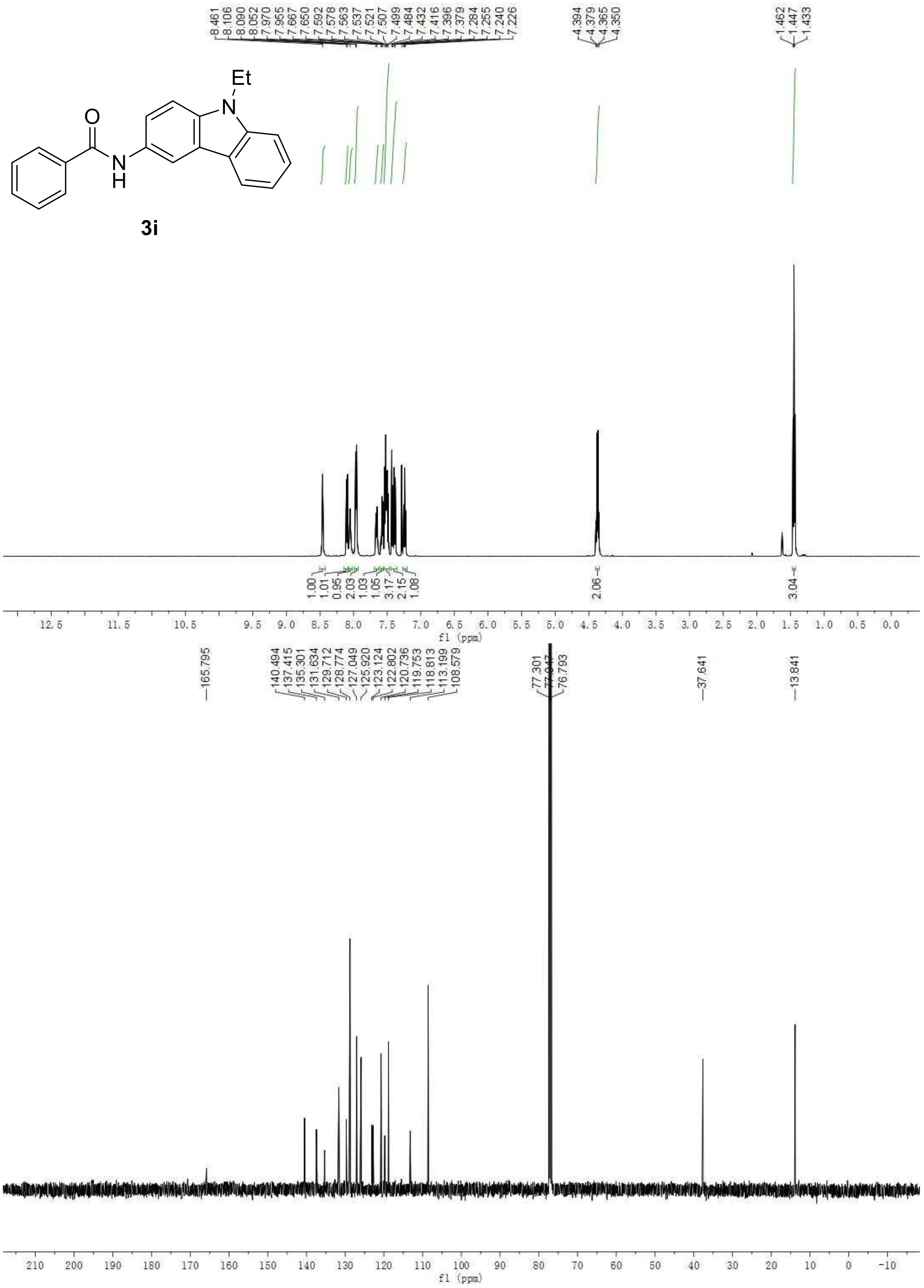

Supplementary Figure 16. ^{1}H and ^{13}C NMR spectra of 3i

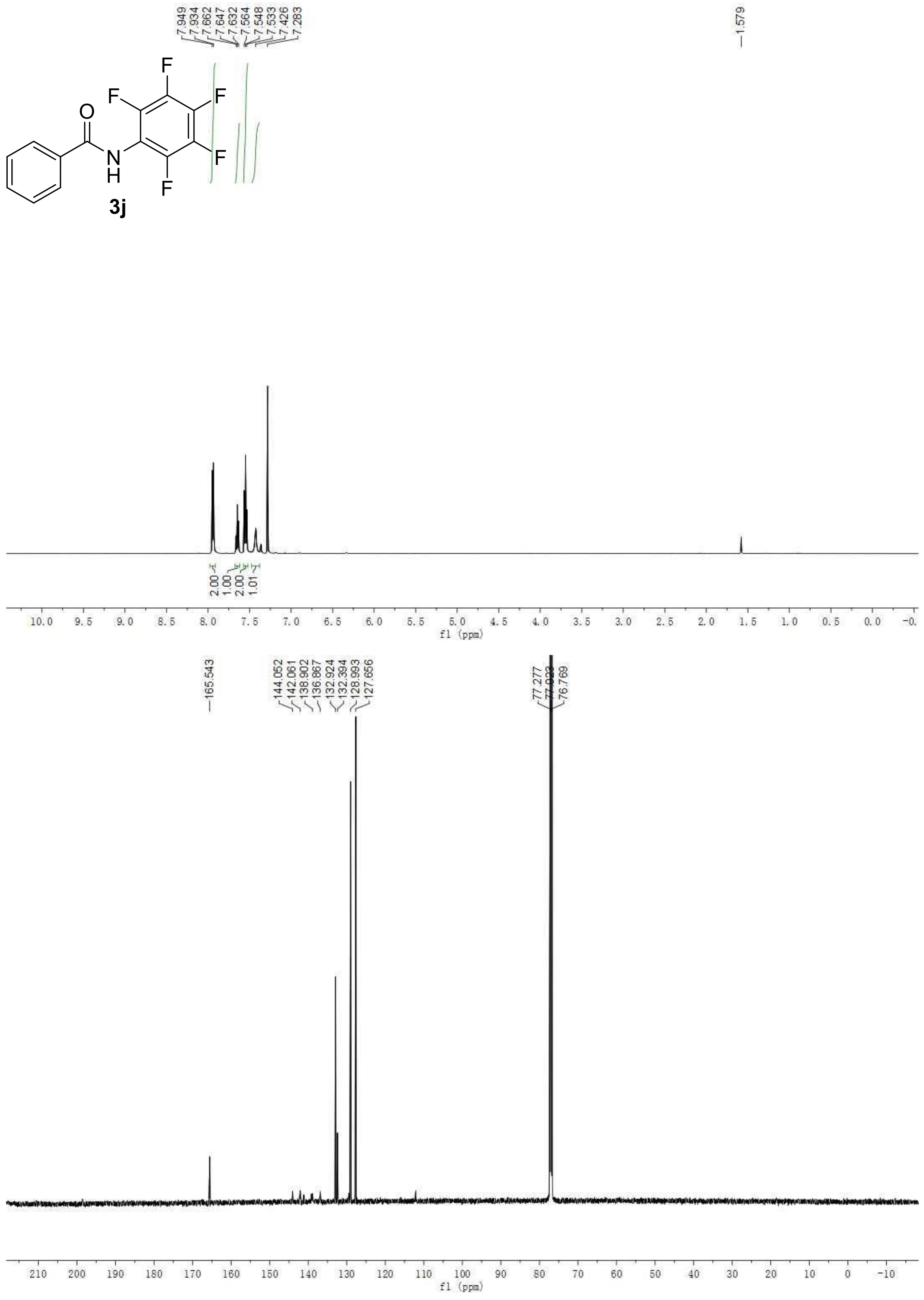

Supplementary Figure 17. ^{1}H and ^{13}C NMR spectra of 3j

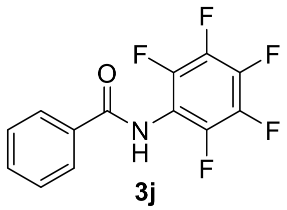

3j

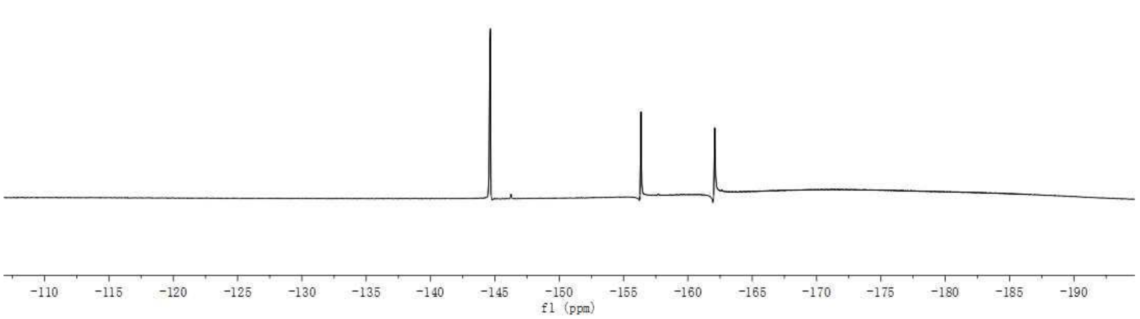

Supplementary Figure 18. ^{19}F NMR spectrum of 3j

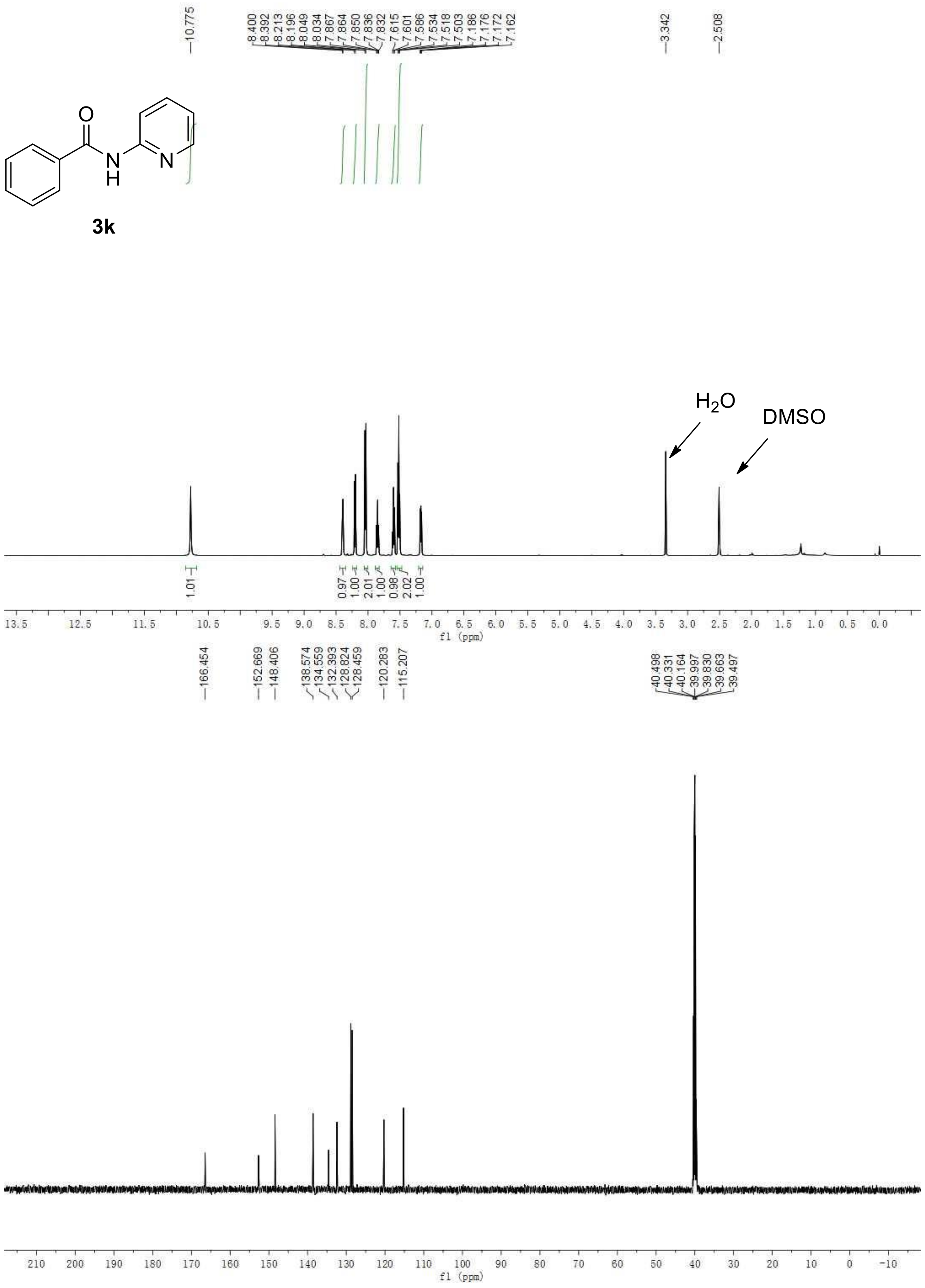

Supplementary Figure 19. ^{1}H and ^{13}C NMR spectra of 3k

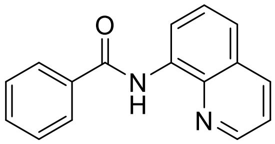

3l

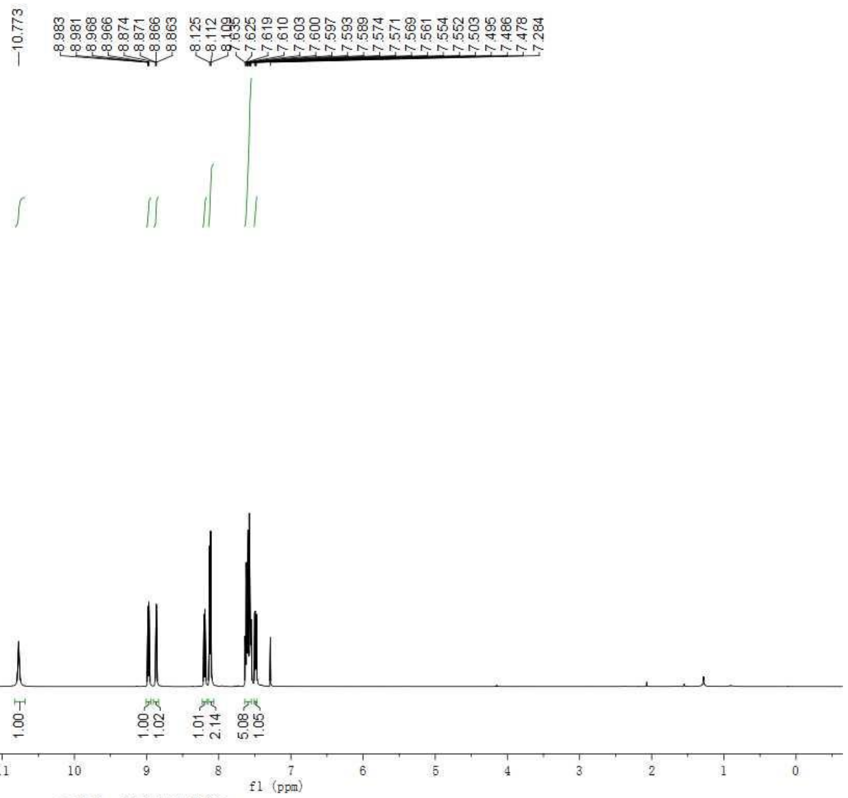

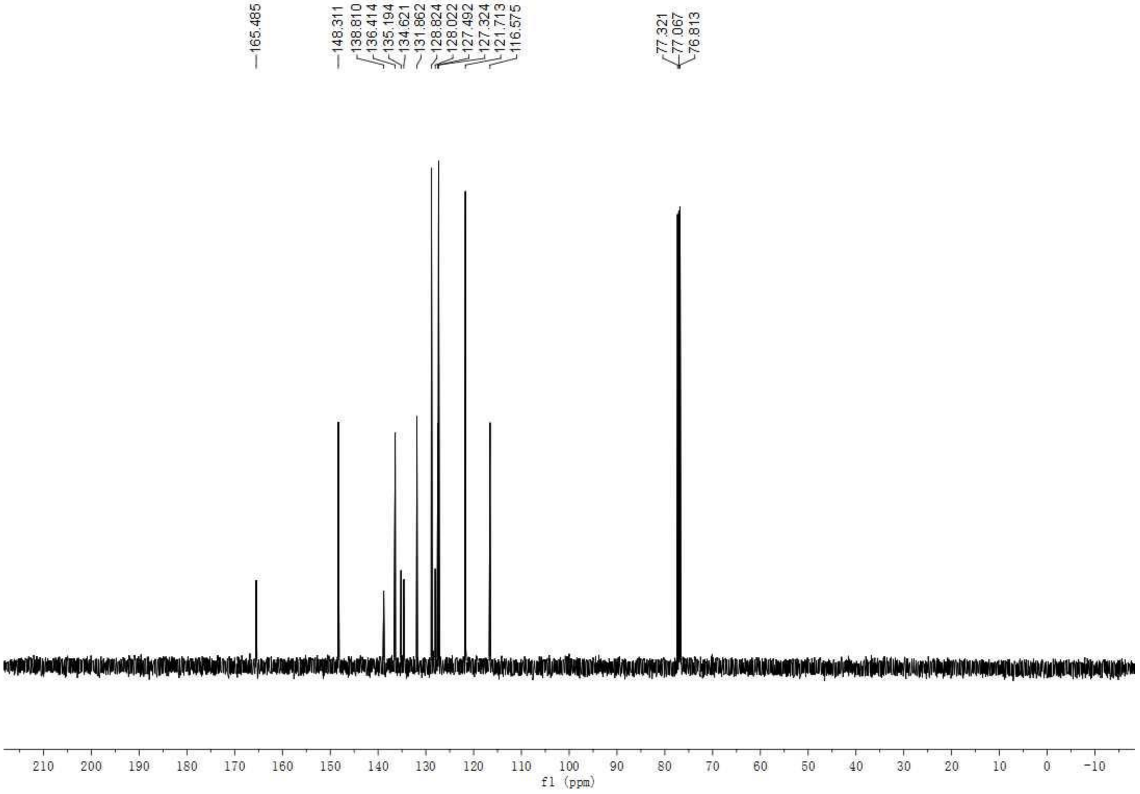

Supplementary Figure 20. ¹H and ¹³C NMR spectra of 3l

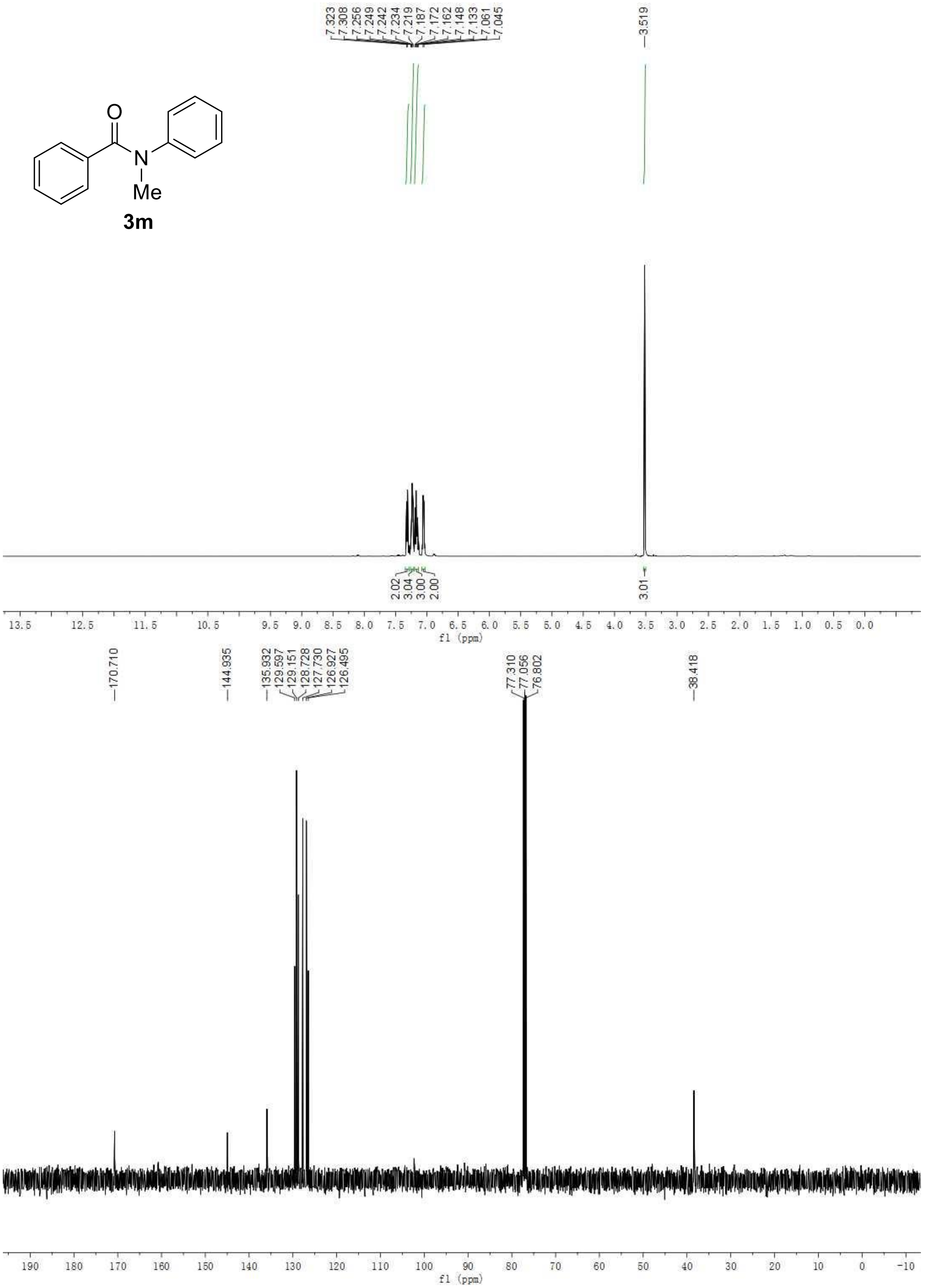

Supplementary Figure 21. ^{1}H and ^{13}C NMR spectra of 3m

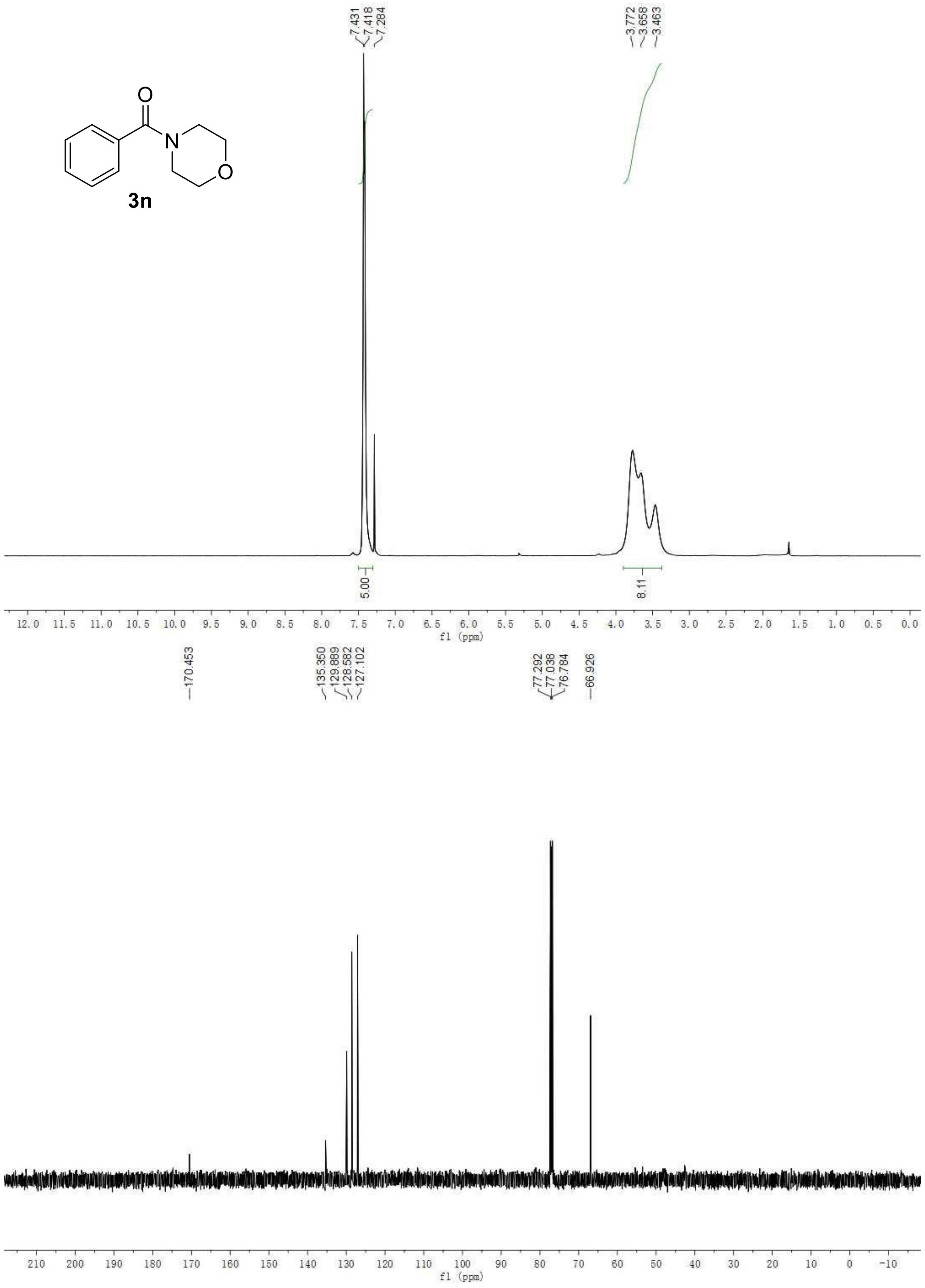

Supplementary Figure 22. ^{1}**H and** ^{13}**C NMR spectra of 3n**

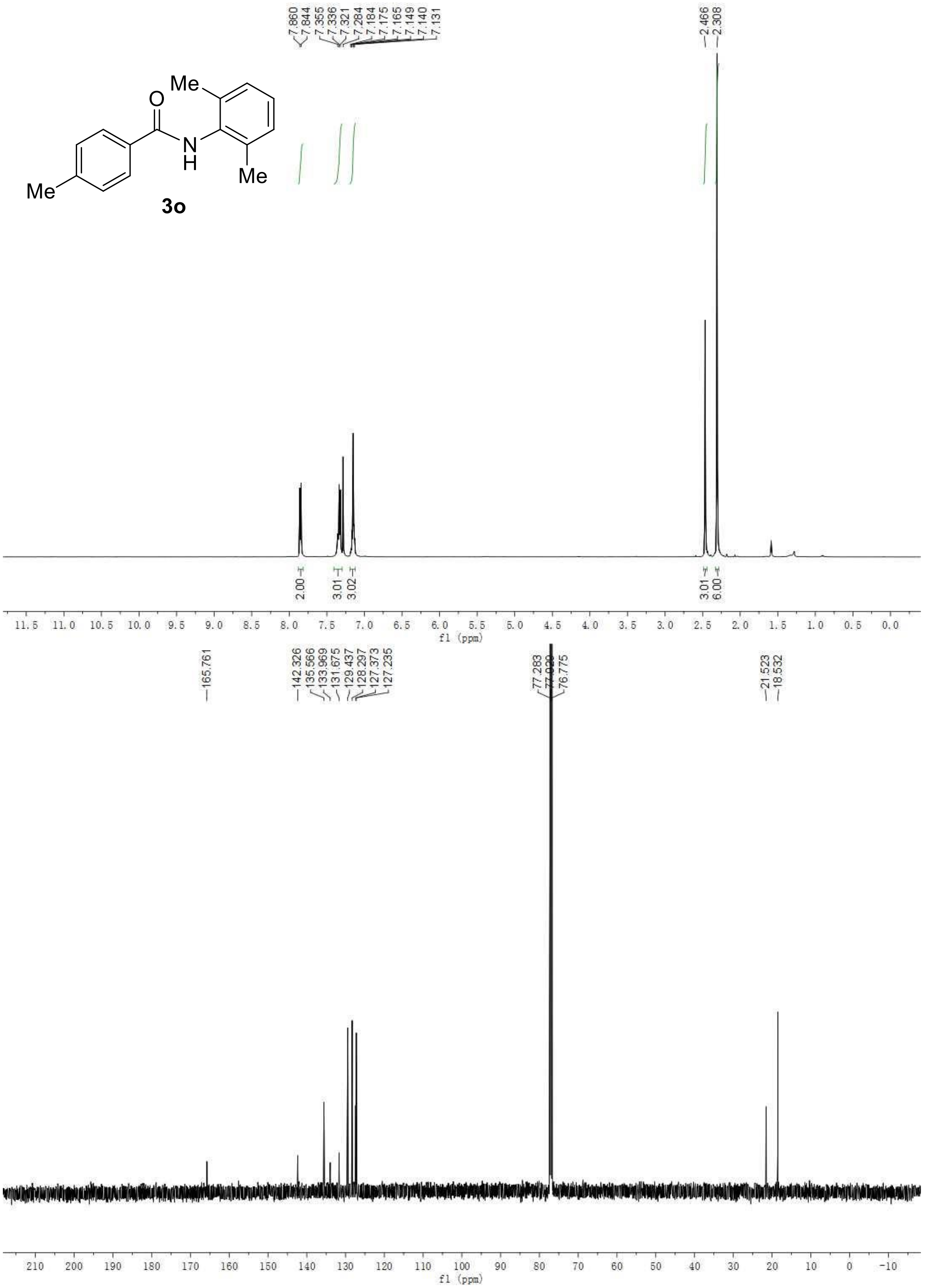

Supplementary Figure 23. ^{1}**H and** ^{13}**C NMR spectra of 3o**

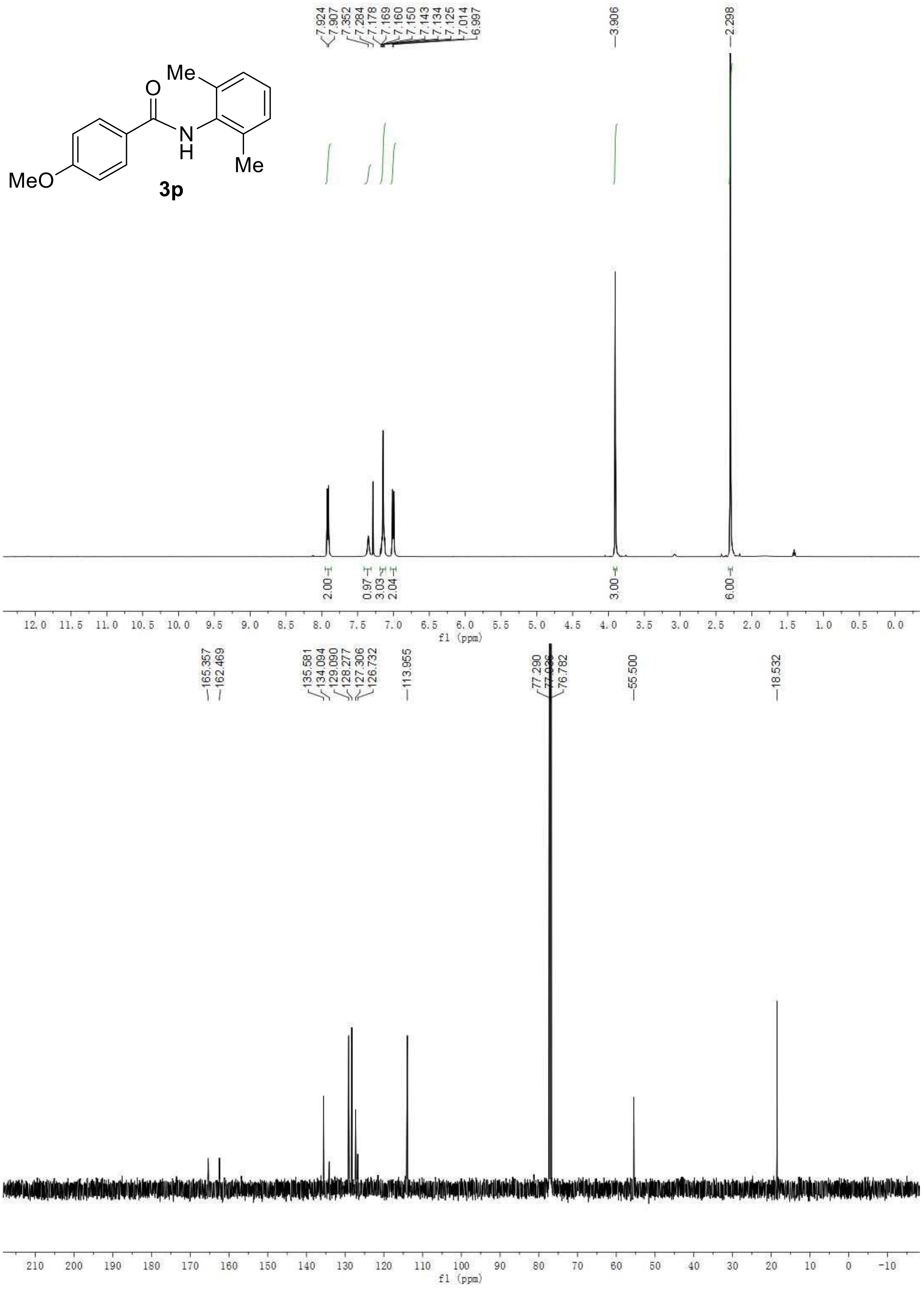

Supplementary Figure 24. ^{1}H and ^{13}C NMR spectra of 3p

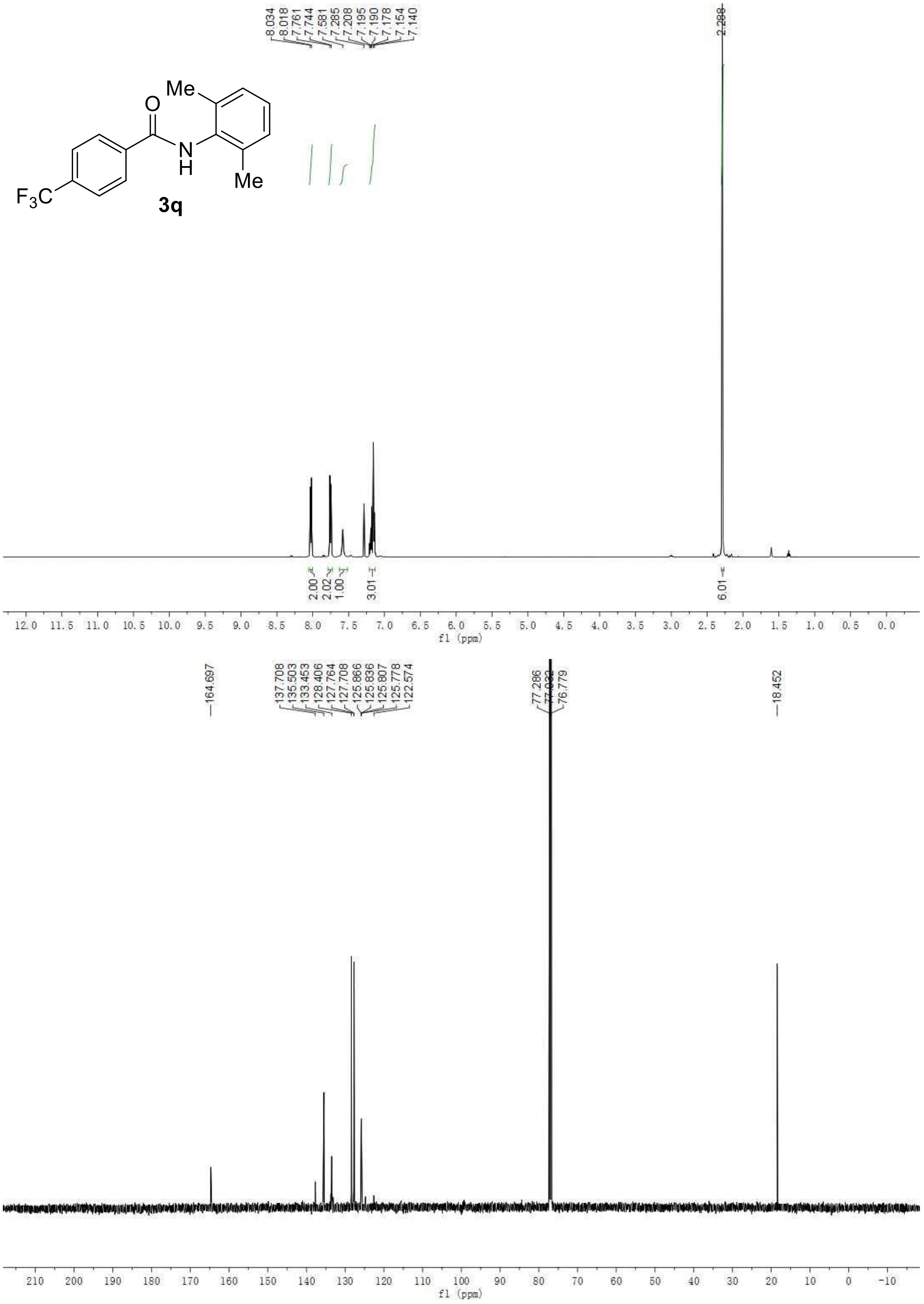

Supplementary Figure 25. ^{1}H and ^{13}C NMR spectra of 3q

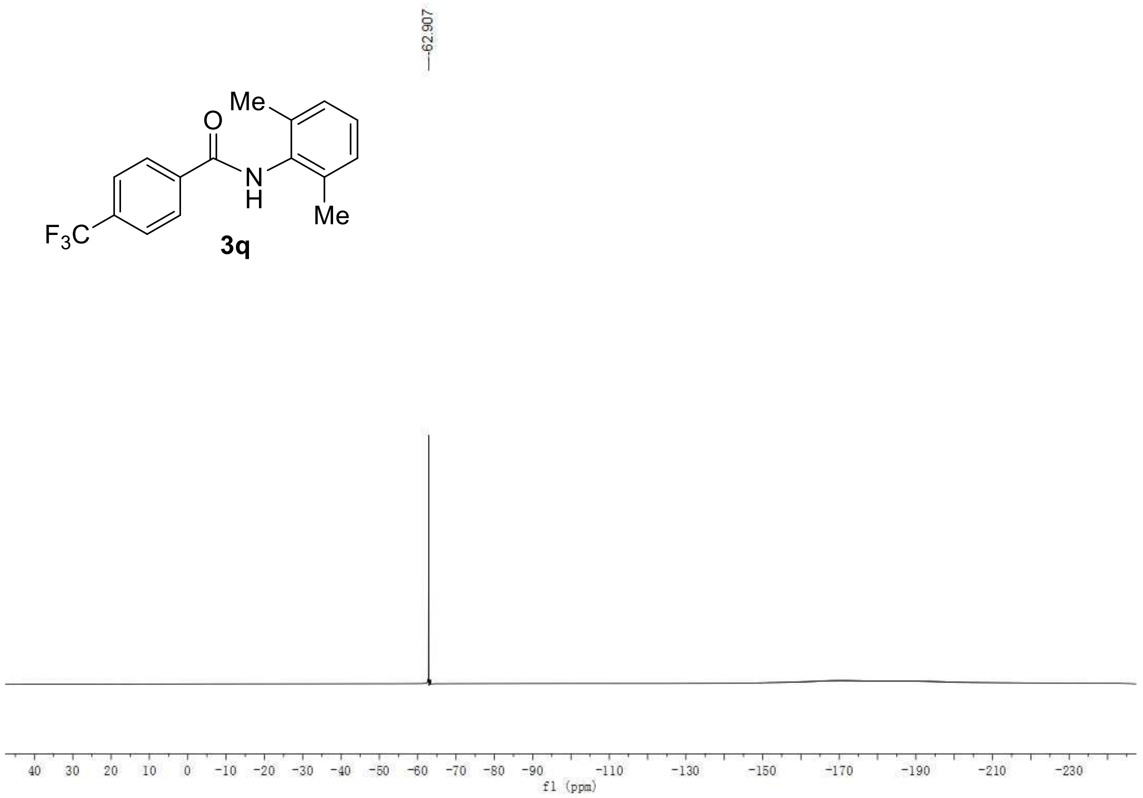

Supplementary Figure 26. ^{19}F NMR spectra of 3q

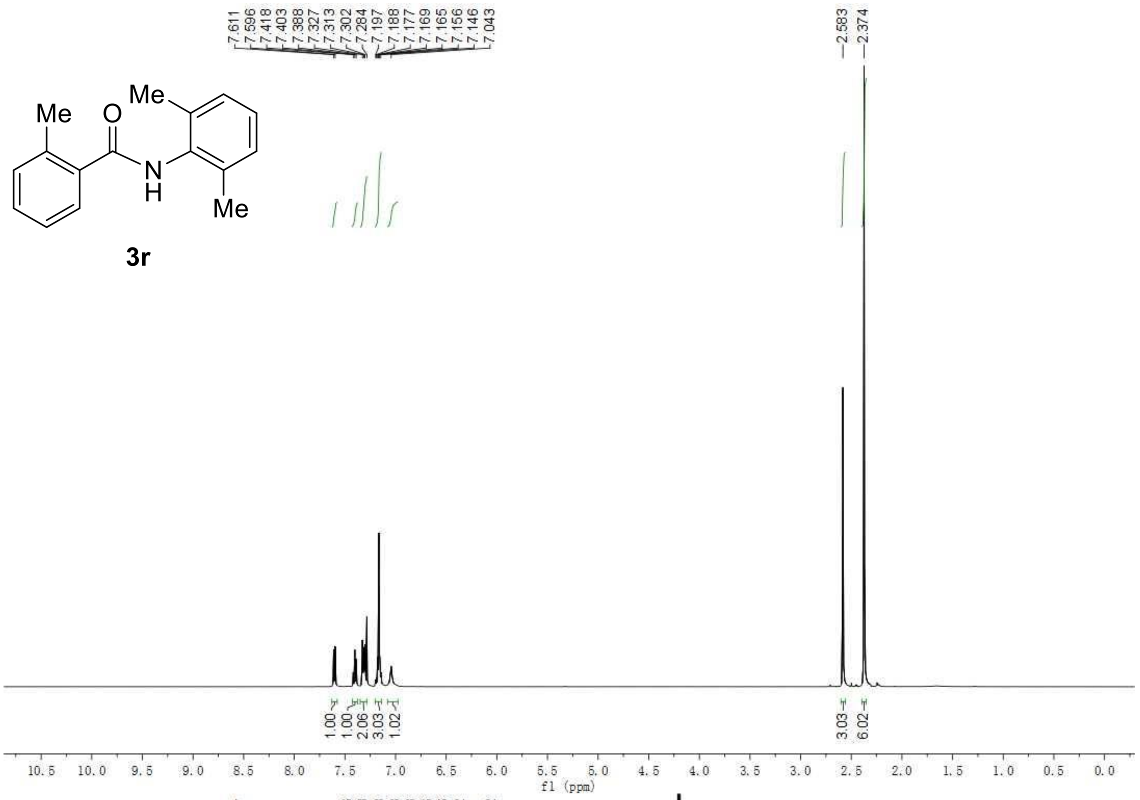

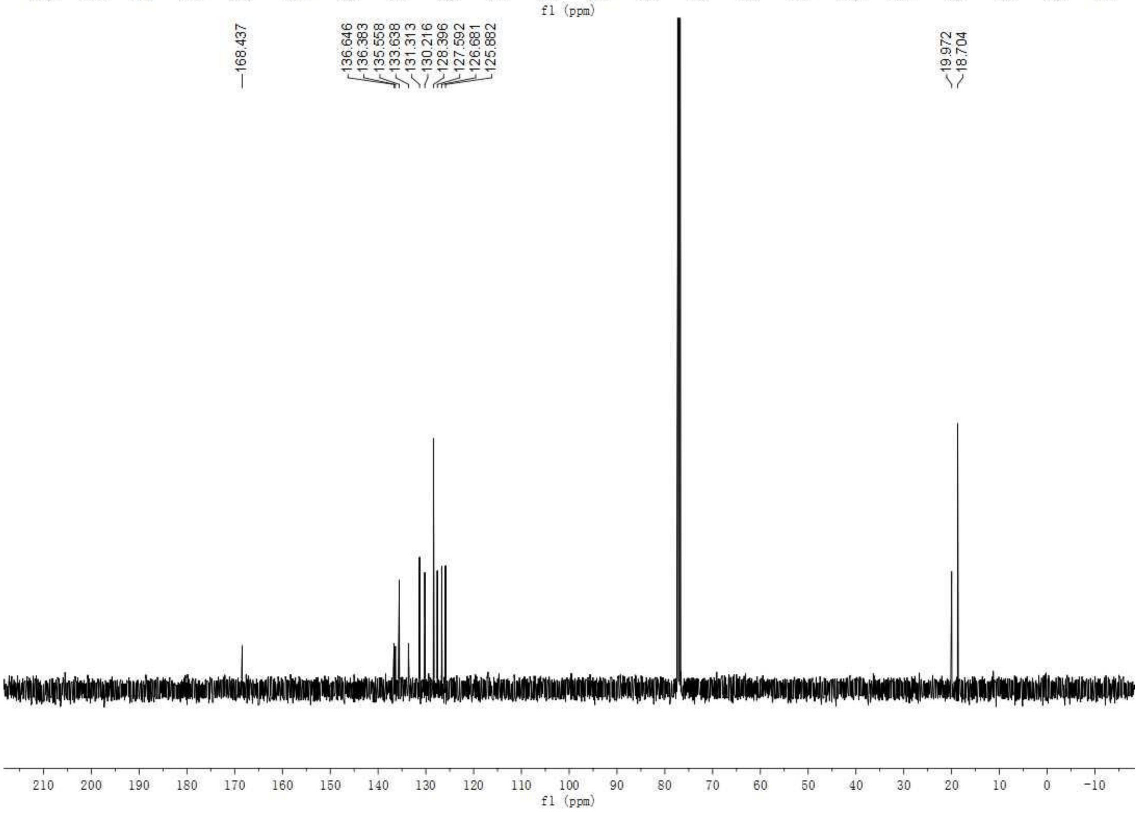

Supplementary Figure 27. ^{1}**H and** ^{13}**C NMR spectra of 3r**

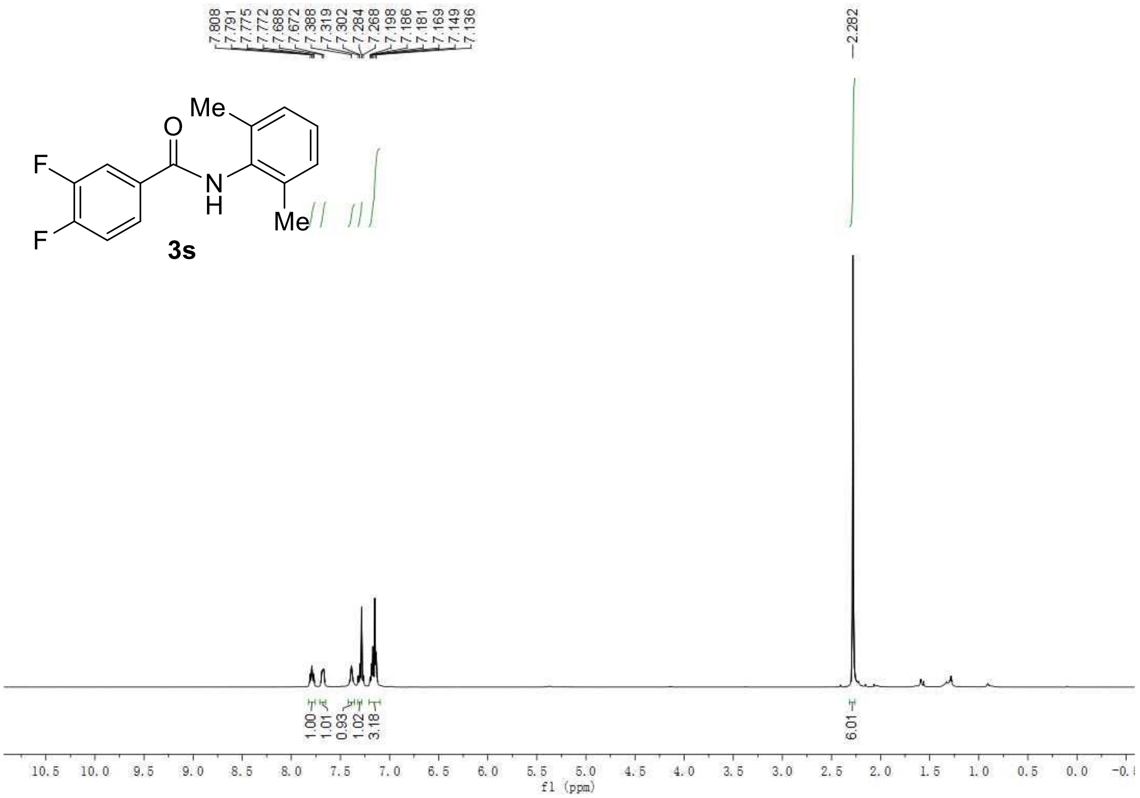

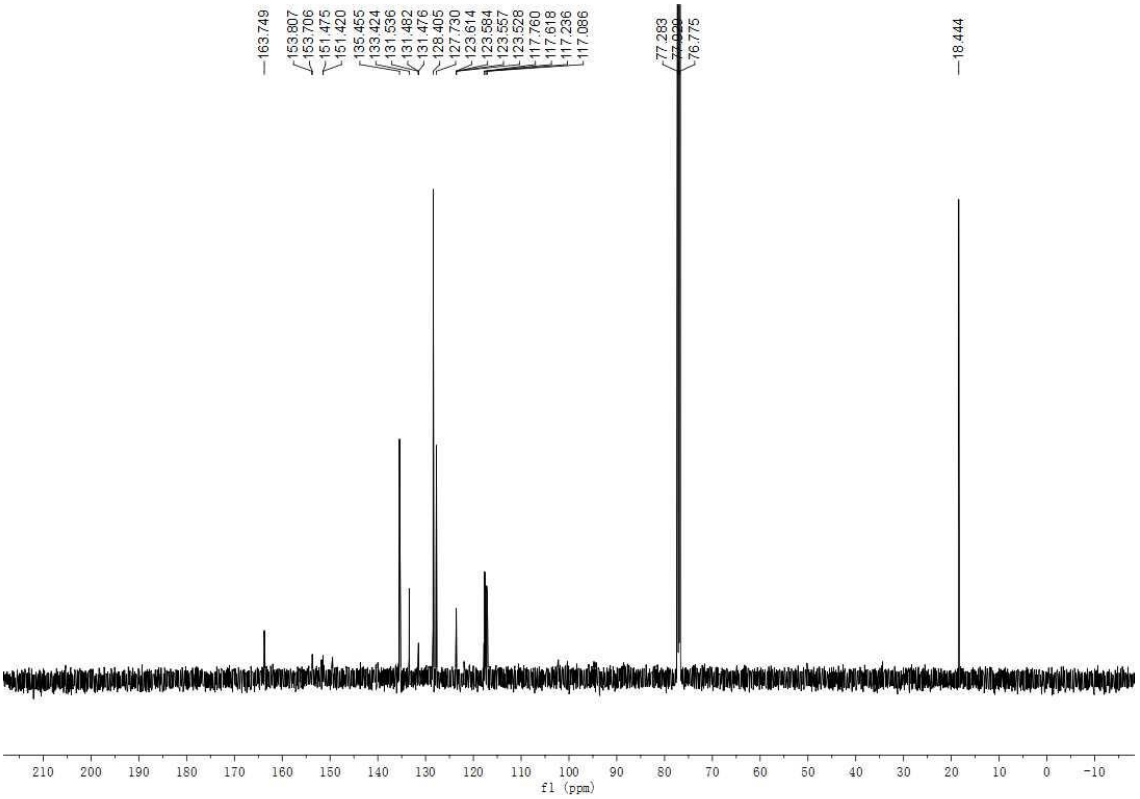

Supplementary Figure 28. ^{1}H and ^{13}C NMR spectra of 3s

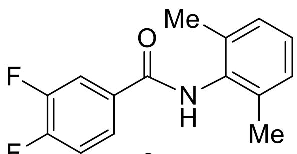

3s

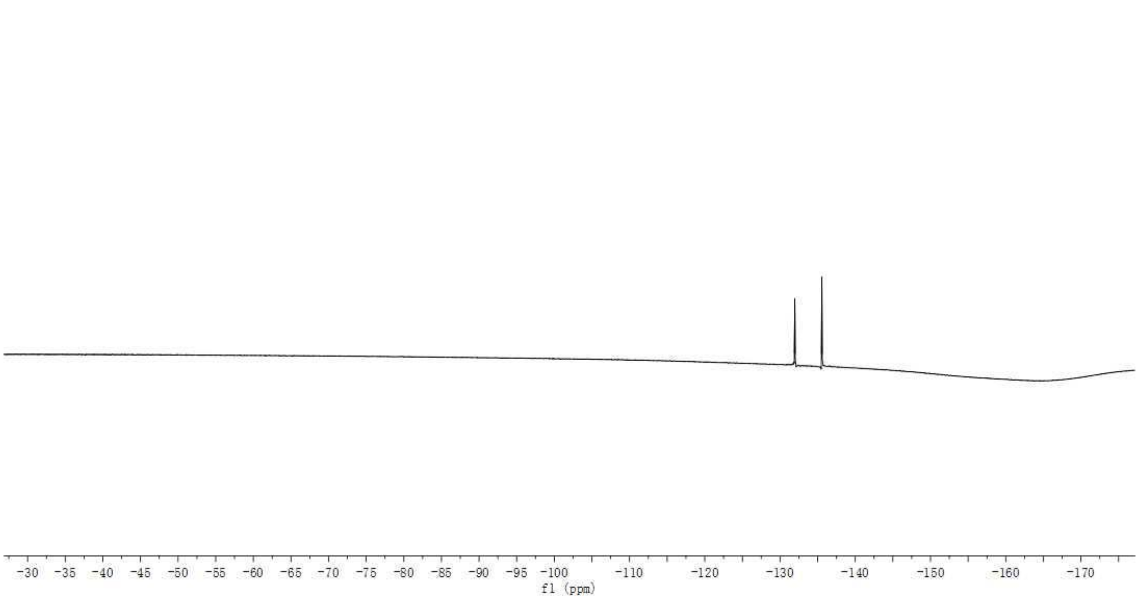

Supplementary Figure 29. ^{19}F NMR spectrum of 3s

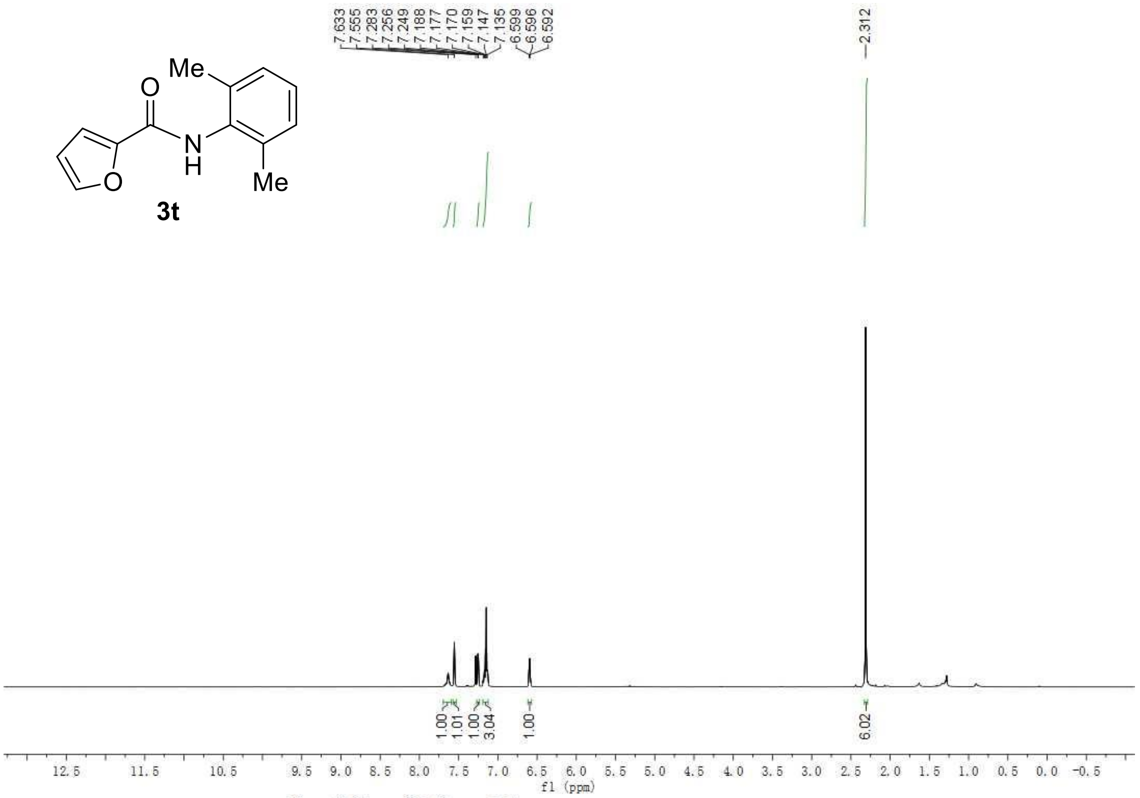
3t

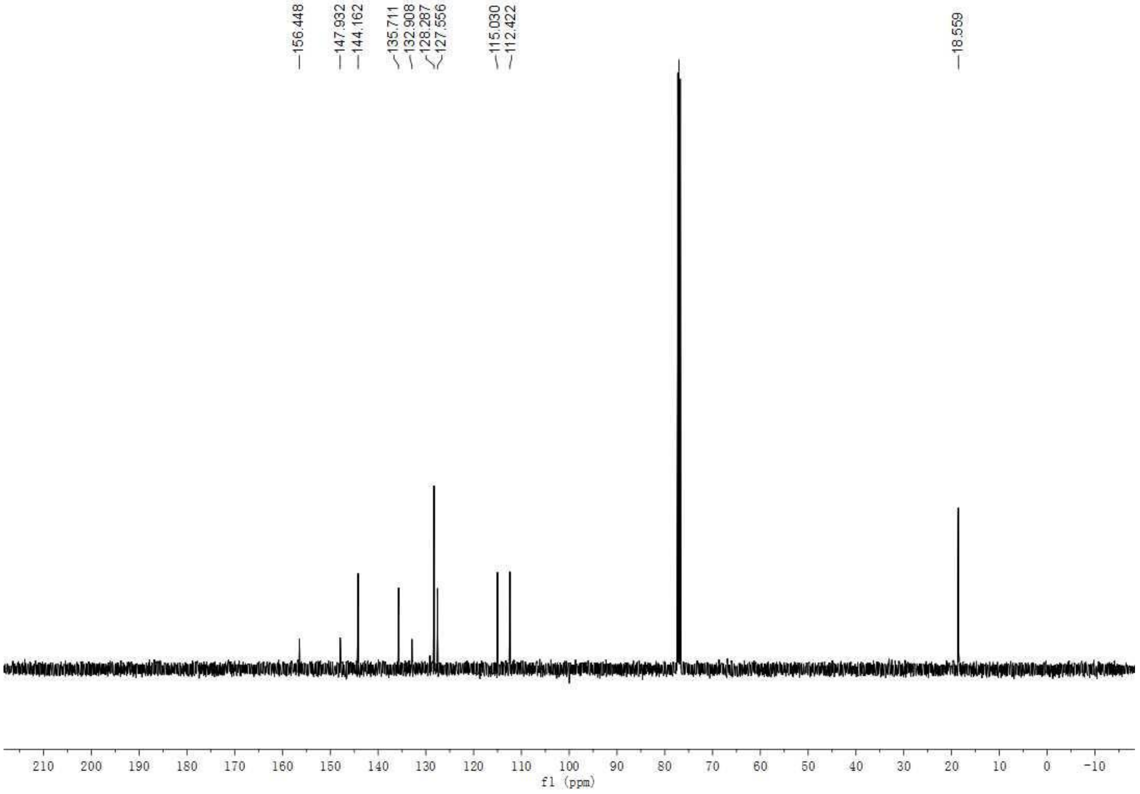

Supplementary Figure 30. ^{1}H and ^{13}C NMR spectra of 3t

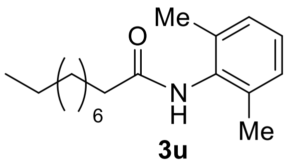
3u

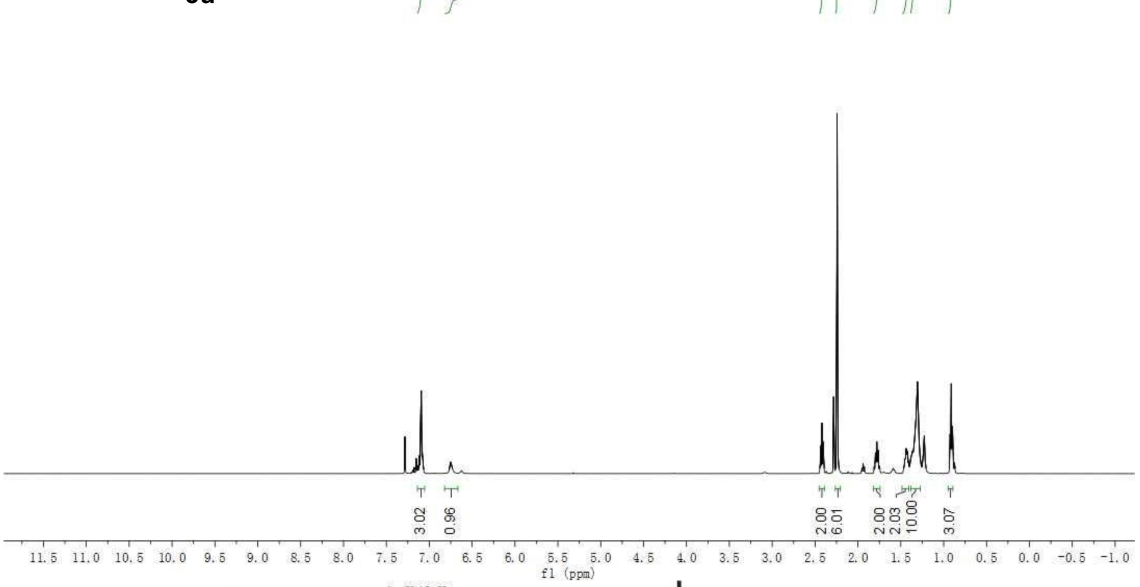

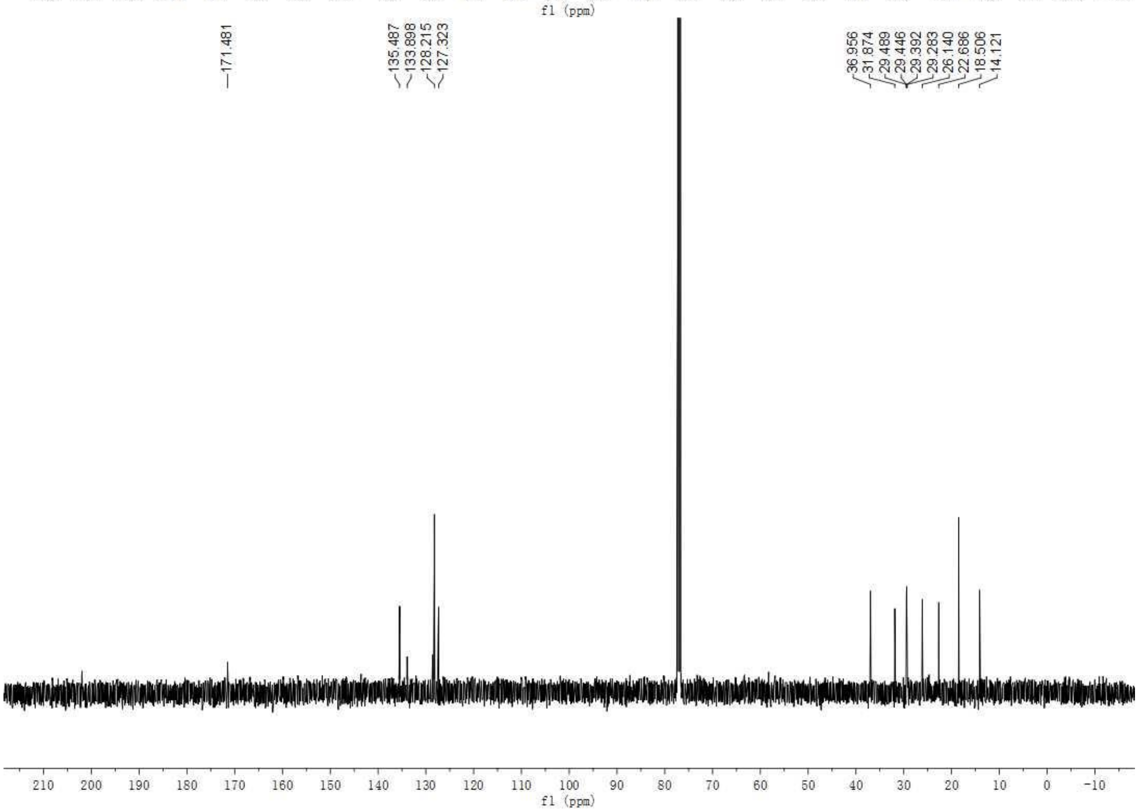

Supplementary Figure 31. ^{1}H and ^{13}C NMR spectra of 3u

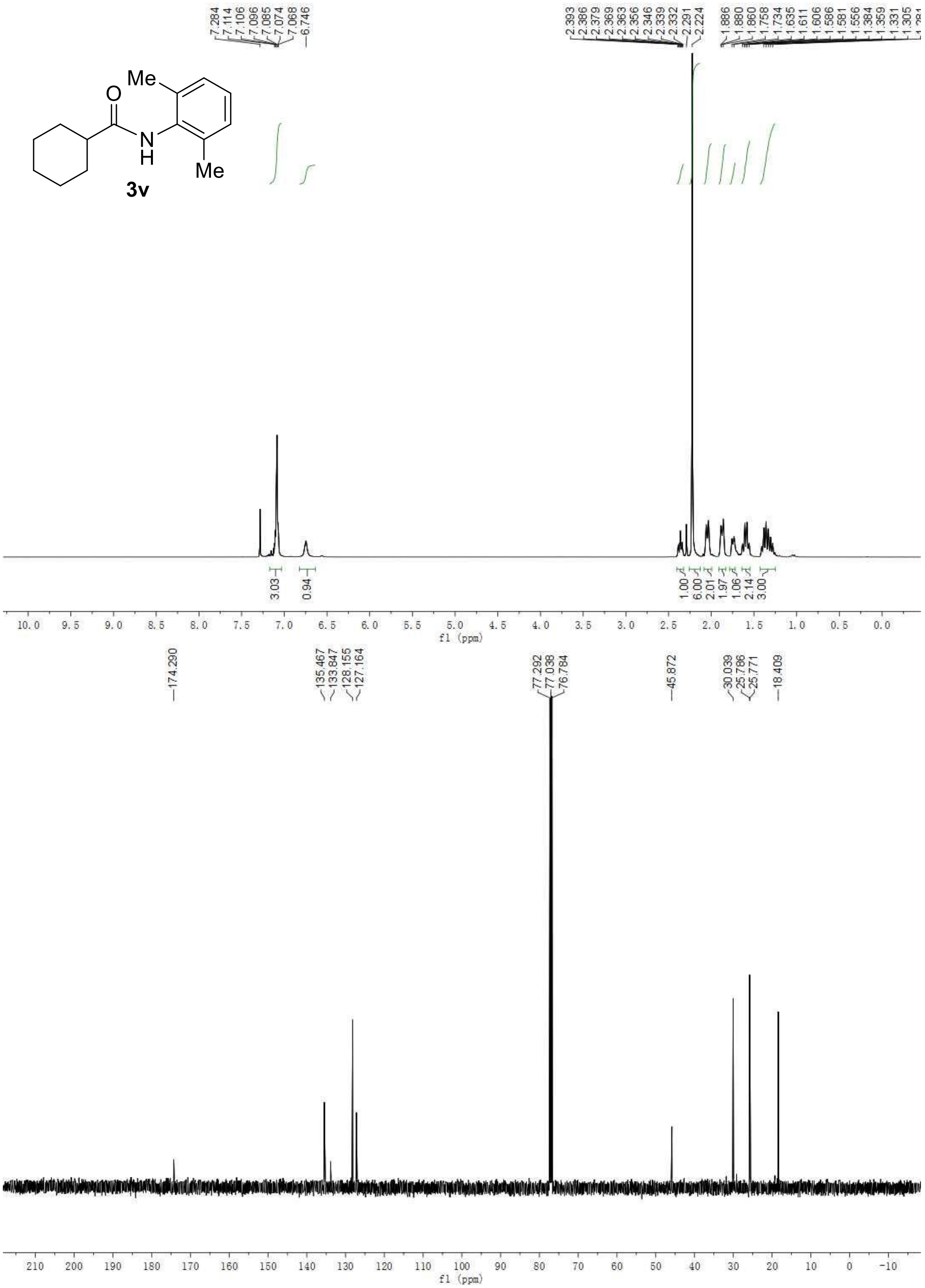

Supplementary Figure 32. ^{1}H and ^{13}C NMR spectra of 3v

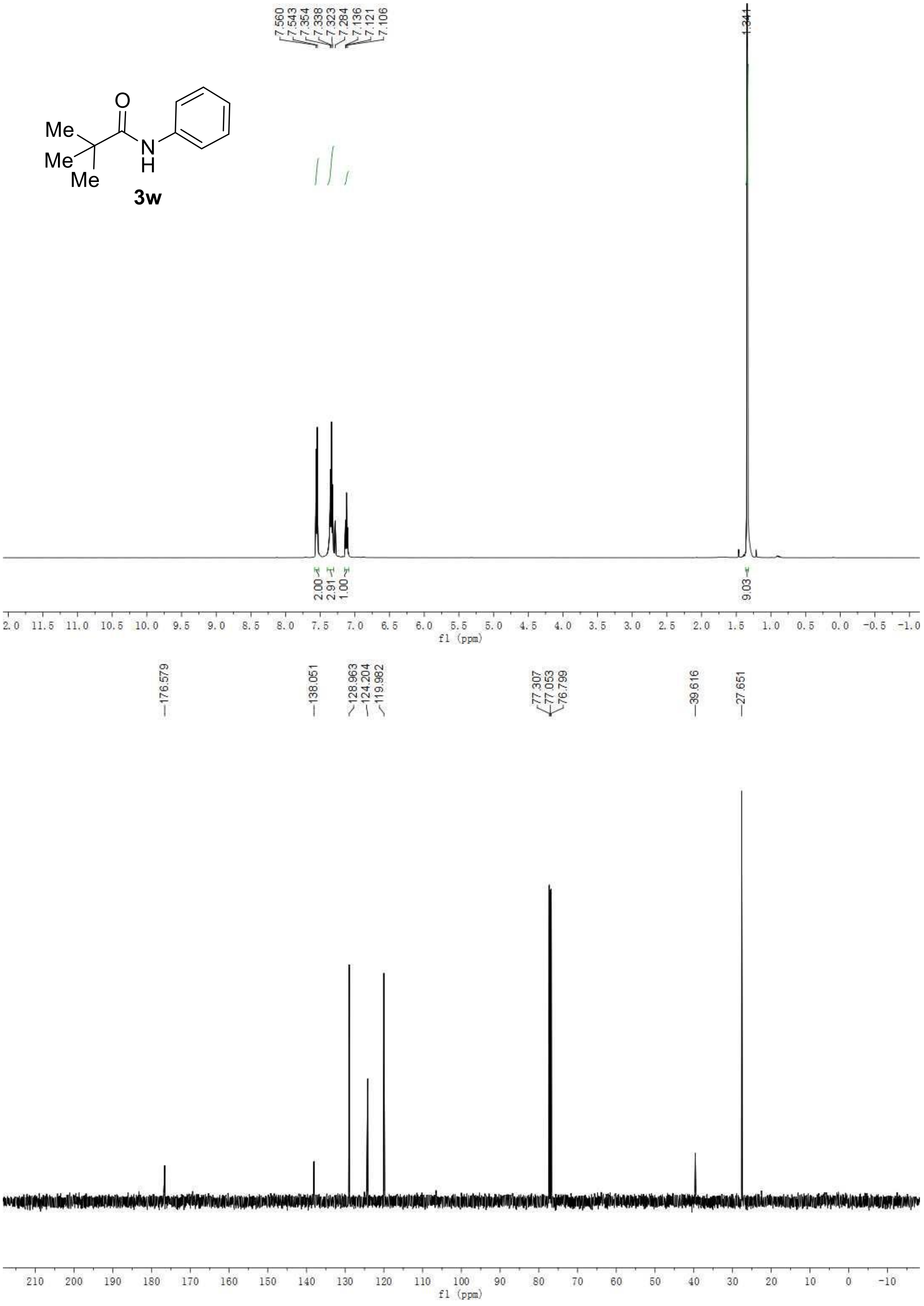

Supplementary Figure 33. ^{1}H and ^{13}C NMR spectra of 3w

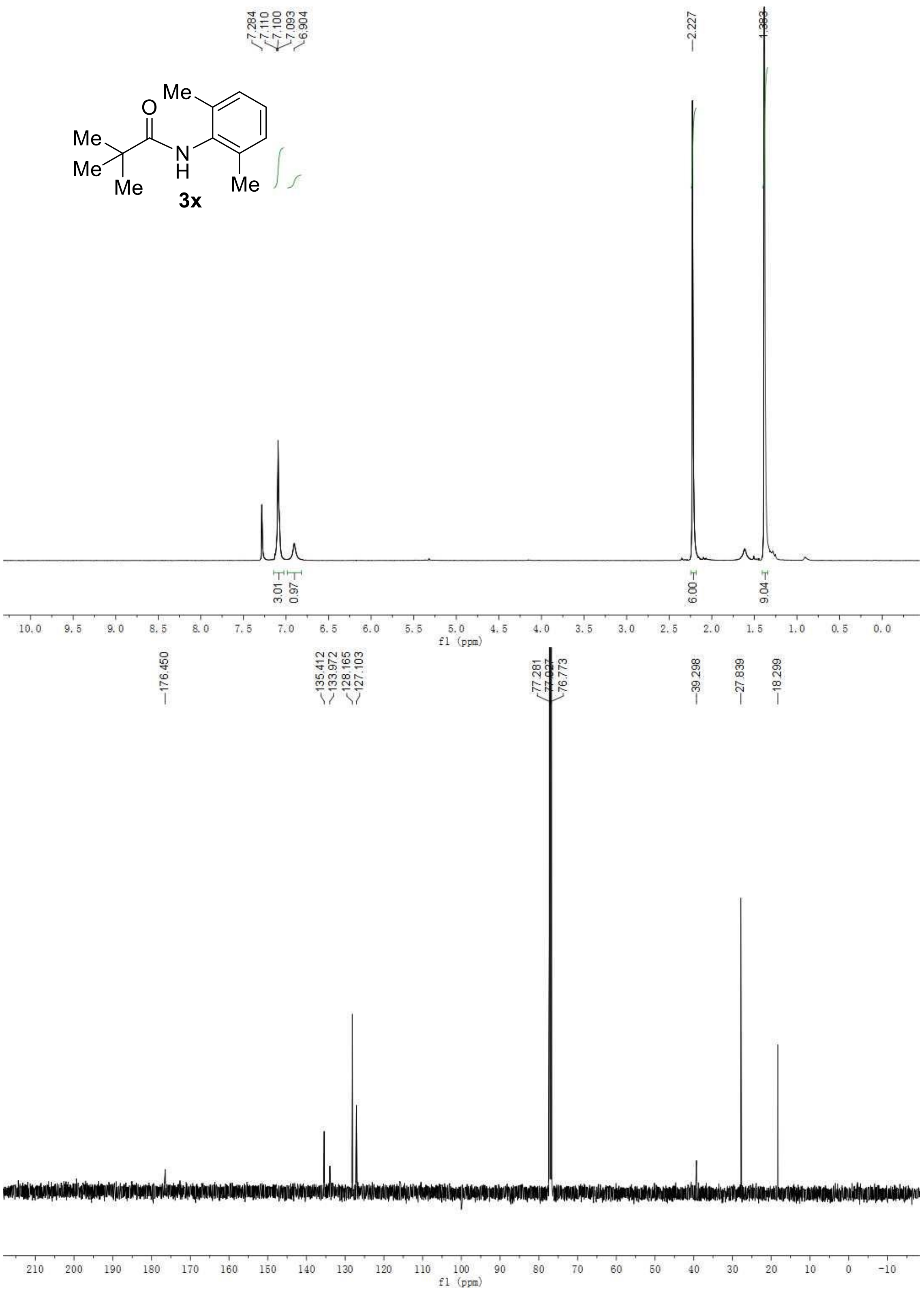

Supplementary Figure 34. ¹H and ¹³C NMR spectra of 3x

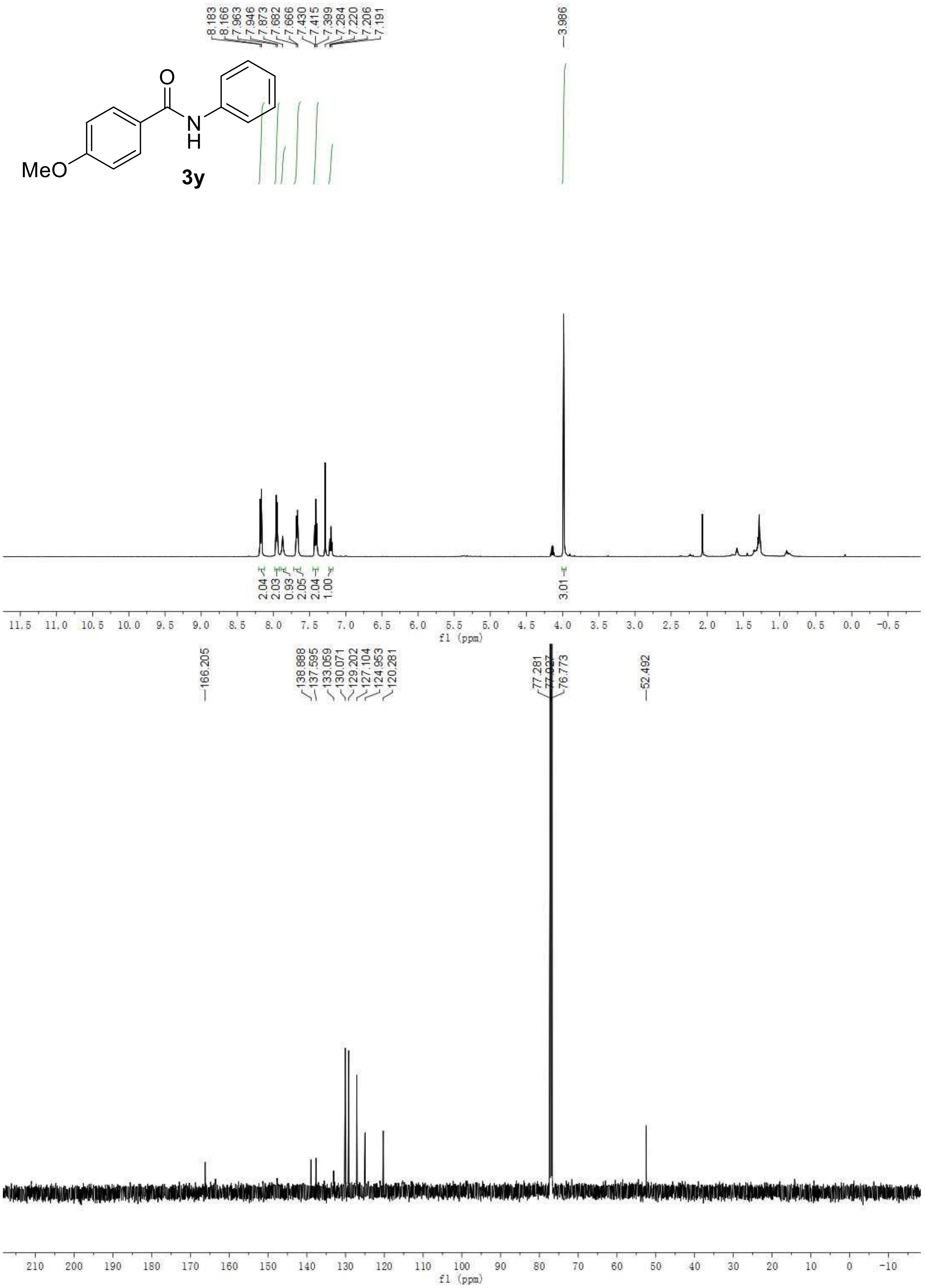

Supplementary Figure 35. ^{1}H and ^{13}C NMR spectra of 3y

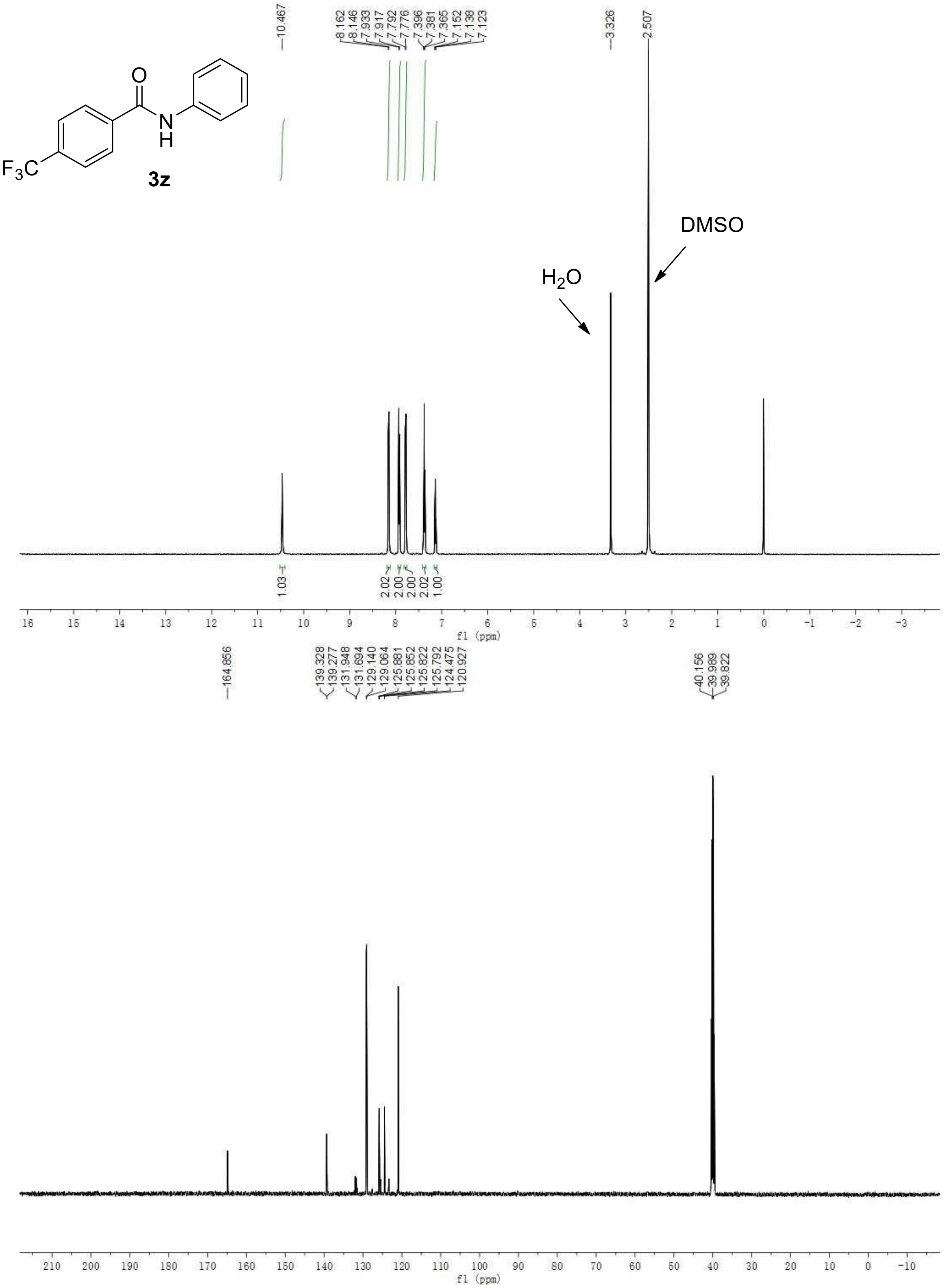

Supplementary Figure 36. ^{1}H and ^{13}C NMR spectra of 3z

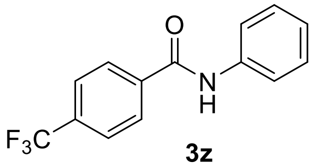

3z

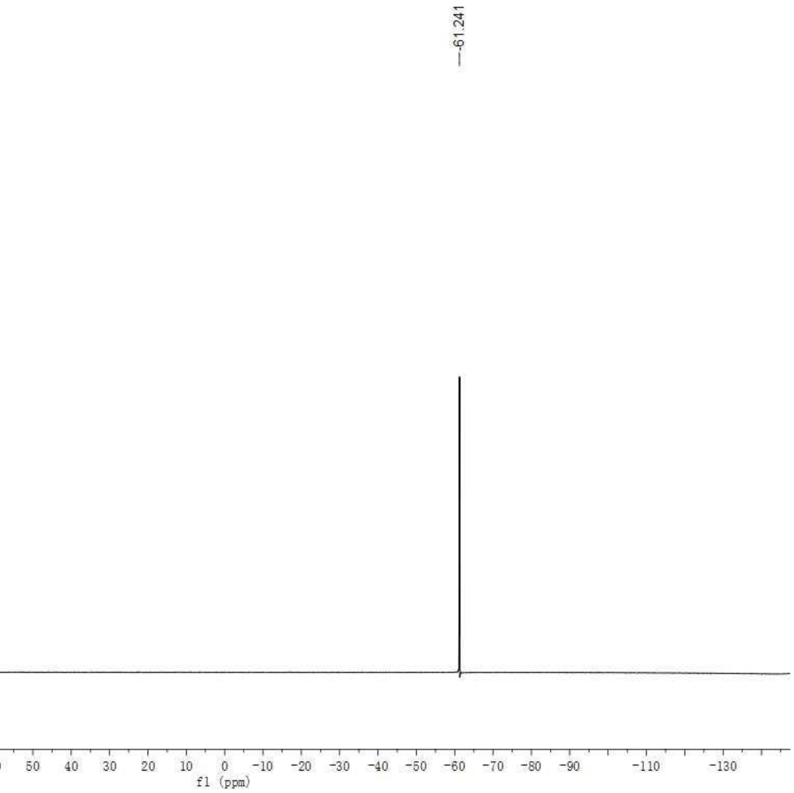

Supplementary Figure 37. ^{19}F NMR spectra of 3z

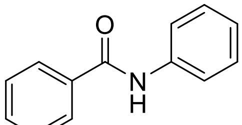

3aa

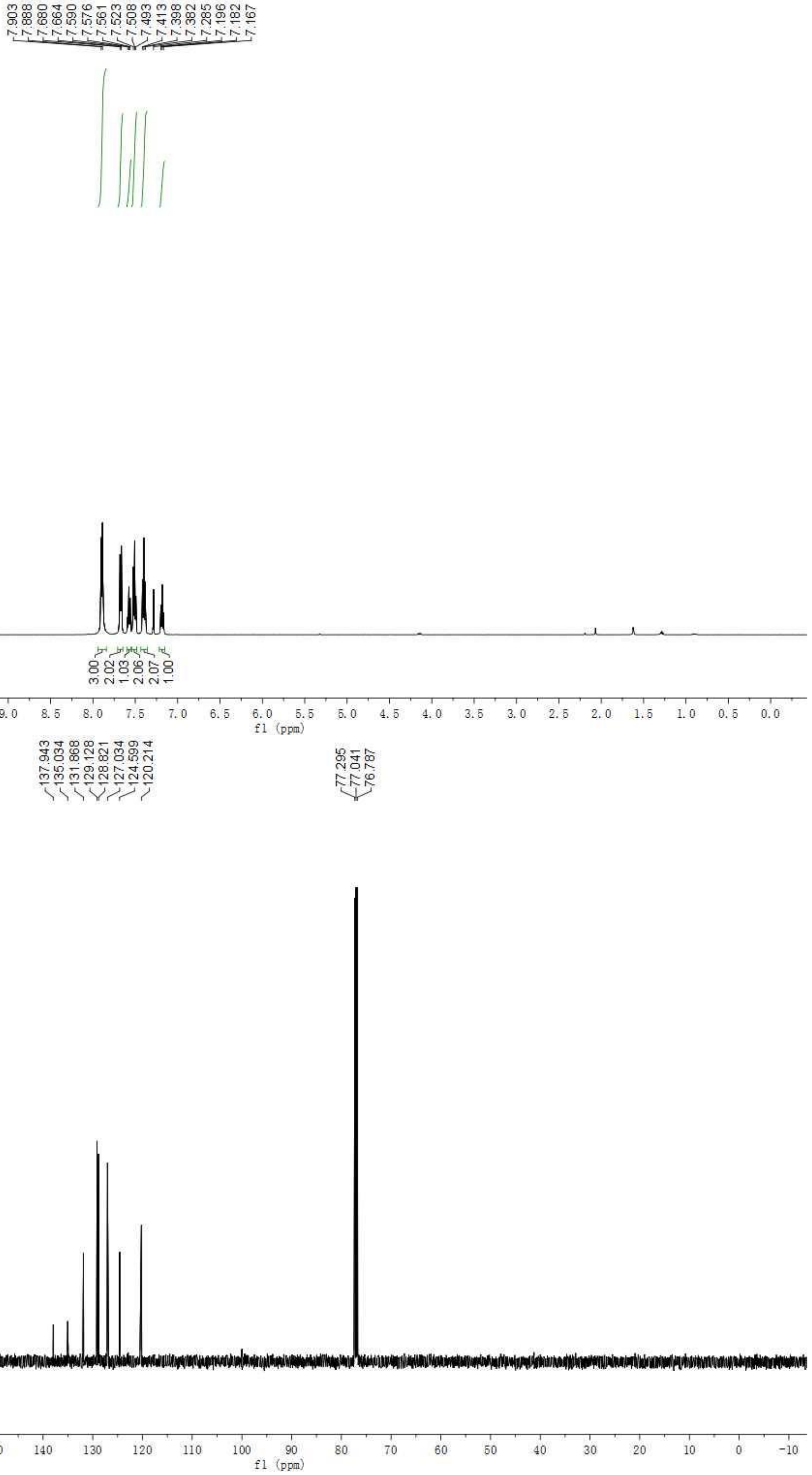

Supplementary Figure 38. ^{1}H and ^{13}C NMR spectra of 3aa

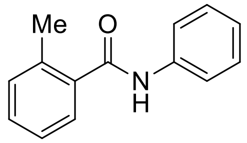

3ab

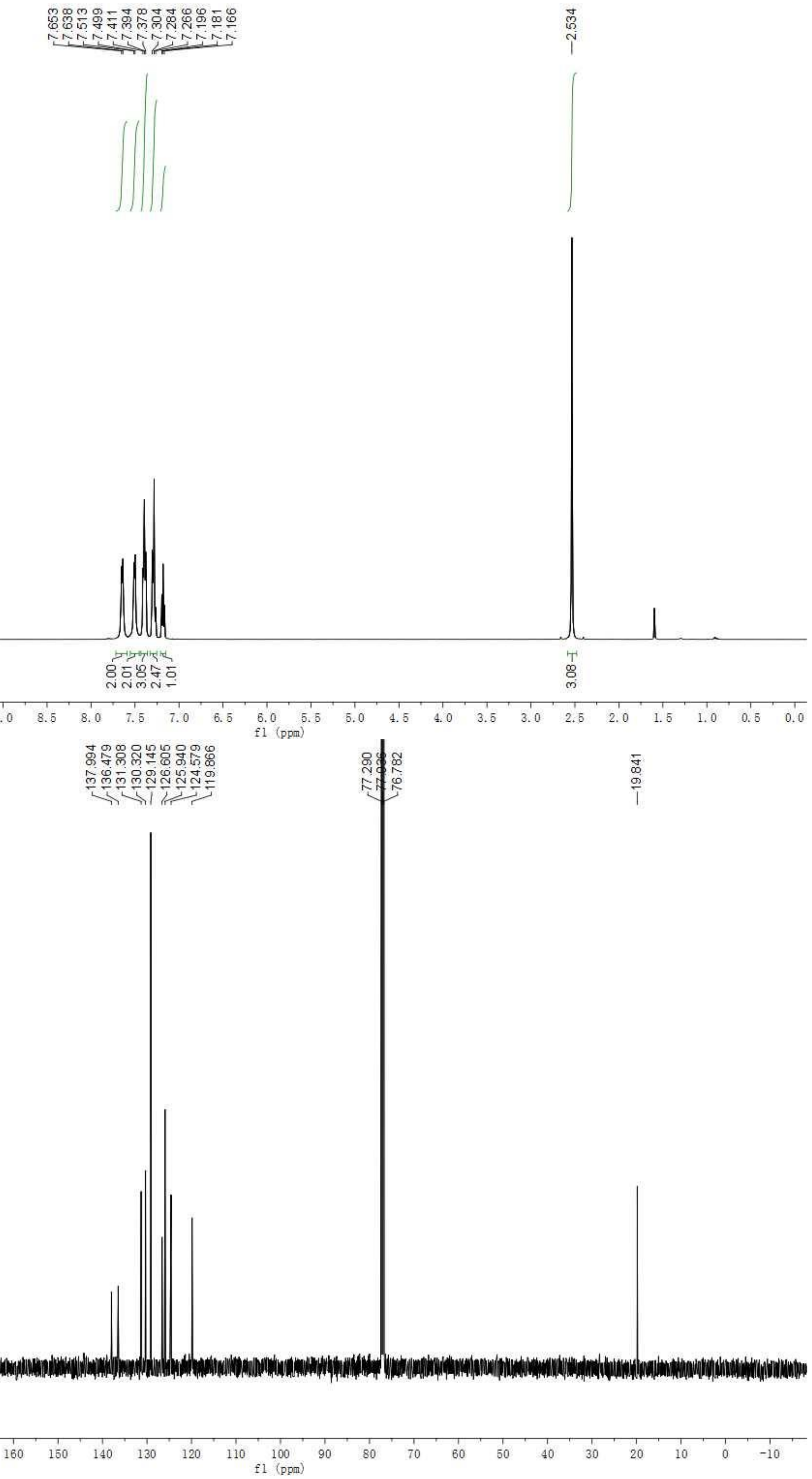

Supplementary Figure 39. ^{1}**H and** ^{13}**C NMR spectra of 3ab**

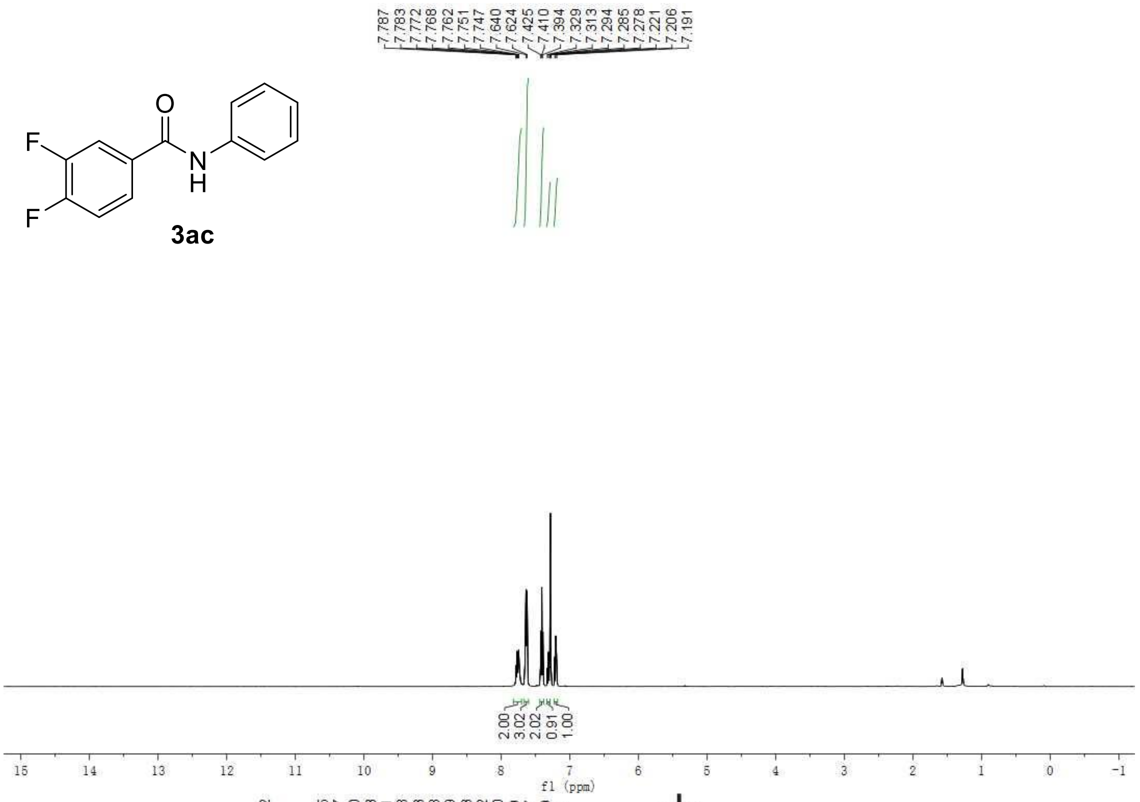

3ac

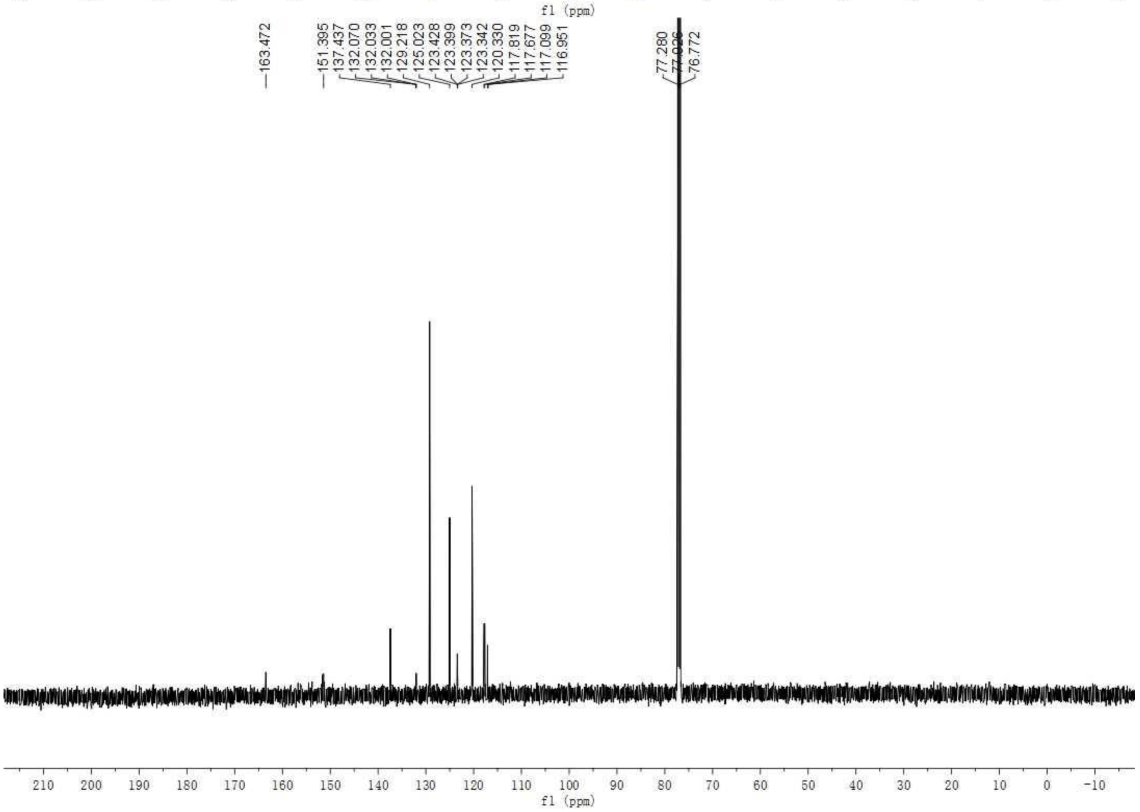

Supplementary Figure 40. ^{1}H and ^{13}C NMR spectra of 3ac

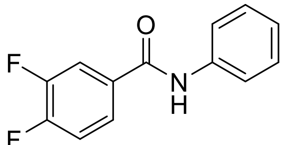

3ac

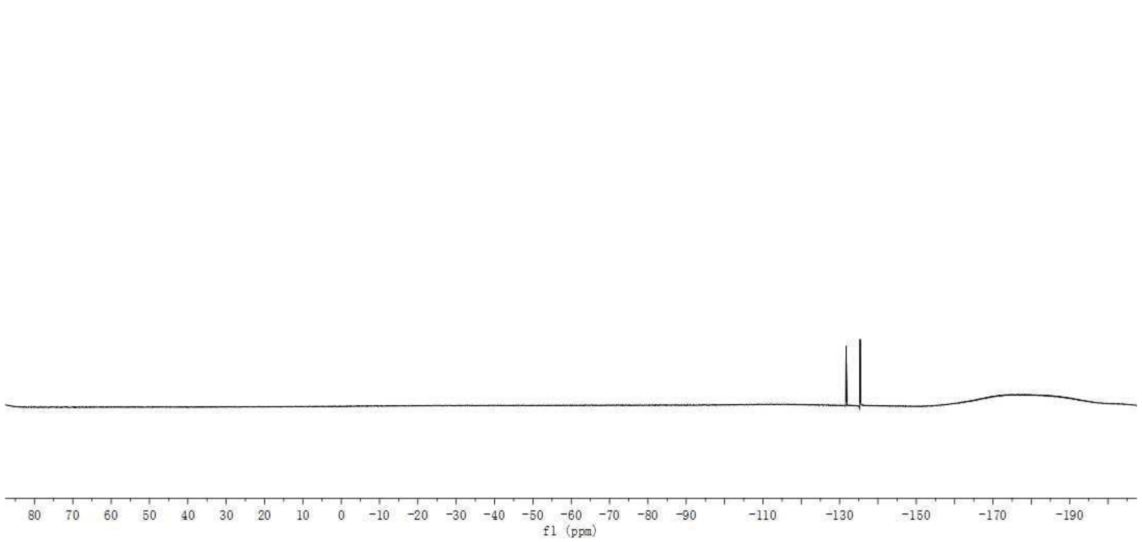

Supplementary Figure 41. ^{19}F NMR spectra of 3ac

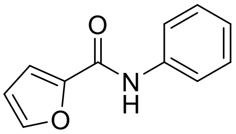

3ad

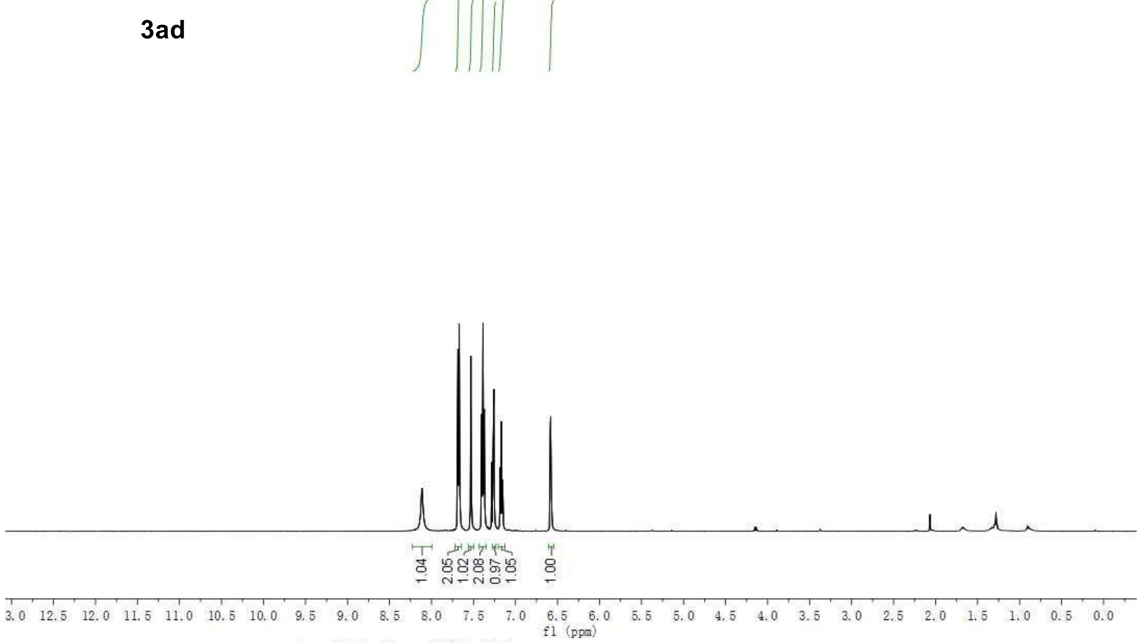

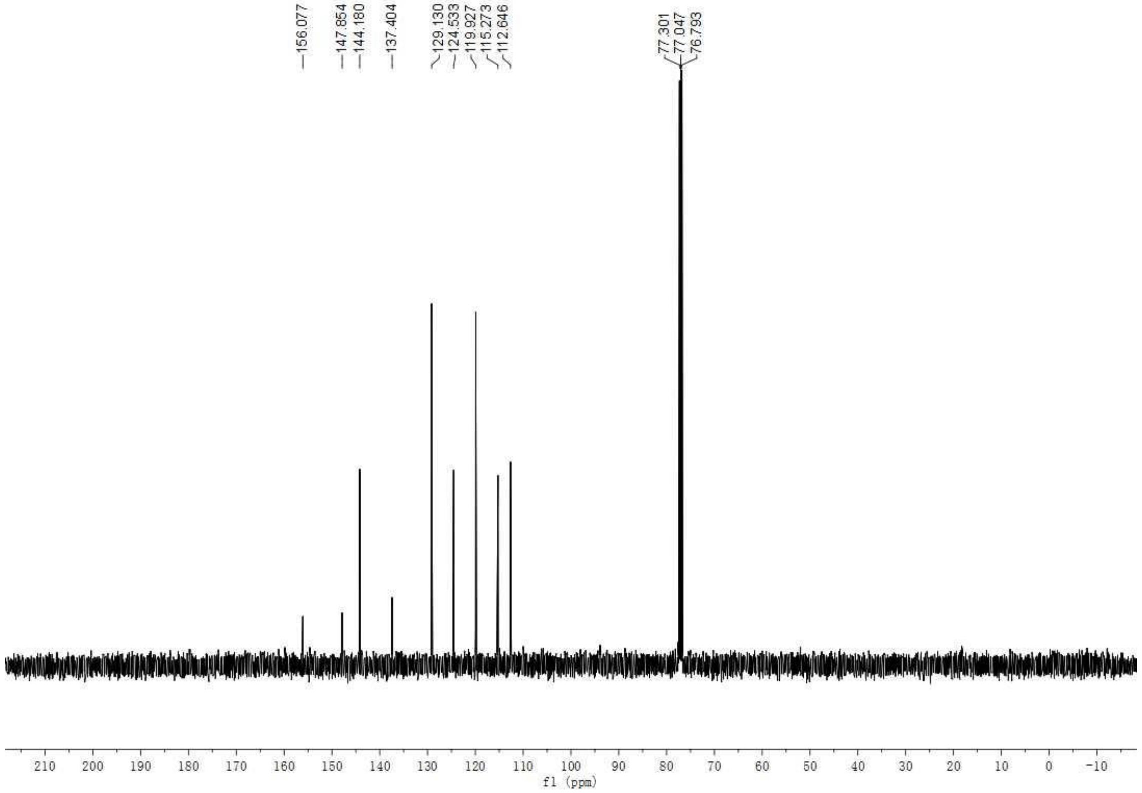

Supplementary Figure 42. ^{1}H and ^{13}C NMR spectra of 3ad

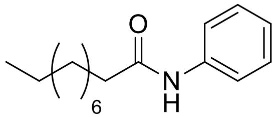

3ae

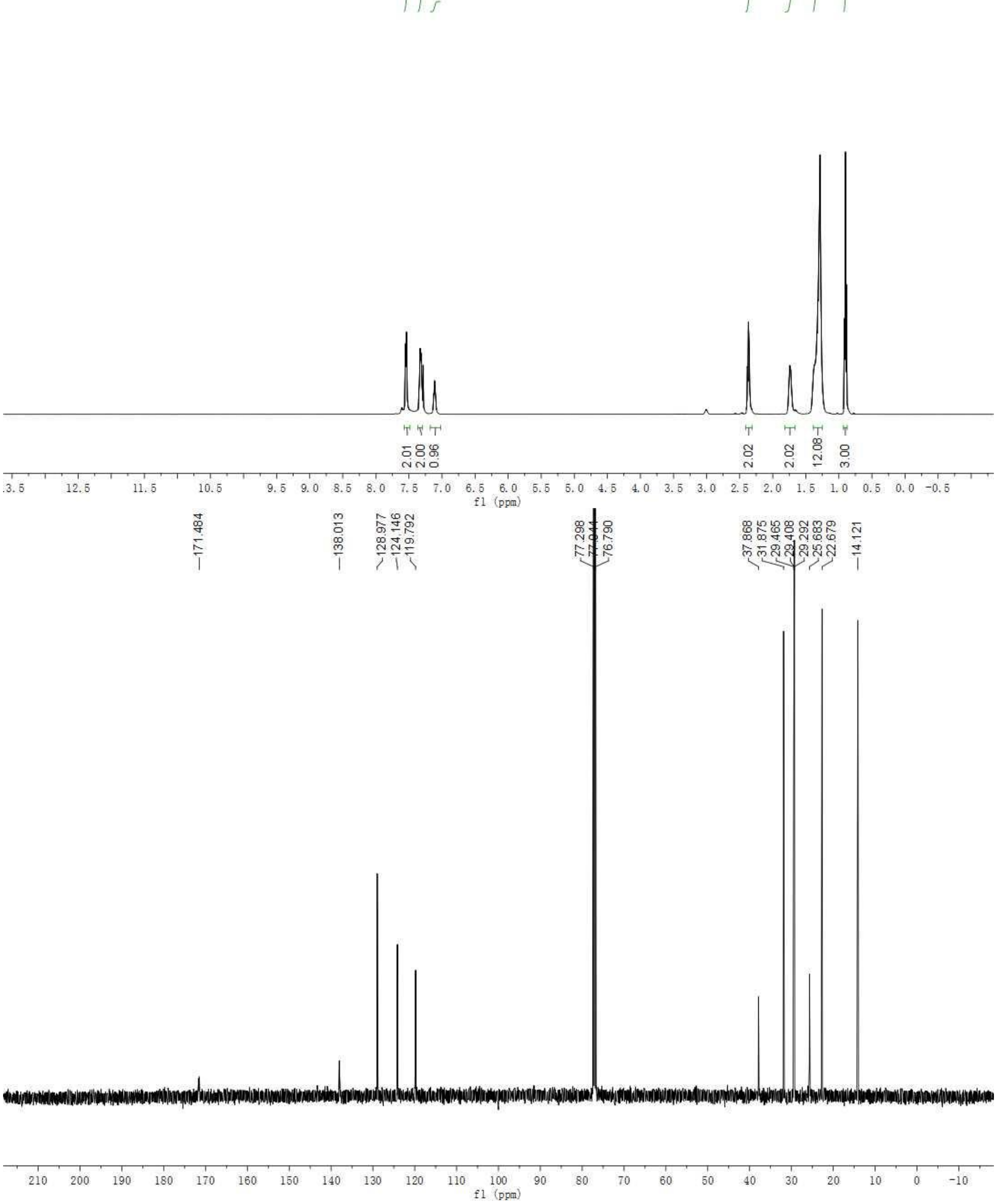

Supplementary Figure 43. ^{1}H and ^{13}C NMR spectra of 3ae

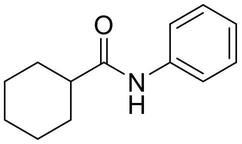

3af

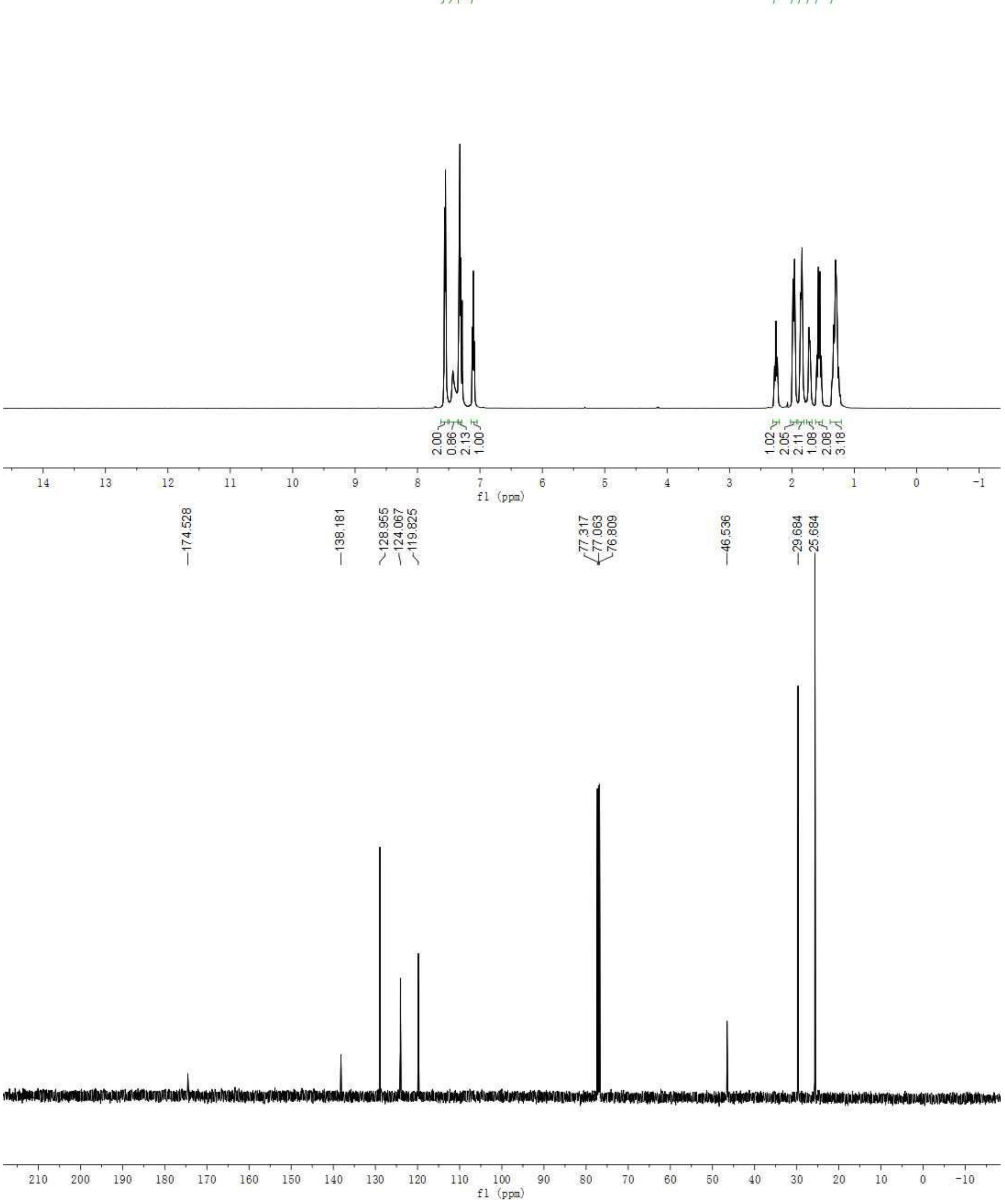

Supplementary Figure 44. ^{1}H and ^{13}C NMR spectra of 3af

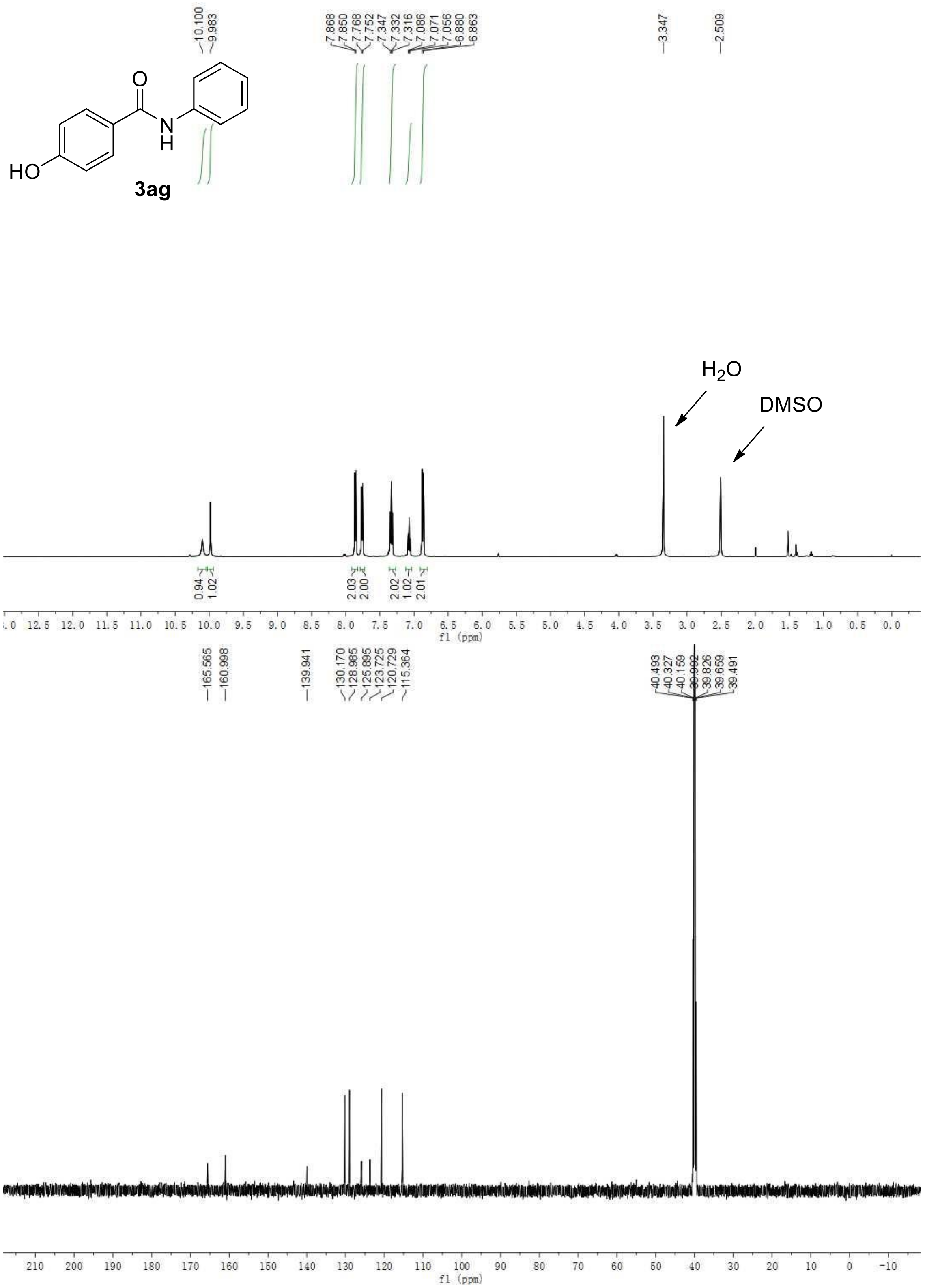

Supplementary Figure 45. ^{1}H and ^{13}C NMR spectra of 3ag

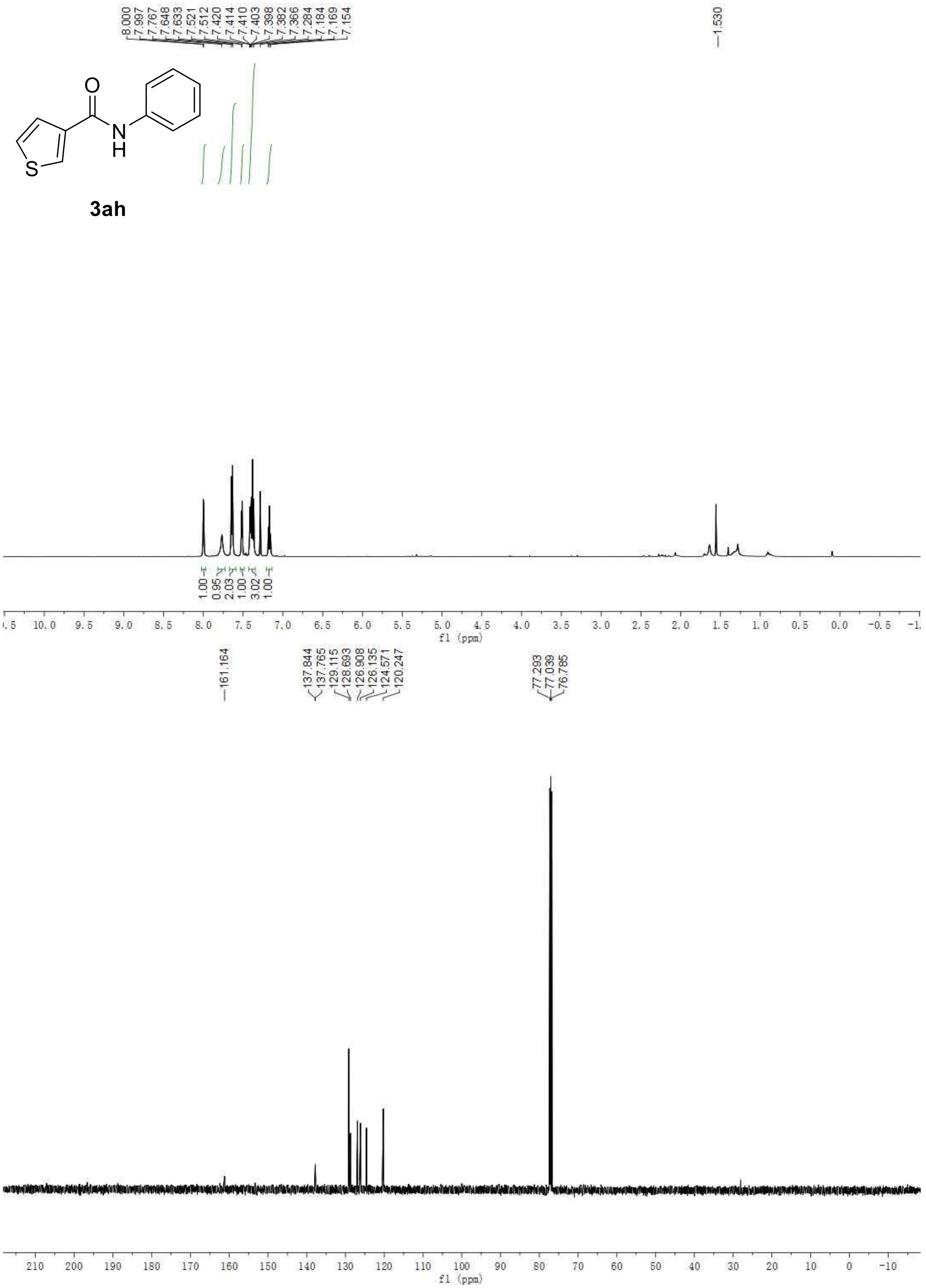

Supplementary Figure 46. ^{1}H and ^{13}C NMR spectra of 3ah

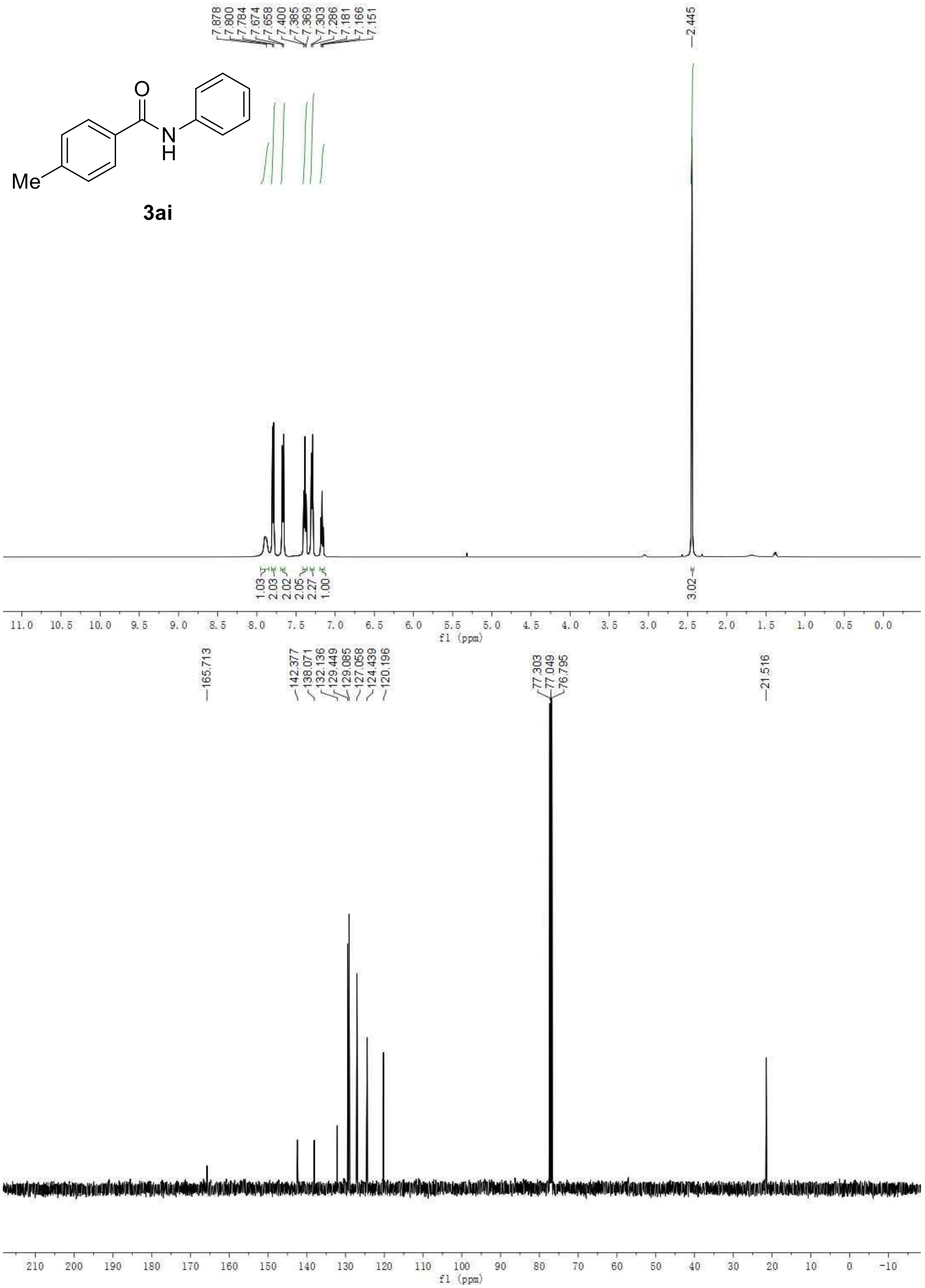

Supplementary Figure 47. ^{1}H and ^{13}C NMR spectra of 3ai

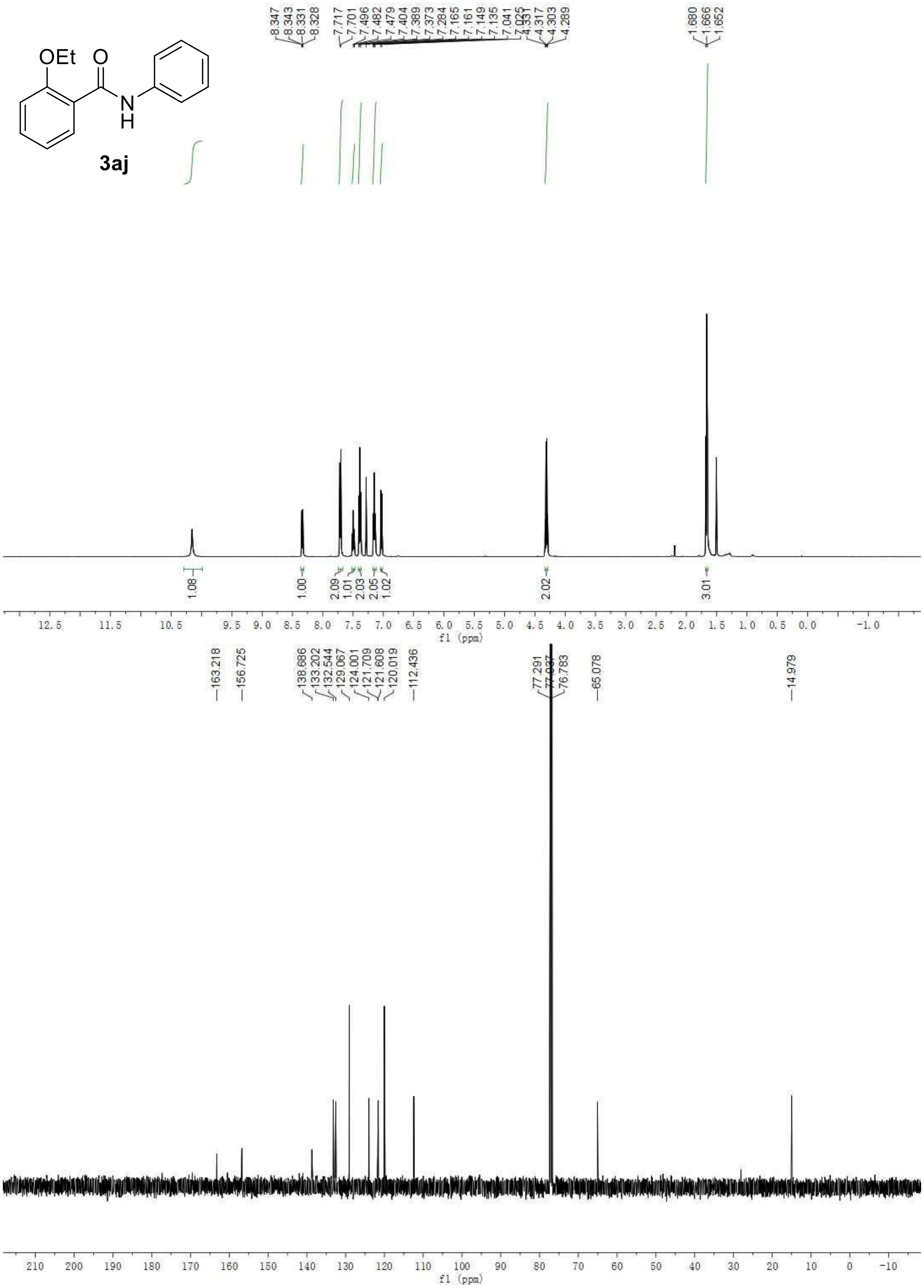

Supplementary Figure 48. ^{1}H and ^{13}C NMR spectra of 3aj

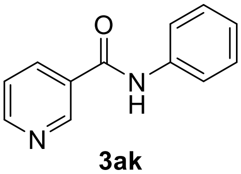

3ak

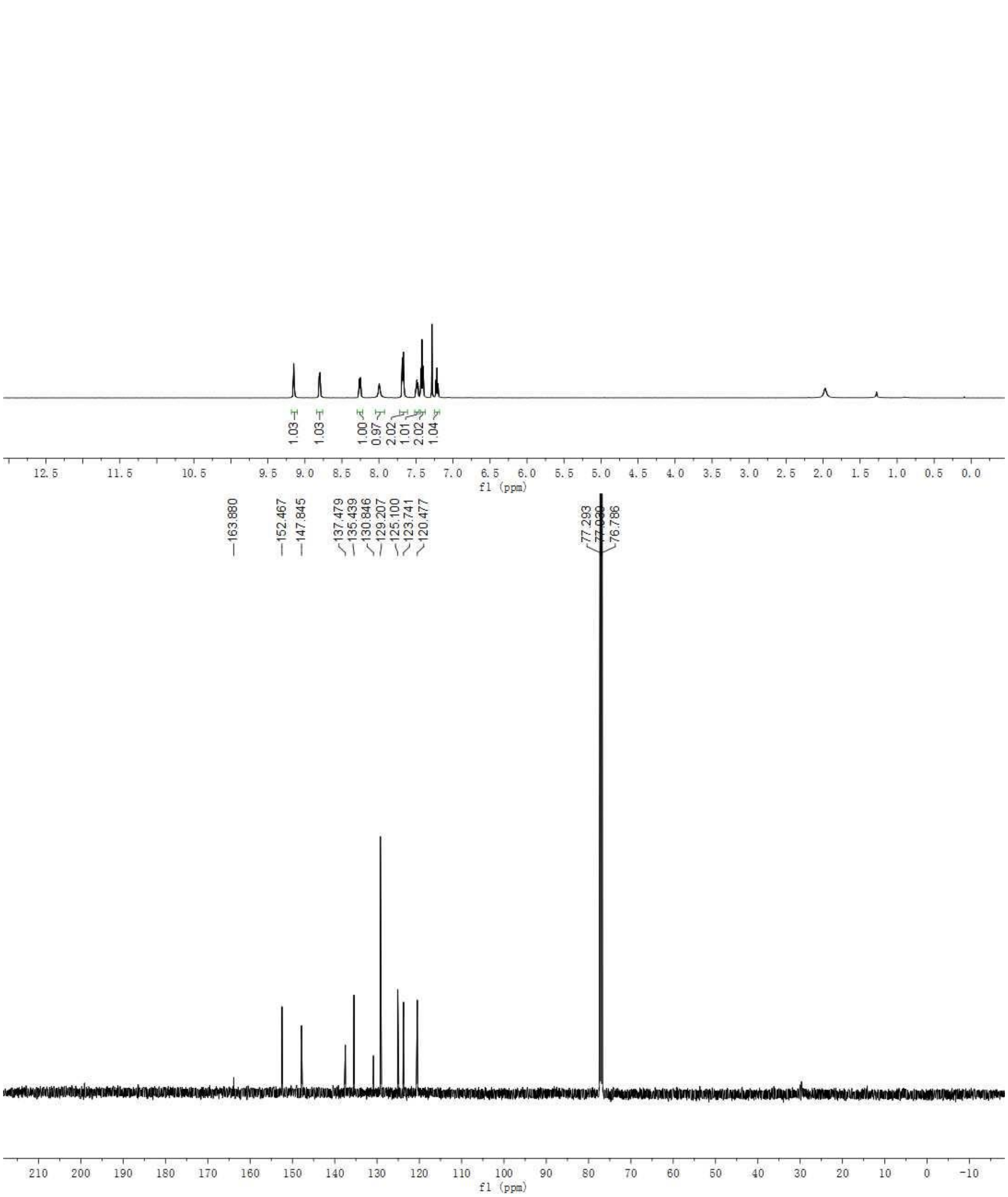

Supplementary Figure 49. ^{1}H and ^{13}C NMR spectra of 3ak

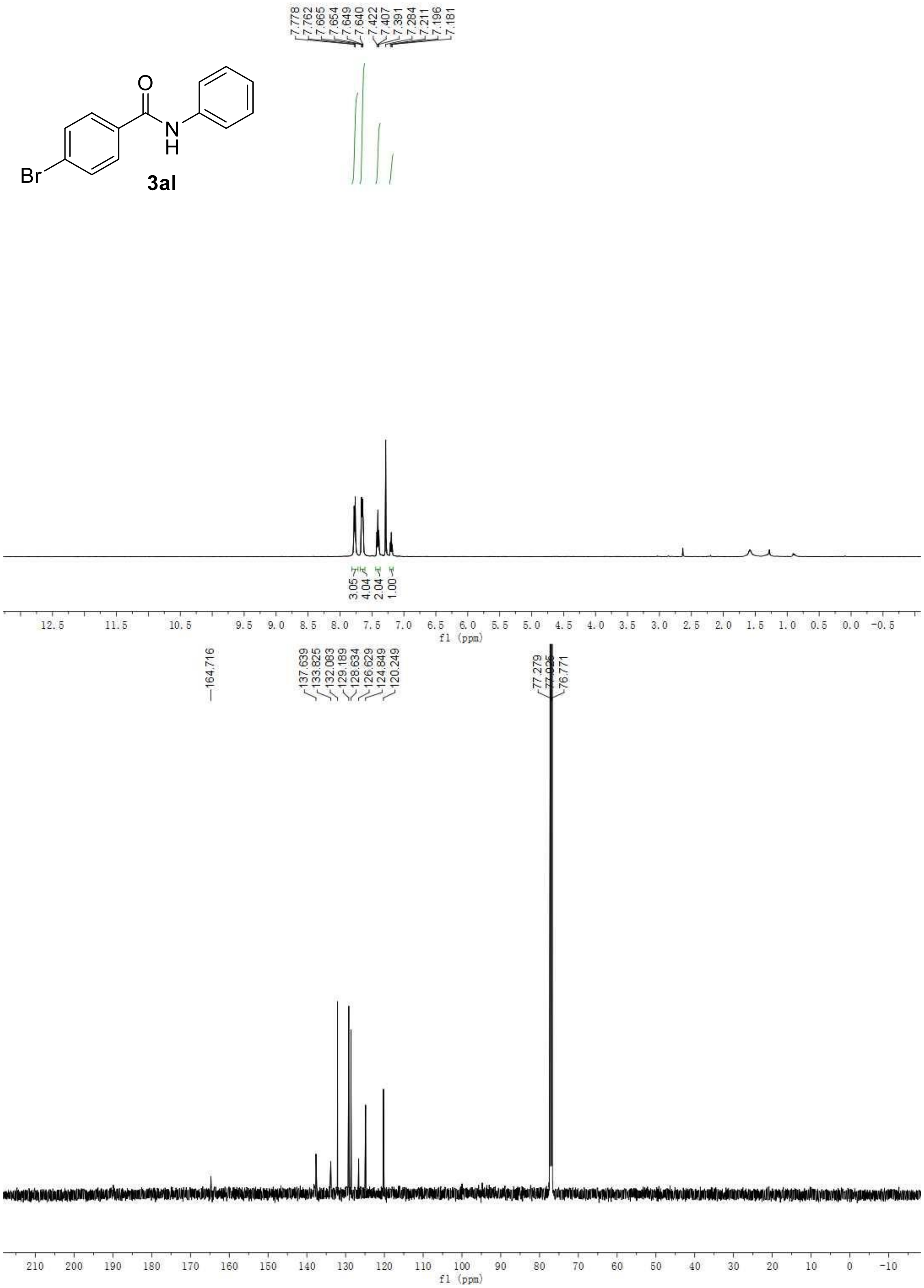

Supplementary Figure 50. ^{1}H and ^{13}C NMR spectra of 3al

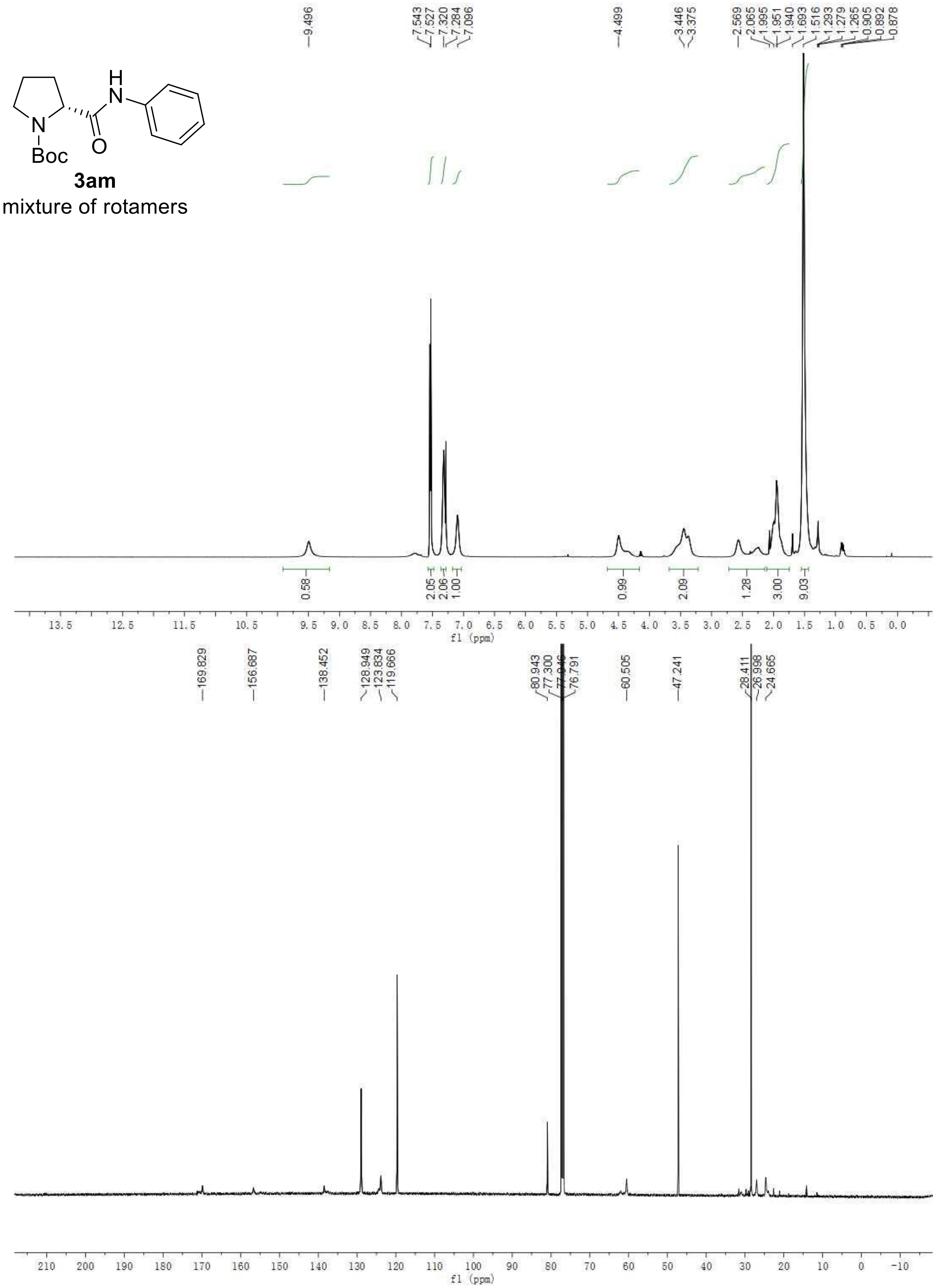

Supplementary Figure 51. ^{1}H and ^{13}C NMR spectra of 3am

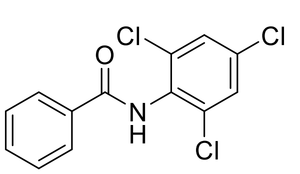

3an

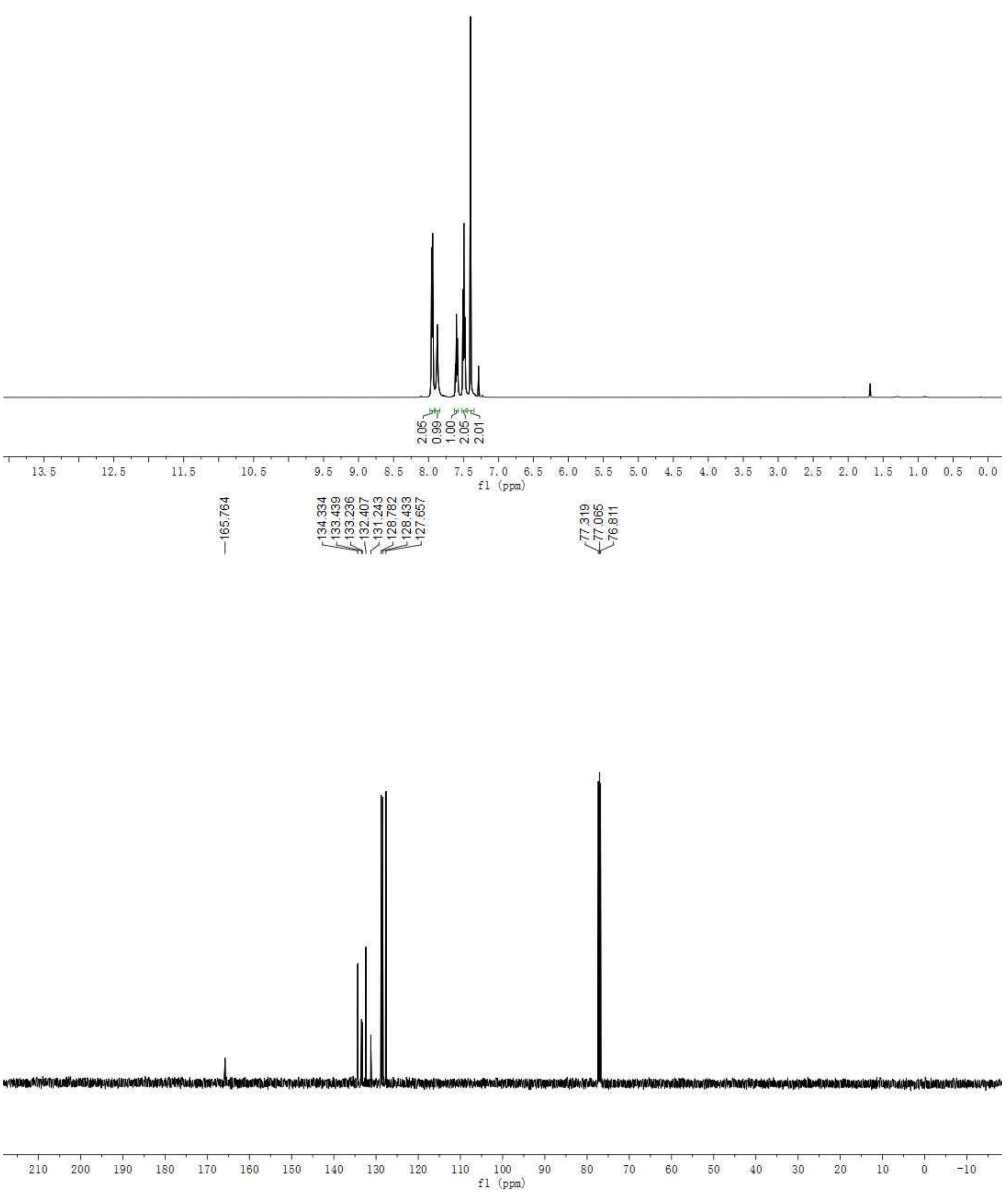

Supplementary Figure 52. ^{1}H and ^{13}C NMR spectra of 3an

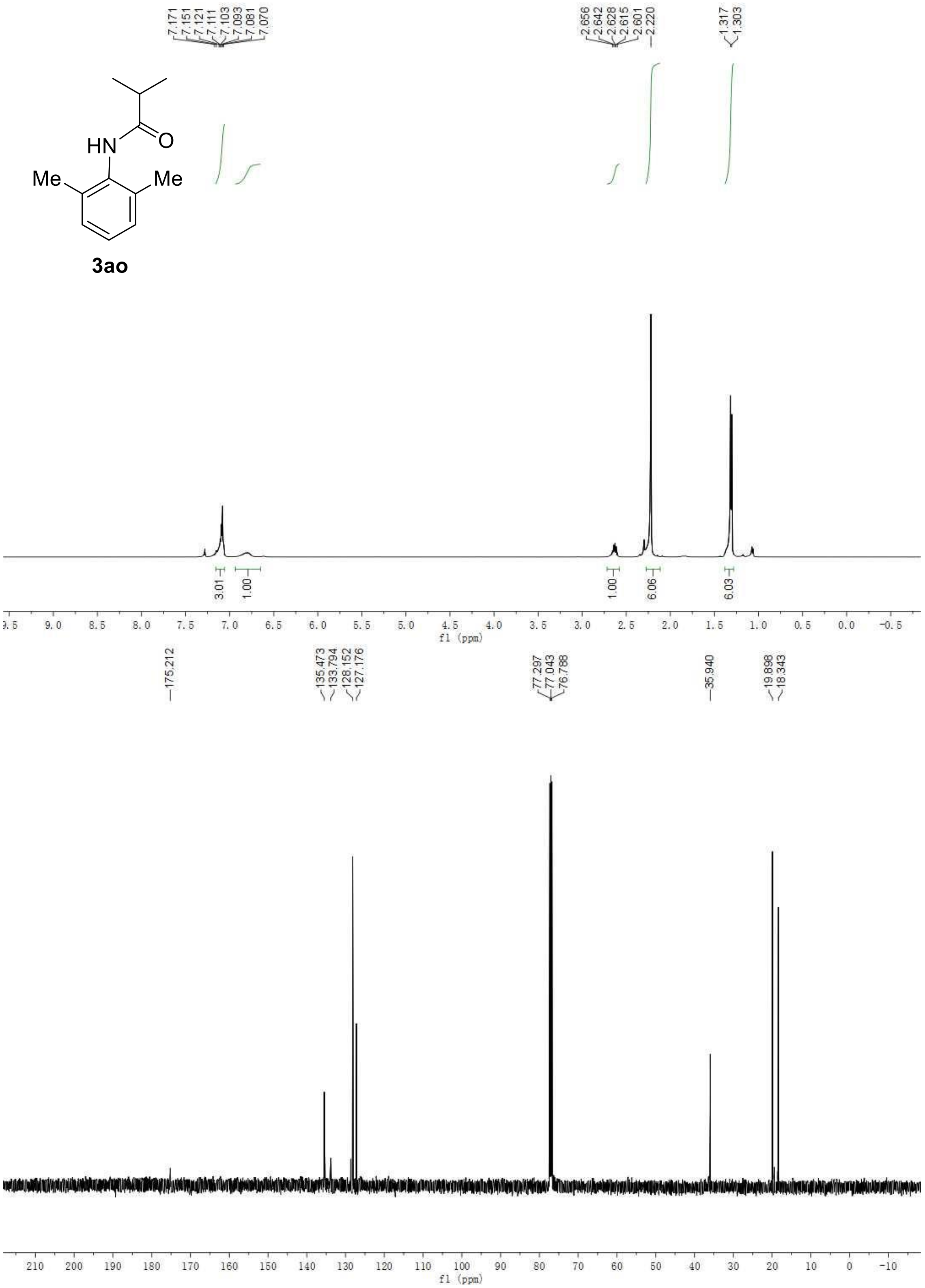

Supplementary Figure 53. ^{1}H and ^{13}C NMR spectra of 3ao

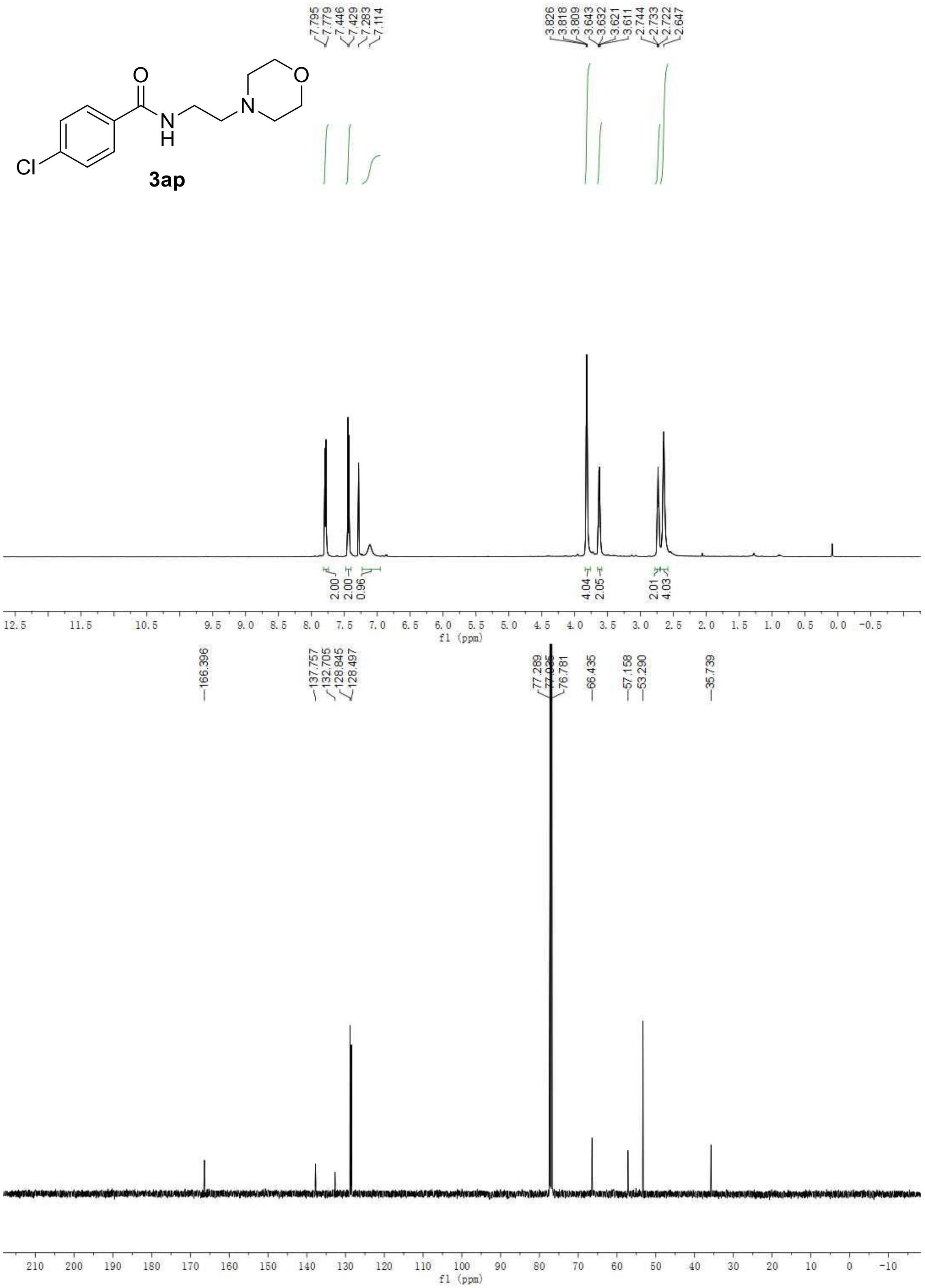

Supplementary Figure 54. ^{1}H and ^{13}C NMR spectra of 3ap

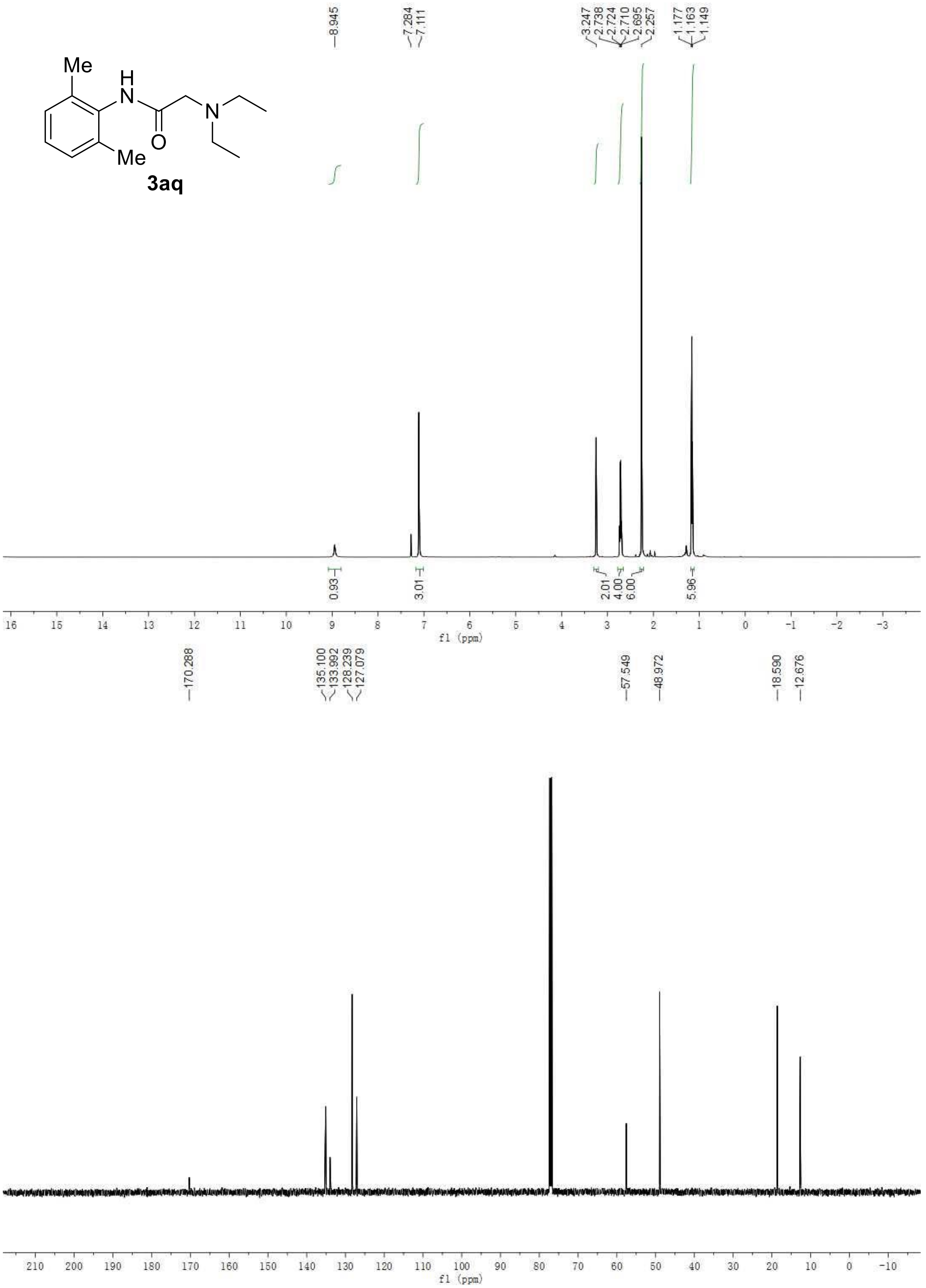

Supplementary Figure 55. ^{1}H and ^{13}C NMR spectra of 3aq

Supplementary References

1. Lei, P. *et al*. Suzuki–Miyaura cross-coupling of amides and esters at room temperature: correlation with barriers to rotation around C–N and C–O bonds. *Chem. Sci.* **8**, 6525–6530 (2017).
2. Dander, J. E., Baker, E. L. & Garg, N. K. Nickel-catalyzed transamidation of aliphatic amide derivatives. *Chem. Sci.* **8**, 6433–6438 (2017).
3. Wu, H. *et al*. Fluoride-catalyzed esterification of amides. *Chem. Eur. J.* **24**, 3444–3447 (2018).
4. Liu, Y., Shi, S., Achtenhagen, M., Liu, R. & Szostak, M. Metal-free transamidation of secondary amides via selective N–C cleavage under mild conditions. *Org. Lett.* **19**, 1614–1617 (2017).
5. Liu, C. *et al*. Palladium-catalyzed Suzuki–Miyaura cross-coupling of N-mesyl amides by N–C cleavage: electronic effect of the mesyl group. *Org. Lett.* **19**, 1434–1437 (2017).
6. Meng, G., Szostak, R. & Szostak, M. Suzuki–Miyaura cross-coupling of N-acylpyrroles and pyrazoles: planar, electronically activated amides in catalytic N–C cleavage. *Org. Lett.* **19**, 3596–3599 (2017).
7. Halima, T. B. *et al*. Palladium-catalyzed Suzuki-Miyaura coupling of aryl esters. *J. Am. Chem. Soc.* **139**, 1311–1318 (2017).
8. Muto, K., Yamaguchi, J., Musaev, D. G. & Itami, K. Decarbonylative organoboron cross-coupling of esters by nickel catalysis. *Nat. Commun.* **6**, 7508 (2015).
9. Yue, H. *et al*. Catalytic ester and amide to amine interconversion: nickel-catalyzed decarbonylative amination of esters and amides by C–O and C–C bond activation. *Angew. Chem. Int. Ed.* **56**, 4282–4285 (2017).
10. Kim, D. S., Park, W. J., Lee, C. H. & Jun, C. H. Hydroesterification of alkenes with sodium formate and alcohols promoted by cooperative catalysis of $Ru_3(CO)_{12}$ and 2-pyridinemethanol. *J. Org. Chem.* **79**, 12191–12196 (2014).
11. Carle, M. S., Shimokura, G. K. & Murphy, G. K. Iodobenzene dichloride in the esterification and amidation of carboxylic acids: in-situ synthesis of Ph_3PCl_2. *Eur. J. Org. Chem.* 3930–3933 (2016).

12. Kwon, E. M., Kim, C. G., Goh, A. R., Park, J. & Jun, J. G. Preparation of benzoyloxy benzophenone derivatives and their inhibitory effects of icam-1 expression. *Bull. Korean. Chem. Soc.* **33**, 1939–1944 (2012).
13. Hua, X., Masson-Makdissi, J., Sullivan, R. J. & Newman, S. G. Inherent vs apparent chemoselectivity in the Kumada–Corriu cross-coupling reaction. *Org. Lett.* **18**, 5312–5315 (2016).
14. Murie, V. E. *et al*. Acetaminophen Prodrug: Microwave-assisted synthesis and in vitro metabolism evaluation by mass spectrometry. *J. Braz. Chem. Soc.* **27**, 1121–1128 (2016).
15. Ragnarsson, U., Grehn, L., Monteiro, L. S. & Maia, H. L. S. Mild two-step conversion of primary amides to alcohols by reduction of acylimidodicarbonates with sodium borohydride. *Synlett* 2386–2388 (2003).
16. Meng, G., Shi, S. & Szostak, M. Palladium-catalyzed Suzuki–Miyaura cross-coupling of amides via site-selective N–C bond cleavage by cooperative catalysis. *ACS Catal.* **6**, 7335–7339 (2016).
17. Meng, G. & Szostak, M. Site-selective C–H/C–N activation by cooperative catalysis: primary amides as arylating reagents in directed C–H arylation. *ACS Catal.* **7**, 7251–7256 (2017).
18. Lei, P., Meng, G. & Szostak, M. General method for the Suzuki-Miyaura cross-coupling of amides using commercially available, air- and moisture-stable palladium/nhc (nhc = N-heterocyclic carbene) complexes. *ACS Catal.* **7**, 1960–1965 (2017).
19. Hie, L. *et al*. Conversion of amides to esters by the nickel-catalysed activation of amide C–N bonds. *Nature* **524**, 79–83 (2015).
20. Li, X. & Zou, G. Acylative Suzuki coupling of amides: acyl-nitrogen activation via synergy of independently modifiable activating groups. *Chem. Commun.* **51**, 5089–5092 (2015).
21. Asahara, H., Arikiyo, K. & Nishiwaki, N. Development of variously functionalized nitrile oxides. *Beilstein J. Org. Chem.* **11**, 1241–1245 (2015).
22. Meng, G., Shi, S., Lalancette, R., Szostak, R. & Szostak, M. Reversible twisting of primary amides via ground state N–C(O) destabilization: highly twisted rotationally inverted acyclic amides. *J. Am. Chem. Soc.* **140**, 727–734 (2018).

23. Perron-Sierra, F. *et al*. Substituted benzocyloheptenes as potent and selective αv-integrin antagonists. *Bioorg. Med. Chem. Lett.* **12**, 3291–3296 (2002).
24. Ennis, M. D. *et al*. Isochroman-6-carboxamides as highly selective 5-ht1d agonists: potential new treatment for migraine without cardiovascular side effects. *J. Med. Chem.* **41**, 2180–2183 (1998).
25. Miller, D. D. *et al*. 3,5-Disubstituted-indole-7-carboxamides: The discovery of a novel series of potent, selective inhibitors of IKK-β. *Bioorg. Med. Chem. Lett.* **21**, 2255–2258 (2011).
26. Takise, R., Muto, K. & Yamaguchi, J. Cross-coupling of aromatic esters and amides. *Chem. Soc. Rev.* **46**, 5864–5888 (2017).
27. Meng, G., Lei, P. & Szostak, M. A general method for two-step transamidation of secondary amides using commercially available, air- and moisture-stable palladium/nhc (N-heterocyclic carbene) complexes. *Org. Lett.* **19**, 2158–2161 (2017).
28. Halima, T. B., Vandavasi, J. K., Shkoor, M. & Newman, S. G. A cross-coupling approach to amide bond formation from esters. *ACS Catal.* **7**, 2176–2180 (2017).
29. Xiao, F., Liu, Y., Tang, C. & Deng, G. J. Peroxide-mediated transition-metal-free direct amidation of alcohols with nitroarenes. *Org. Lett.* **14**, 984–987 (2012).
30. Baker, E. L., Yamano, M. M., Zhou, Y., Anthony, S. M. & Garg, N. K. A two-step approach to achieve secondary amide transamidation enabled by nickel catalysis. *Nat. Commun.* **7**, 11554 (2016).
31. Jin, L. M. *et al*. Selective radical amination of aldehydic C(sp^2)–H bonds with fluoroaryl azides via Co(II)-based metalloradical catalysis: synthesis of N-fluoroaryl amides from aldehydes under neutral and nonoxidative conditions. *Chem. Sci.* **5**, 2422–2427 (2014).
32. Wybon, C. C. D. *et al*. Zn-catalyzed tert-butyl nicotinate-directed amide cleavage as a biomimic of metallo-exopeptidase activity. *ACS Catal.* **8**, 203–218 (2018).
33. Ho, B., Crider, A. M. & Stables, J. P. Synthesis and structure–activity relationships of potential anticonvulsants based on 2-piperidinecarboxylic acid and related pharmacophores. *Eur. J. Med. Chem.* **36**, 265–286 (2001).
34. Xi, X., Chen, T., Zhang, J. S. & Han, L. B. Efficient and selective hydrogenation of C–O bonds with a simple sodium formate catalyzed by nickel. *Chem. Commun.* **54**, 1521–1524 (2018).

35. Zhu, Y. P., Sergeyev, S., Franck, P., Orru, R. V. A. & Maes, B. U. W. Amine activation: synthesis of N-(hetero)arylamides from isothioureas and carboxylic acids. *Org. Lett.* **18**, 4602–4605 (2016).
36. Fan, W., Yang, Y., Lei, J., Jiang, Q. & Zhou, W. Copper-catalyzed N-benzoylation of amines via aerobic C–C bond cleavage. *J. Org. Chem.* **80**, 8782–8789 (2015).
37. Moocilac, P., Osman, I. A. & Gallagher, J. F. Short C–H F interactions involving the 2,5-difluorobenzene group: understanding the role of fluorine in aggregation and complex C–F/C–H disorder in a 2 × 6 isomer grid. *CrystEngComm* **18**, 5764–5776 (2016).
38. Shi, S. & Szostak, M. Pd–PEPPSI: a general Pd–NHC precatalyst for Buchwald–Hartwig cross-coupling of esters and amides (transamidation) under the same reaction conditions. *Chem. Commun.* **53**, 10584–10587 (2017).
39. Dander, J. E., Baker, E. L. & Garg, N. K. Nickel-catalyzed transamidation of aliphatic amide derivatives. *Chem. Sci.* **8**, 6433–6438 (2017).
40. Kumar, A. & Bishnoi, A. K. Nanoparticle mediated organic synthesis (NAMO-synthesis): CuI-NP catalyzed ligand free amidation of aryl halides. *RSC Adv.* **4**, 41631–41635 (2014).
41. Miura, T., Takahashi, Y. & Murakami, M. Rhodium-catalysed addition reaction of aryl- and alkenylboronic acids to isocyanates. *Chem. Commun.* 3577–3578 (2007).
42. Werbel, L. M., Hess, C. A. & Elslager, E. F. Potential antimalarial substances. amides of o-ethoxy- and p-isopropylbenzoic acids. *J. Med. Chem.* **10**, 508–509 (1967).
43. Rovira, M. *et al*. Orthogonal discrimination among functional groups in Ullmann-type C–O and C–N couplings. *J. Org. Chem.* **81**, 7315–7325 (2016).
44. Seo, H. A., Cho, Y. H., Lee, Y. S. & Cheon, C. H. Formation of amides from imines via cyanide-mediated metal-free aerobic oxidation. *J. Org. Chem.* **80**, 11993–11998 (2015).
45. Wojcik, P., Rosar, V., Gniewek, A., Milani, B. & Trzeciak, A. M. In situ generated Pd(0) nanoparticles stabilized by bis(aryl)acenaphthenequinone diimines as catalysts for aminocarbonylation reactions in water. *J. Mol. Cat. A: Chem.* **425**, 322–331 (2016).
46. Donnier-Marechal, M. *et al*. Synthesis and pharmacological evaluation of benzamide derivatives as potent and selective sigma-1 protein ligands. *Eur. J. Med. Chem.* **138**, 964–978 (2017).

47. Monbaliu, J. C. M. *et al*. Compact and integrated approach for advanced end-to-end production, purification, and aqueous formulation of lidocaine hydrochloride. S. *Org. Process Res. Dev.* **20**, 1347–1353 (2016).
48. Dudley, T. J. *et al*. Conformational analysis via calculations and NMR spectroscopy for isomers of the mono(imino)pyridine ligand, 2-{(2,6-Me_2-C_6H_3)NC(*i*-Pr)}C_5H_4N. *RSC Adv.* **2**, 6237–6244 (2012).
